# Global distribution and health impact of infectious disease outbreaks, 1996–2023: a worldwide retrospective analysis of World Health Organization emergency event reports

**DOI:** 10.7189/jogh.15.04151

**Published:** 2025-05-16

**Authors:** Qiao Liu, Min Liu, Wannian Liang, Xuanjun Li, Wenzhan Jing, Zhongdan Chen, Jue Liu

**Affiliations:** 1 Department of Epidemiology and Biostatistics, School of Public Health, Peking University, Beijing, China; 2 Ministry of Education, Key Laboratory of Epidemiology of Major Diseases, Peking University, Beijing, China; 3 Global Centre for Infectious Disease and Policy Research and Global Health and Infectious Diseases Group, Peking University, Beijing, China; 4 Institute for Healthy China, Tsinghua University, Beijing, China; 5 Vanke School of Public Health, Tsinghua University, Beijing, China; 6 School of Medicine, Stanford University, Stanford, California, USA; 7 Representative Office for China, World Health Organization, Beijing, China; 8 Institute for Global Health and Development, Peking University, Beijing, China; 9 Institute of Environmental Medicine, Peking University, Beijing, China

## Abstract

**Background:**

Over 30 priority pathogens with pandemic potential were identified, underscoring the need for targeted surveillance and prevention. As infectious disease outbreaks increase globally, particularly from zoonotic and vector-borne pathogens, understanding their distribution is crucial for effective public health responses. We aimed to provide a comprehensive analysis of global infectious disease outbreaks from 1996–2023, addressing gaps in previous research.

**Methods:**

We sourced data from the World Health Organization emergency events webpage, focusing on key details like disease name, location, date, and fatalities. We calculated case fatality rates (CFR) to assess outbreak severity. We categorised outbreaks into six types – respiratory, vector-borne, foodborne/waterborne, direct contact infections, non-infectious conditions, and others. Data extraction was independently performed and cross-verified for accuracy.

**Results:**

Between 1996–2023, a total of 3013 global outbreak events were reported. The Democratic Republic of the Congo had the highest frequency of outbreaks, with 272 events, followed by China with 254, and Saudi Arabia with 202. Influenza was the most frequently reported disease, with 771 outbreaks, followed by Ebola (n = 342) and Middle East respiratory syndrome-related coronavirus (MERS-CoV) (n = 305). Significant outbreaks included the 2023 global dengue outbreak, which accounted for five million cases and 5000 deaths. The CFR was highest for the Marburg virus at 76.86%, followed by haemorrhagic fever at 63.63%, and Ebola at 63.00%. The data underscore the varying severity and distribution of outbreaks, highlighting the critical need for robust global health surveillance and targeted interventions.

**Conclusions:**

In this study, we highlighted the significant impact of influenza, Ebola, and MERS-CoV. The high case fatality rates of viruses like Marburg and Ebola emphasised the need for early detection and rapid response systems. Strengthening global cooperation, investing in health care infrastructure, and integrating digital surveillance technologies are crucial to enhancing preparedness and reducing future outbreak impacts.

The World Health Organization (WHO) has identified over 30 priority pathogens that could cause global public health emergencies, including influenza A viruses, dengue virus, and monkeypox virus [1]. This updated list highlights the importance of focusing efforts on these high-risk pathogens due to their high transmissibility, virulence, and the limited availability of vaccines and treatments. As global challenges such as climate change, deforestation, and increased urbanisation increase the risk of these diseases spreading, it is crucial to prioritise surveillance and prevention strategies tailored to these pathogens. Understanding the spatio-temporal distribution of emerging infectious diseases is one of the most critical and challenging tasks of the next century [2]. There has been a global rise in the frequency of human infectious disease outbreaks. These outbreaks are caused by various pathogens, including bacteria and viruses, the majority of which either originate in animals or are transmitted via vectors, accounting for the bulk of outbreaks [3]. This trend might correlate with a series of severe infectious disease outbreaks that the 21st century has witnessed, including the most recent COVID-19 pandemic, which has had a devastating impact on lives and livelihoods globally [4]. Significant efforts have been made to establish surveillance and response systems for early detection of epidemics, to respond at a global scale, and to curb transmission at its source, especially after the severe acute respiratory syndrome (SARS) epidemic [5].

The burden of infectious diseases is still substantial in low- and middle-income countries, with persistently high morbidity and mortality associated with neglected tropical diseases, HIV infection, tuberculosis, and malaria [6–8]. However, there tends to be a delay in the global response to disease outbreaks, particularly between the source of an outbreak and taking collective action [9]. Improving surveillance capabilities and expediting decision response time are vital for minimising disease spread. The advent of information technology, especially the internet, provides a potential solution through the availability of online information sources. Early detection of global disease outbreaks is crucial, as it can initiate effective public health intervention measures and timely alert government agencies and the public [10]. This situation calls for leveraging the internet more effectively to enhance the transparency of disease reporting, reduce the costs of outbreak detection, and optimise reporting efficiency. However, past studies lack large-scale spatio-temporal data documenting distributions for many pathogens, and this has impeded disease biogeographers from fully characterising the global disease-scape.

A previous study described 70 infectious diseases and 2227 public health events in 233 countries and regions from 1996–2022, revealing disease outbreak hotspots in Africa, the Americas, and Asia through spatial analysis, aiding policymakers in understanding and responding to disease outbreaks [11]. However, the study recorded the occurrence of outbreaks, not their intensity, and lacked detailed subnational data. Therefore, the primary objective of this study was to provide a comprehensive analysis of global infectious disease outbreaks from 1996–2023, with a particular focus on the frequency, geographical distribution, and health impacts of these events.

By systematically documenting and analysing outbreak events across various regions, we aimed to fill the gaps left by previous research, particularly in terms of understanding the intensity of outbreaks. Additionally, we sought to identify patterns in the types of diseases most frequently responsible for outbreaks, as well as the variations in case fatality rates among different pathogens. The insights gained from this analysis will not only enhance our understanding of the global disease landscape but also inform future strategies for disease surveillance, preparedness, and response, particularly in regions with high vulnerability to infectious diseases. Ultimately, this study aims to contribute to the ongoing efforts to reduce the global burden of infectious diseases by providing a robust evidence base for targeted public health interventions and policy development.

## METHODS

### Study design and data sources

This secondary data analysis utilised an observational study design (retrospective analysis), wherein we systematically extracted the data from the WHO emergency events webpage [12]. This reliable source provides an extensive collection of data on public health emergencies across the globe, which are compiled and updated by the WHO. The webpage contains structured and comprehensive records of various types of emergencies, including epidemics, conflicts, and natural disasters. The emergencies are reported in an efficient and systematic manner, making this database a robust and valuable resource for public health analyses. Each reported emergency event includes information on the nature of the emergency, the country or region affected, the date when the event was reported, the health hazard posed by the event, and the WHO actions taken in response. The transparency and accessibility of the data from the WHO Emergency Events webpage establish its validity and provide an invaluable foundation for our study [12].

### Inclusion and exclusion criteria

We included all outbreak reports listed on WHO emergency events webpage as of December 2023, and the exclusion criteria were: 1) non-infectious disease outbreaks (*e.g.* acute flaccid paralysis and acute jaundice syndrome), 2) COVID-19 related events (excluded due to dedicated WHO COVID-19 reporting portals), and 3) animal-only outbreaks without human transmission.

### Data collection

We performed data extraction using Microsoft Excel (Microsoft, Redmond, Washington, USA) through manual review of all outbreak reports on the WHO emergency events webpage. The extraction workflow included: 1) primary data layer (mandatory fields extracted from all reports), disease name, emergency location (country/region), news publication date, and event reporting date; 2) secondary data layer (supplementary fields extracted when available), number of confirmed cases and death counts; 3) validation protocol: dual independent extraction by two researchers (QL and XL), 100% cross-verification for primary data fields, third researcher arbitration for discrepancies (WJ), uniform resource locator archiving for all source reports (Table S1 in the **Online Supplementary Document**).

It is noteworthy that the availability of this detailed set of data varied across reports, given that not all of these specific statistics were reported in each news item. However, all accessible relevant data were meticulously recorded to support a comprehensive understanding of the outbreak characteristics.

In order to facilitate a structured and informative analysis, we classified the outbreaks reported in the news items into five broad categories, based on the mode of transmission. This classification was essential as diseases with different transmission mechanisms require distinct prevention and control strategies. The categories were as follows: 1) respiratory infections, encompassing diseases primarily transmitted through respiratory droplets, 2) vector-borne infections, which are diseases transmitted by vectors such as mosquitoes, ticks, and fleas, 3) foodborne or waterborne outbreaks, highlighting those conditions resulting from consumption of contaminated food or water, 4) direct contact infections, encompassed diseases predominantly spread through direct physical contact with an infected person or animal, and 5) other outbreaks, capturing those conditions which could not be accurately classified into the first five categories, including diseases with multiple modes of transmission, the mode of transmission or nature of the pathogen was not clearly defined, or was unique (**Table 1**). This categorisation strategy aided in providing a more nuanced understanding of the outbreak patterns and their respective public health impacts.

**Table 1:** Classification of outbreaks

Category	Name of the outbreaks
Respiratory infections	Coccidioidomycosis, diphtheria, influenza, measles, MERS-CoV, nCoV, *Pertussis*, pneumonia, SARS, XDR-TB, other respiratory syndromes
Vector-borne infections	*Arenaviridae*, chikungunya, dengue, hantavirus, leishmaniasis, malaria, Mayaro fever, O'nyong'nyong fever, Oropouche virus, plague, relapsing fever, Rift Valley fever, Seoul virus, tularemia, West Nile virus, yellow fever, Zika
Foodborne or waterborne outbreaks	Botulism, cholera, diarrhoea, dracunculiasis, dysentery, *Escherichia coli*, enterovirus, food-borne intoxication, legionellosis, leptospirosis, listeriosis, poliovirus, salmonella/salmonella typhimurium, shigella, typhoid fever
Direct contact infections	Anthrax, buffalopox, Creutzfeldt-Jakob disease, Ebola, hand-foot-and-mouth disease, Lassa fever, Marburg virus, monkeypox, rabies, smallpox, *Staphylococcus aureus*, *Streptococcus*, surgical site infections caused by antibiotic-resistant *Pseudomonas aeruginosa*
Other outbreaks	Acute febrile illness, acute hepatitis, borreliosis, *Elizabethkingia anopheles*, encephalitis, haemorrhagic fever, hendra-like virus, HIV, Kwazulu-natal, meningitis, nipah virus, reston virus, XDR *Neisseria gonorrhoeae*, unknown

MERS-COV – Middle East respiratory syndrome-related coronavirus, nCoV – novel coronavirus, SARS – severe acute respiratory syndrome, TB – tuberculosis, XDR – extensively drug resistant

### Statistical analysis

We calculated the case fatality rate (CFR), a vital measure that provides insights into the severity of an outbreak, for those events where both the number of cases and the number of deaths were reported. The CFR signifies the proportion of reported cases that result in fatalities, thus allowing an understanding of the lethality of a particular disease or outbreak. It’s important to note that a higher CFR indicates a more lethal outbreak, while lower rates may suggest a higher survivability or a more effective control and management of that particular outbreak condition. Thus, a comprehensive understanding of the CFR not only contributes to the understanding of the severity of the disease but also directs the necessary public health response. For the calculation of the CFR we followed the formula:



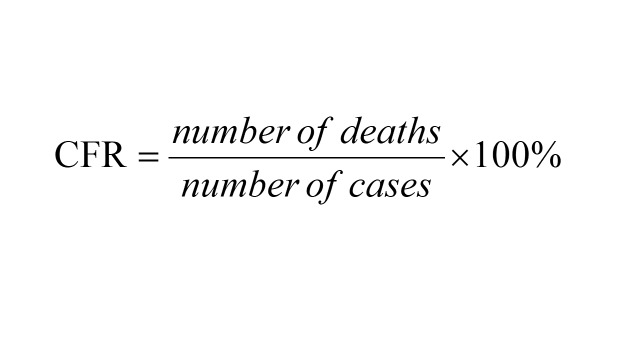



By this calculation, the CFR is expressed as a percentage, indicating the proportion of cases that resulted in death. For all statistical analyses, we used *R*, version 4.3.1 (R Core Team, Vienna, Austria).

Our primary outcomes, including disease type, location, and timing, had 100% completeness. For the secondary outcomes, the availability of case and death data was 2176 out of 3013 events, representing 72.2%. The CFR calculations were restricted to events that had both case and death reports, with a total of 1688 events included. No imputation was applied, as the CFR calculation relies on paired case and death data.

### Technical validation

We ensured the credibility of our data set through multiple checks and confirmations. First, the data we used were primarily obtained from the WHO Emergency Events webpage, attesting to its credibility and authenticity. However, to ensure its reliability for our study, a thorough validation process was performed. We cross-checked the data with other verifiable records and found high coherence, which confirmed the robustness of our primary data source. Second, the intense scrutiny that each news report underwent for data extraction added another validation layer. The dual-layered examination of the data by two independent researchers not only minimised potential bias but also elevated the consistency and reliability of the data, as discrepancies were resolved with the involvement of a third researcher. Next, the calculation of the CFR underwent a precise mathematical process for every event where both the number of cases and the number of deaths were documented. Moreover, the categorisation of outbreaks into five broad categories was based on the mode of transmission, type of pathogen, and nature of the outbreak, exemplifying a structured approach. This classification underwent a validation process, wherein we again ensured the congruity of our category assignments with those made by recognised public health organisations. Finally, reiterating the transparency of our process, we documented each step meticulously, providing concrete reference points for each data source. This ensured the traceability of our research process, allowing for independent verification if needed.

In conclusion, the validation protocol employed in our study ensured that our analysis and results are rooted in credible, reliable, and robust data. The rigorous scrutiny at each stage of data processing affirms the authenticity of our findings and their potential contribution to understanding public health emergencies.

## RESULTS

### Frequency of global infectious outbreak reports between 1996–2023

**Figure 1:**
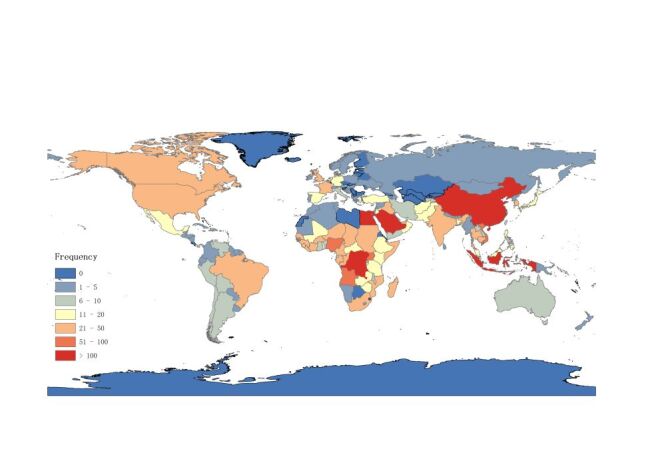
Geographic distribution of reported outbreak events globally between 1996–2023.

Between 1996–2023, a total of 3013 outbreak events were reported globally, including 1305 respiratory infections, 436 vector-borne infections, 469 foodborne or waterborne outbreaks, 484 direct contact infections, and 319 other outbreaks (**Figure 1**). The Democratic Republic of the Congo had the highest frequency of reported outbreak events, with a total of 272 instances. This was closely followed by China and Saudi Arabia, with a total of 254 and 202 reports, respectively. Two other countries, Indonesia and Egypt, had also reported more than 100 outbreak events during this period, registering 139 and 107 instances respectively. Additionally, there were 188 outbreak events involving multiple countries, yet the specific countries involved in these multi-country events were not reported, and thus, these events are not included in the individual total count for each country.

**Figure 2:**
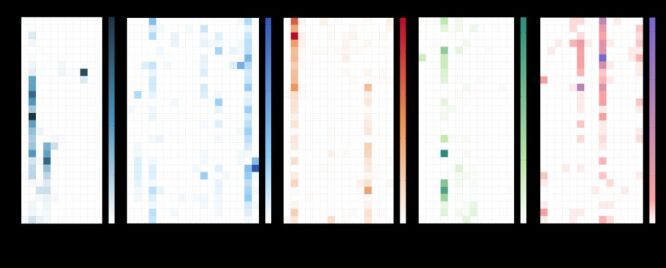
Global outbreak frequency across various infectious diseases between 1996–2023.

Considering the outbreaks of various diseases, influenza has been reported more frequently with a total of 771 outbreaks, followed by Ebola with 342 and Middle East respiratory syndrome-related coronavirus (MERS-CoV) with 305 cases (**Table 2**; Table S2 in the **Online Supplementary Document**). Within the category of respiratory infections, influenza was reported almost annually, with the apex of 127 reports in 2009, encompassing 82 outbreaks of H1N1 and 29 of H5N1. These pandemic instances spanned multiple countries, territories, and areas, with certain reports covering more than 200 countries, territories, or communities. Furthermore, MERS-CoV had been reported for 11 years, peaking in 2015 with a global count of 94, while SARS had been reported globally 112 times in 2003. Within vector-borne infections, yellow fever outbreaks were reported nearly every year, with marked surges in 2001 and 2016, registering 14 and 12 reports, respectively, the majority of which originated from African nations. Additionally, there were 31 global reports of Zika outbreaks in 2016. The foodborne or waterborne infections category witnessed a high frequency of Cholera and poliomyelitis reports, with outbreaks reported nearly every year. The frequency of Cholera outbreaks reached its zenith in 1998 with 48 global reports, followed by 33 in 1996, and 23 in 2005. Poliomyelitis saw 20 reported outbreaks in 2019. The predominance of Ebola outbreaks was evident within direct contact infections, with over 10 outbreak occurrences in nine different years, peaking conspicuously in 2014 with 65 global reports, followed by 53 reports in 2019, and 36 in 2018. In other outbreaks, meningitis and haemorrhagic fever were reported most commonly, with meningitis incidents peaking in 2001 at 43, and haemorrhagic fever reaching 24 reports in 2005 (**Figure 2**).

**Table 2:** Total number of reports of outbreaks, number of cases and deaths caused by the outbreaks (if reported), and case fatality rate of the outbreaks*

Outbreaks’ name	Reported	Cases	Deaths	CFR (%)
Respiratory infections				
*Influenza*	771	3 285 450	577 523	17.58
*MERS-CoV*	305	6418	1793	27.94
*SARS*	123	306 203	22 928	7.49
*Measles*	43	755 799	2110	0.28
*nCoV*	24	327	147	44.95
Vector-borne infections				
*Yellow fever*	164	779 323	3434	0.44
*Dengue*	73	12 977 361	12 327	0.09
*Zika*	47	317	0	0.00
*Rift Valley fever*	38	7315	1959	26.78
*Plague*	36	10 492	1004	9.57
Foodborne or waterborne infections				
*Cholera*	289	4 904 288	149 988	3.06
*Poliovirus*	112	5569	91	1.63
*Diarrhoea†*	13	246 619	504	0.20
*Enterovirus*	13	2056	260	12.65
*Legionellosis*	12	2189	63	2.88
Direct contact infections				
*Ebola*	342	226 701	142 813	63.00
*Lassa fever*	44	8934	1617	18.10
*Marburg virus*	28	795	611	76.86
*Monkeypox*	26	23 062	840	3.64
*Anthrax*	18	932	42	4.51
Other outbreaks				
*Meningitis*	178	609 772	60 299	9.89
*Haemorrhagic fever*	68	8095	5151	63.63
*Unknown*	23	16 678	1182	7.09
*Acute hepatitis*	18	16 952	338	1.99
*Encephalitis*	12	4250	763	17.95

CFR – case fatality rate, MERS-CoV – Middle East respiratory syndrome-related coronavirus, nCoV – novel coronavirus, SARS – severe acute respiratory syndrome

*Presented as n unless specified otherwise. The reported numbers of cases and deaths in this table represent cumulative counts aggregated from each outbreak report. For certain infectious diseases, multiple reports may document additional cases and deaths during the same outbreak, resulting in overlapping data being included in the totals. Consequently, the aggregated numbers of cases and deaths reflect all reported instances without de-duplication. This approach is appropriate for calculating the CFR since it is derived directly from the aggregated reported cases and deaths within the same data framework, ensuring consistency and comparability across outbreaks. Five outbreaks with the top reported numbers were shown in each category (Table S2 in the **Online Supplementary Document**).

†Depending on the exact cause.

The data thus shed light on both the periodic patterns and sporadic occurrences of an array of infectious diseases across geographic locales, underlining the need for relentless, proactive global health surveillance and strategic prevention and control of infectious diseases.

### The health impact of the outbreaks

In outbreaks where the number of cases or deaths were reported, the 2009 H1N1 influenza outbreak resulted in 414 000 cases and 1799 deaths worldwide. The impact of measles outbreaks should not be overlooked, as eight of the top ten outbreaks in terms of case numbers were due to measles. Dengue, cholera, and meningitis also have significant health impacts, dominating the top ten outbreaks in their respective categories. The worldwide dengue outbreak in 2023 accounted for the highest number of cases and deaths, at 5 000 000 and 5000, respectively. Furthermore, the top seven deadliest outbreaks of vector-borne diseases were all due to dengue. In 2023, cholera resulted in 2 900 000 cases and 95 000 deaths globally. Meningitis caused the greatest number of cases (n = 67 681) in Somalia in 2002 and the most deaths (n = 8955) in Africa in 1996. For outbreaks of direct contact infections, monkeypox resulted in the highest number of cases, while Ebola resulted in the highest number of deaths, with Democratic Republic of Congo being severely affected by both. (Table S3 in the **Online Supplementary Document**).

**Figure 3:**
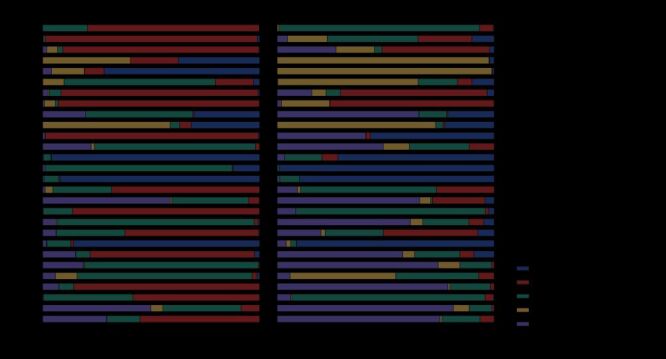
Percentage of cases and deaths of the outbreaks between 1996–2023, by six categories.

There was a remarkable variation among different years when examining the proportions of cases or deaths attributed to distinct types of outbreaks, considering all outbreaks each year. Predominantly, the outbreaks of vector-borne infections contributed to the majority of cases in most years, but they only led in mortality in certain years, notably 2016, 2017, and 2021. In particular years, one outbreak category overpowered, such as the instances of respiratory infection outbreak in years 2003, 2009, and 2011; vector-borne infection outbreaks in 2022; reported fatalities from respiratory infection outbreaks in 2003 and 2010; fatalities from direct contact infection outbreaks in 2019 and 2020; and fatalities from foodborne or waterborne outbreaks in 2023. (**Figure 3**)

When the total count of cases and deaths from all the outbreaks was consolidated and the case fatality rate was computed, the Marburg virus claimed the highest position with a substantial case fatality rate of 76.86% (n/N = 611/795). This was closely followed by haemorrhagic fever and Ebola, which recorded case fatality rates of 63.63% (n/N = 5151/8095) and 63.00% (n/N = 142 813/226 701), respectively. Remarkably, the Nipah virus had a case fatality rate exceeding 50%, precisely, 58.51% (n/N = 55/94). In contrast, the overall case fatality rates for categories such as foodborne or waterborne outbreaks, and vector-borne infections presented relatively lower figures. Specifically, the highest fatality rates in each of these categories were attributed to Listeriosis at 17.62% (n/N = 400/2270), and Rift Valley Fever at 26.78% (n/N = 1959/7315). (**Table 2**)

## DISCUSSION

To the best of our knowledge, this study represents the first comprehensive analysis of global infectious outbreaks between 1996–2023, providing crucial insights into their frequency, geographical distribution, and health impacts. Our findings reveal that over 3000 outbreak events were reported globally during this period, with the Democratic Republic of the Congo, China, and Saudi Arabia being the most affected countries. Notably, influenza, Ebola, and MERS-CoV were the most frequently reported diseases, with influenza alone accounting for 771 outbreaks. The health impacts were substantial, with the 2009 H1N1 influenza outbreak caused 414 000 cases and nearly 1800 deaths worldwide. Vector-borne diseases, particularly dengue and cholera, demonstrated significant morbidity and mortality, with the 2023 dengue outbreak leading to five million cases and 5000 deaths globally. Additionally, the Marburg virus, haemorrhagic fevers, and Ebola exhibited alarmingly high case fatality rates, with Marburg reaching nearly 77%. The study also sheds light on the significant variation in the impact of different types of outbreaks over the years, with certain years dominated by specific categories such as respiratory infections or vector-borne diseases. The findings of this study underscore the persistent and evolving threat of infectious disease outbreaks, emphasising the need for continuous global health surveillance, strategic preparedness, and targeted interventions. By mapping the patterns and health impacts of outbreaks over nearly three decades, this study highlights the critical need for proactive and coordinated efforts to mitigate the risks posed by infectious diseases, particularly in regions with high vulnerability. Overall, this study contributes valuable knowledge to the field of global health and serves as a foundational resource for guiding policy and public health strategies aimed at preventing and controlling infectious disease outbreaks worldwide.

Our findings showed that between 1996–2023, 3013 global infectious disease outbreaks were reported, with the Democratic Republic of Congo leading in frequency, followed by China and Saudi Arabia. The recurrence of outbreaks in these countries can be attributed to a combination of factors, including high population density, variations in health care infrastructure, and environmental conditions. For example, endemic diseases such as Ebola and malaria, prevalent in the Democratic Republic of Congo, contribute significantly to the frequency of outbreaks. Furthermore, mass gatherings, such as religious festivals in Saudi Arabia and China, can facilitate the spread of infectious diseases, highlighting the importance of addressing public health risks in such settings [13–15]. Effective public health surveillance systems are critical in controlling infectious disease outbreaks, as they provide continuous and real-time data for early detection and rapid response. The integration of digital technologies has the potential to enhance the timeliness and accuracy of disease reporting, improving response strategies. However, despite advancements, the role of digital surveillance in preventing outbreaks during large gatherings remains insufficiently evaluated, leaving a gap in the ability to optimise these technologies for public health interventions. The lack of robust assessment frameworks for digital surveillance underscores the need for further research and innovation in this area [16,17]. Given these challenges, it is recommended that countries with high outbreak frequencies, such as the Democratic Republic of Congo, China, and Saudi Arabia, invest strategically in strengthening their public health surveillance systems, focusing on integrating digital technologies and ensuring that these systems can adequately respond to the unique risks posed by mass gatherings. Furthermore, public health policies should prioritise enhancing health care infrastructure in high-risk regions and developing targeted strategies that address the specific challenges posed by recurrent outbreaks and large-scale events. By improving these areas, countries can better prepare for and mitigate the impact of future outbreaks.

Influenza was the most frequently reported disease, with a total of 771 outbreak events, followed by Ebola virus and MERS-CoV. Other frequently reported diseases included yellow fever, cholera, and meningitis. The high frequency of influenza outbreaks highlights its substantial global burden and the persistent challenges it poses to public health systems, especially in regions like Asia, where both seasonal and avian influenza remain major concerns [18]. Studies have shown that countries such as China have experienced significant mortality from upper respiratory infections, with emerging strains such as H5N1 and H7N9 further complicating disease control efforts [19,20]. The emergence of highly transmissible and virulent outbreaks like Ebola and MERS-CoV underscores the urgent need for robust health care policies, comprehensive preparedness plans, and enhanced global surveillance systems to mitigate their spread. [21] Meanwhile, the frequent reports of yellow fever, cholera, and meningitis highlight the ongoing threat of vector-borne and waterborne diseases, particularly in resource-limited settings. Environmental factors, such as increased heavy rainfall and flooding, often lead to water contamination, triggering outbreaks, especially in developing regions [22,23]. Additionally, rising temperatures and deteriorating water quality further heighten the risks associated with these diseases, underscoring the need for proactive public health measures [24,25]. Addressing these ongoing and emerging threats requires coordinated international efforts focused on strengthening disease surveillance, improving health care infrastructure in high-risk regions, and promoting timely access to vaccines and treatments. Furthermore, improving environmental management practices and establishing sustainable water and sanitation systems are critical steps in mitigating the impact of vector-borne and waterborne diseases, ultimately contributing to more resilient public health systems worldwide.

While high-frequency infectious disease outbreaks demand significant attention, it is equally important to recognise the profound health losses caused by single, severe outbreaks. The 2003 SARS outbreak underscores the devastating impact of respiratory infections on global health, highlighting the urgent need for robust international response systems and preparedness for future pandemics. The 2023 dengue fever outbreak, resulting in five million cases and 5000 deaths, emphasises the growing threat posed by vector-borne diseases, particularly in regions with inadequate vector control measures and health care infrastructure. Historical outbreaks such as the 1998 cholera epidemic, which caused 2.9 million cases and 95 000 deaths, and the 2002 meningitis outbreak in Somalia further illustrate the vulnerability of populations to waterborne diseases and meningitis in resource-limited settings. The alarmingly high case fatality rates of the Marburg virus (76.86%), Ebola (63.00%), and haemorrhagic fever (63.63%) highlight the critical need for early detection, rapid response, and effective treatment strategies to reduce mortality in outbreaks of highly virulent pathogens. Collectively, these outbreaks highlight the diverse and significant challenges posed by infectious diseases, underscoring the necessity of global collaboration, strengthened health care systems, and targeted public health interventions. Given the historical severity of these outbreaks, aligning surveillance and prevention efforts with WHO’s list of priority pathogens, encompassing over 30 high-risk agents, is essential. This list, which now includes influenza A viruses, dengue virus, monkeypox virus, and entire subgenera of coronaviruses, underscores the urgency of vigilant monitoring and targeted interventions. As global factors such as climate change, deforestation, and urbanisation heighten the risk of these disease emergence and transmission, proactive public health measures will be critical in mitigating future outbreaks and safeguarding global health [1].

In this study, several limitations should be acknowledged. First, our analysis of infectious disease outbreaks was confined to the national level, which did not account for the sub-national distribution of outbreaks. This limitation might obscure significant regional variations in the spread and impact of infectious diseases within countries, potentially leading to an incomplete understanding of the true geographic distribution of outbreaks. Second, not all outbreak reports included comprehensive data on infection rates, mortality, or other critical indicators of disease severity. Consequently, our comparisons of outbreak severity were based only on those events for which such data were available. It is important to note that the WHO Emergency Events webpage is an essential tool for monitoring global outbreaks, but its data may be influenced by several factors, including regional disparities in health care infrastructure, differences in surveillance capacities, and reporting practices. Some regions may have more comprehensive reporting mechanisms, while others may underreport due to limited resources or differing priorities. These factors could introduce potential biases, affecting the completeness and accuracy of the data. Third, while our transmission-based classification optimally informs containment strategies, it does not explicitly distinguish pathogen types (viral, bacterial, *etc.*), which may influence therapeutic development. Future studies integrating both transmission pathways and pathogen phylogeny could further refine risk-stratification frameworks. Despite these limitations, our study remained valuable in providing a comprehensive overview of the global distribution and impact of infectious disease outbreaks, highlighting key patterns and trends that could inform public health strategies and preparedness efforts.

## CONCLUSIONS

In conclusion, this study represented the first comprehensive analysis of global infectious disease outbreaks between 1996–2023, providing crucial insights into their frequency, geographical distribution, and health impacts. With over 3000 outbreak events reported during this period, the findings highlighted the significant burden of diseases such as influenza, Ebola, and MERS-CoV, which caused substantial morbidity and mortality worldwide. The study also emphasised the growing threat of vector-borne and waterborne diseases, such as the 2023 dengue fever outbreak and the 1998 cholera epidemic, particularly in regions with inadequate health care infrastructure and vector control measures. The high case fatality rates of viruses like Marburg and Ebola emphasised the need for early detection and rapid response systems. Despite the study’s limitations, it underscored the importance of continuous global surveillance and targeted public health interventions. To enhance global preparedness, policies should focus on strengthening international cooperation, investing in health care infrastructure, and ensuring timely access to vaccines and treatments in vulnerable regions. Additionally, integrating advanced digital surveillance technologies could improve early detection and response strategies, ultimately reducing the impact of future outbreaks.

## Additional material


Online Supplementary Document

## References

[1] World Health Organization. Pathogens prioritization: a scientific framework for epidemic and pandemic research preparedness. 2024. Available: https://www.who.int/publications/m/item/pathogens-prioritization-a-scientific-framework-for-epidemic-and-pandemic-research-preparedness. Accessed: 28 August 2024.

[2] Bonds MH Dobson AP Keenan DC Disease ecology, biodiversity, and the latitudinal gradient in income. PLoS Biol. 2012;10:e1001456. 10.1371/journal.pbio.1001456 23300379 PMC3531233

[3] Smith KF Goldberg M Rosenthal S Carlson L Chen J Chen C Global rise in human infectious disease outbreaks. J R Soc Interface. 2014;11:20140950. 10.1098/rsif.2014.0950 25401184 PMC4223919

[4] Baker RE Mahmud AS Miller IF Rajeev M Rasambainarivo F Rice BL Infectious disease in an era of global change. Nat Rev Microbiol. 2022;20:193–205. 10.1038/s41579-021-00639-z 34646006 PMC8513385

[5] Hoffman SJ The evolution, etiology and eventualities of the global health security regime. Health Policy Plan. 2010;25:510–22. 10.1093/heapol/czq037 20732860

[6] Liu Q Yan W Qin C Du M Wang Y Liu M Incidence and mortality trends of neglected tropical diseases and malaria in China and ASEAN countries from 1990 to 2019 and its association with the socio-demographic index. Glob Health Res Policy. 2023;8:22. 10.1186/s41256-023-00306-1 37349771 PMC10288805

[7] Liu Q Jing W Kang L Liu J Liu M Trends of the global, regional and national incidence of malaria in 204 countries from 1990 to 2019 and implications for malaria prevention. J Travel Med. 2021;28:taab046. 10.1093/jtm/taab046 33763689 PMC8271200

[8] Du M Yan W Jing W Qin C Liu Q Liu M Increasing incidence rates of sexually transmitted infections from 2010 to 2019: an analysis of temporal trends by geographical regions and age groups from the 2019 Global Burden of Disease Study. BMC Infect Dis. 2022;22:574. 10.1186/s12879-022-07544-7 35754034 PMC9233762

[9] Hoffman SJ Silverberg SL Delays in Global Disease Outbreak Responses: Lessons from H1N1, Ebola, and Zika. Am J Public Health. 2018;108:329–33. 10.2105/AJPH.2017.304245 29345996 PMC5803810

[10] Wilson K Brownstein JS Early detection of disease outbreaks using the Internet. CMAJ. 2009;180:829–31. 10.1503/cmaj.1090215 19364791 PMC2665960

[11] Torres Munguía JA Badarau FC Díaz Pavez LR Martínez-Zarzoso I Wacker KM A global dataset of pandemic- and epidemic-prone disease outbreaks. Sci Data. 2022;9:683. 10.1038/s41597-022-01797-2 36357405 PMC9648436

[12] World Health Organization. WHO emergency events. 2024. Available: https://www.who.int/emergencies/emergency-events. Accessed: 28 August 2024.

[13] Burkle FM Declining Public Health Protections within Autocratic Regimes: Impact on Global Public Health Security, Infectious Disease Outbreaks, Epidemics, and Pandemics. Prehosp Disaster Med. 2020;35:237–46. 10.1017/S1049023X20000424 32238221 PMC7156578

[14] Memish ZA Zumla A Alhakeem RF Assiri A Turkestani A Al Harby KD Hajj: infectious disease surveillance and control. Lancet. 2014;383:2073–82. 10.1016/S0140-6736(14)60381-0 24857703 PMC7137990

[15] Carratalà A Joost S Population density and water balance influence the global occurrence of hepatitis E epidemics. Sci Rep. 2019;9:10042. 10.1038/s41598-019-46475-3 31296895 PMC6624372

[16] Maddah N Verma A Almashmoum M Ainsworth J Effectiveness of Public Health Digital Surveillance Systems for Infectious Disease Prevention and Control at Mass Gatherings: Systematic Review. J Med Internet Res. 2023;25:e44649. 10.2196/44649 37204833 PMC10238952

[17] Jia P Liu S Yang S Innovations in Public Health Surveillance for Emerging Infections. Annu Rev Public Health. 2023;44:55–74. 10.1146/annurev-publhealth-051920-093141 36626834

[18] Liu Q Qin C Du M Wang Y Yan W Liu M Incidence and Mortality Trends of Upper Respiratory Infections in China and Other Asian Countries from 1990 to 2019. Viruses. 2022;14:2550. 10.3390/v14112550 36423159 PMC9697955

[19] Tang RB Chen HL An overview of the recent outbreaks of the avian-origin influenza A (H7N9) virus in the human. J Chin Med Assoc. 2013;76:245–8. 10.1016/j.jcma.2013.04.003 23651506

[20] Neumann G Chen H Gao GF Shu Y Kawaoka Y H5N1 influenza viruses: outbreaks and biological properties. Cell Res. 2010;20:51–61. 10.1038/cr.2009.124 19884910 PMC2981148

[21] Weber DJ Rutala WA Fischer WA Kanamori H Sickbert-Bennett EE Emerging infectious diseases: Focus on infection control issues for novel coronaviruses (Severe Acute Respiratory Syndrome-CoV and Middle East Respiratory Syndrome-CoV), hemorrhagic fever viruses (Lassa and Ebola), and highly pathogenic avian influenza viruses, A(H5N1) and A(H7N9). Am J Infect Control. 2016;44:e91–100. 10.1016/j.ajic.2015.11.018 27131142 PMC7132650

[22] Liu Q Yuan J Yan W Liang W Liu M Liu J Association of natural flood disasters with infectious diseases in 168 countries and territories from 1990 to 2019: A worldwide observational study. Glob Transit. 2023;5:149–59. 10.1016/j.glt.2023.09.001

[23] Liu Q Du M Wang Y Deng J Yan W Qin C Global, regional and national trends and impacts of natural floods, 1990-2022. Bull World Health Organ. 2024;102:410–20. 10.2471/BLT.23.290243 38812801 PMC11132161

[24] Hunter PR Climate change and waterborne and vector-borne disease. J Appl Microbiol. 2003;94:37S–46S. 10.1046/j.1365-2672.94.s1.5.x 12675935

[25] Liu Q Wang Y Deng J Yan W Qin C Du M Association of temperature and precipitation with malaria incidence in 57 countries and territories from 2000 to 2019: A worldwide observational study. J Glob Health. 2024;14:04021. 10.7189/jogh.14.04021 38385445 PMC10882640

