# Supplementary material for: Global distribution and health impact of infectious disease outbreaks, 1996–2023: a worldwide retrospective analysis of World Health Organization emergency event reports
Source: J Glob Health. 2025 May 16;15:04151. doi: 10.7189/jogh.15.04151 (PMC12082254; doi:10.7189/jogh.15.04151)
Supplement: Online Supplementary Document [file jogh-15-04151-s001.pdf]

Supplement to: Qiao L, Min L, Wannian L, Xuanjun L, Wenzhan J, Zhongdan C, Jue L. Global distribution and health impact of infectious disease outbreaks, 1996–2023: a worldwide retrospective analysis of WHO emergency event reports. J Glob Health. 2025;15:04151.

Table S1. Uniform Resource Locator archiving for all source reports.

Serial Number	News Date	Link
1	1996-01-22	https://www.who.int/emergencies/disease-outbreak-news/item/1996_01_22b-en
2	1996-01-22	https://www.who.int/emergencies/disease-outbreak-news/item/1996_01_22c-en
3	1996-01-22	https://www.who.int/emergencies/disease-outbreak-news/item/1996_01_22e-en
4	1996-01-22	https://www.who.int/emergencies/disease-outbreak-news/item/1996_01_22f-en
5	1996-01-22	https://www.who.int/emergencies/disease-outbreak-news/item/1996_01_22d-en
6	1996-01-22	https://www.who.int/emergencies/disease-outbreak-news/item/1996_01_22a-en
7	1996-02-02	https://www.who.int/emergencies/disease-outbreak-news/item/1996_02_02-en
8	1996-02-05	https://www.who.int/emergencies/disease-outbreak-news/item/1996_02_05-en
9	1996-02-08	https://www.who.int/emergencies/disease-outbreak-news/item/1996_02_08-en
10	1996-02-15	https://www.who.int/emergencies/disease-outbreak-news/item/1996_02_15-en
11	1996-02-19	https://www.who.int/emergencies/disease-outbreak-news/item/1996_02_19a-en
12	1996-02-19	https://www.who.int/emergencies/disease-outbreak-news/item/1996_02_19b-en
13	1996-02-19	https://www.who.int/emergencies/disease-outbreak-news/item/1996_02_19c-en
14	1996-02-29	https://www.who.int/emergencies/disease-outbreak-news/item/1996_02_29a-en
15	1996-02-29	https://www.who.int/emergencies/disease-outbreak-news/item/1996_02_29b-en
16	1996-03-01	https://www.who.int/emergencies/disease-outbreak-news/item/1996_03_01a-en
17	1996-03-01	https://www.who.int/emergencies/disease-outbreak-news/item/1996_03_01b-en
18	1996-03-01	https://www.who.int/emergencies/disease-outbreak-news/item/1996_03_01c-en
19	1996-03-06	https://www.who.int/emergencies/disease-outbreak-news/item/1996_03_06a-en
20	1996-03-06	https://www.who.int/emergencies/disease-outbreak-news/item/1996_03_06b-en
21	1996-03-06	https://www.who.int/emergencies/disease-outbreak-news/item/1996_03_06c-en
22	1996-03-07	https://www.who.int/emergencies/disease-outbreak-news/item/1996_03_07a-en
23	1996-03-07	https://www.who.int/emergencies/disease-outbreak-news/item/1996_03_07b-en
24	1996-03-12	https://www.who.int/emergencies/disease-outbreak-news/item/1996_03_12-en
25	1996-03-12	https://www.who.int/emergencies/disease-outbreak-news/item/1996_03_12b-en
26	1996-03-15	https://www.who.int/emergencies/disease-outbreak-news/item/1996_03_15a-en
27	1996-03-15	https://www.who.int/emergencies/disease-outbreak-news/item/1996_03_15b-en
28	1996-03-15	https://www.who.int/emergencies/disease-outbreak-news/item/1996_03_15c-en
29	1996-03-19	https://www.who.int/emergencies/disease-outbreak-news/item/1996_03_19-en
30	1996-03-21	https://www.who.int/emergencies/disease-outbreak-news/item/1996_03_21-en
31	1996-03-27	https://www.who.int/emergencies/disease-outbreak-news/item/1996_03_27-en

32	1996-03-28	https://www.who.int/emergencies/disease-outbreak-news/item/1996_03_28-en
33	1996-04-04	https://www.who.int/emergencies/disease-outbreak-news/item/1996_04_04b-en
34	1996-04-04	https://www.who.int/emergencies/disease-outbreak-news/item/1996_04_04a-en
35	1996-04-11	https://www.who.int/emergencies/disease-outbreak-news/item/1996_04_11-en
36	1996-04-17	https://www.who.int/emergencies/disease-outbreak-news/item/1996_04_17-en
37	1996-04-26	https://www.who.int/emergencies/disease-outbreak-news/item/1996_04_26-en
38	1996-04-26	https://www.who.int/emergencies/disease-outbreak-news/item/1996_04_26b-en
39	1996-04-30	https://www.who.int/emergencies/disease-outbreak-news/item/1996_04_30-en
40	1996-05-03	https://www.who.int/emergencies/disease-outbreak-news/item/1996_05_03-en
41	1996-05-15	https://www.who.int/emergencies/disease-outbreak-news/item/1996_05_15-en
42	1996-05-20	https://www.who.int/emergencies/disease-outbreak-news/item/1996_05_20-en
43	1996-05-28	https://www.who.int/emergencies/disease-outbreak-news/item/1996_05_28-en
44	1996-05-31	https://www.who.int/emergencies/disease-outbreak-news/item/1996_05_31-en
45	1996-06-03	https://www.who.int/emergencies/disease-outbreak-news/item/1996_06_03-en
46	1996-06-14	https://www.who.int/emergencies/disease-outbreak-news/item/1996_06_14a-en
47	1996-06-14	https://www.who.int/emergencies/disease-outbreak-news/item/1996_06_14c-en
48	1996-06-14	https://www.who.int/emergencies/disease-outbreak-news/item/1996_06_14d-en
49	1996-06-14	https://www.who.int/emergencies/disease-outbreak-news/item/1996_06_14b-en
50	1996-07-05	https://www.who.int/emergencies/disease-outbreak-news/item/1996_07_05-en
51	1996-07-05	https://www.who.int/emergencies/disease-outbreak-news/item/1996_07_05b-en
52	1996-07-11	https://www.who.int/emergencies/disease-outbreak-news/item/1996_07_11-en
53	1996-07-18	https://www.who.int/emergencies/disease-outbreak-news/item/1996_07_18-en
54	1996-07-19	https://www.who.int/emergencies/disease-outbreak-news/item/1996_07_19a-en
55	1996-07-19	https://www.who.int/emergencies/disease-outbreak-news/item/1996_07_19c-en
56	1996-07-19	https://www.who.int/emergencies/disease-outbreak-news/item/1996_07_19b-en
57	1996-07-26	https://www.who.int/emergencies/disease-outbreak-news/item/1996_07_26-en
58	1996-07-30	https://www.who.int/emergencies/disease-outbreak-news/item/1996_07_30a-en
59	1996-07-30	https://www.who.int/emergencies/disease-outbreak-news/item/1996_07_30b-en
60	1996-08-02	https://www.who.int/emergencies/disease-outbreak-news/item/1996_08_02-en
61	1996-08-06	https://www.who.int/emergencies/disease-outbreak-news/item/1996_08_06a-en
62	1996-08-06	https://www.who.int/emergencies/disease-outbreak-news/item/1996_08_06b-en
63	1996-08-12	https://www.who.int/emergencies/disease-outbreak-news/item/1996_08_12a-en
64	1996-08-12	https://www.who.int/emergencies/disease-outbreak-news/item/1996_08_12b-en
65	1996-08-12	https://www.who.int/emergencies/disease-outbreak-news/item/1996_08_12c-en
66	1996-08-13	https://www.who.int/emergencies/disease-outbreak-news/item/1996_08_13a-en
67	1996-08-13	https://www.who.int/emergencies/disease-outbreak-news/item/1996_08_13b-en
68	1996-08-14	https://www.who.int/emergencies/disease-outbreak-news/item/1996_08_14a-en
69	1996-08-14	https://www.who.int/emergencies/disease-outbreak-news/item/1996_08_14b-en
70	1996-08-14	https://www.who.int/emergencies/disease-outbreak-news/item/1996_08_14c-en

71	1996-08-21	https://www.who.int/emergencies/disease-outbreak-news/item/1996_08_21a-en
72	1996-08-21	https://www.who.int/emergencies/disease-outbreak-news/item/1996_08_21b-en
73	1996-08-28	https://www.who.int/emergencies/disease-outbreak-news/item/1996_08_28-en
74	1996-09-02	https://www.who.int/emergencies/disease-outbreak-news/item/1996_09_02a-en
75	1996-09-02	https://www.who.int/emergencies/disease-outbreak-news/item/1996_09_02b-en
76	1996-09-03	https://www.who.int/emergencies/disease-outbreak-news/item/1996_09_03-en
77	1996-09-09	https://www.who.int/emergencies/disease-outbreak-news/item/1996_09_09-en
78	1996-09-12	https://www.who.int/emergencies/disease-outbreak-news/item/1996_09_12-en
79	1996-09-18	https://www.who.int/emergencies/disease-outbreak-news/item/1996_09_18-en
80	1996-09-24	https://www.who.int/emergencies/disease-outbreak-news/item/1996_09_24-en
81	1996-10-02	https://www.who.int/emergencies/disease-outbreak-news/item/1996_10_02-en
82	1996-10-04	https://www.who.int/emergencies/disease-outbreak-news/item/1996_10_04-en
83	1996-10-14	https://www.who.int/emergencies/disease-outbreak-news/item/1996_10_14-en
84	1996-10-15	https://www.who.int/emergencies/disease-outbreak-news/item/1996_10_15a-en
85	1996-10-15	https://www.who.int/emergencies/disease-outbreak-news/item/1996_10_15b-en
86	1996-10-17	https://www.who.int/emergencies/disease-outbreak-news/item/1996_10_17a-en
87	1996-10-17	https://www.who.int/emergencies/disease-outbreak-news/item/1996_10_17b-en
88	1996-10-18	https://www.who.int/emergencies/disease-outbreak-news/item/1996_10_18-en
89	1996-10-21	https://www.who.int/emergencies/disease-outbreak-news/item/1996_10_21-en
90	1996-10-24	https://www.who.int/emergencies/disease-outbreak-news/item/1996_10_24-en
91	1996-10-31	https://www.who.int/emergencies/disease-outbreak-news/item/1996_10_31a-en
92	1996-10-31	https://www.who.int/emergencies/disease-outbreak-news/item/1996_10_31b-en
93	1996-11-01	https://www.who.int/emergencies/disease-outbreak-news/item/1996_11_01-en
94	1996-11-05	https://www.who.int/emergencies/disease-outbreak-news/item/1996_11_05-en
95	1996-11-12	https://www.who.int/emergencies/disease-outbreak-news/item/1996_11_12a-en
96	1996-11-12	https://www.who.int/emergencies/disease-outbreak-news/item/1996_11_12b-en
97	1996-11-15	https://www.who.int/emergencies/disease-outbreak-news/item/1996_11_15a-en
98	1996-11-15	https://www.who.int/emergencies/disease-outbreak-news/item/1996_11_15b-en
99	1996-11-18	https://www.who.int/emergencies/disease-outbreak-news/item/1996_11_18-en
100	1996-11-19	https://www.who.int/emergencies/disease-outbreak-news/item/1996_11_19-en
101	1996-11-20	https://www.who.int/emergencies/disease-outbreak-news/item/1996_11_20-en
102	1996-11-22	https://www.who.int/emergencies/disease-outbreak-news/item/1996_11_22-en
103	1996-11-25	https://www.who.int/emergencies/disease-outbreak-news/item/1996_11_25-en
104	1996-11-28	https://www.who.int/emergencies/disease-outbreak-news/item/1996_11_28a-en
105	1996-11-28	https://www.who.int/emergencies/disease-outbreak-news/item/1996_11_28b-en
106	1996-11-28	https://www.who.int/emergencies/disease-outbreak-news/item/1996_11_28c-en
107	1996-12-03	https://www.who.int/emergencies/disease-outbreak-news/item/1996_12_03-en
108	1996-12-05	https://www.who.int/emergencies/disease-outbreak-news/item/1996_12_05-en
109	1996-12-06	https://www.who.int/emergencies/disease-outbreak-news/item/1996_12_06a-en

110	1996-12-06	https://www.who.int/emergencies/disease-outbreak-news/item/1996_12_06b-en
111	1996-12-06	https://www.who.int/emergencies/disease-outbreak-news/item/1996_12_06c-en
112	1996-12-13	https://www.who.int/emergencies/disease-outbreak-news/item/1996_12_13a-en
113	1996-12-13	https://www.who.int/emergencies/disease-outbreak-news/item/1996_12_13b-en
114	1996-12-20	https://www.who.int/emergencies/disease-outbreak-news/item/1996_12_20-en
115	1996-12-24	https://www.who.int/emergencies/disease-outbreak-news/item/1996_12_24-en
116	1997-01-02	https://www.who.int/emergencies/disease-outbreak-news/item/1997_01_02-en
117	1997-01-08	https://www.who.int/emergencies/disease-outbreak-news/item/1997_01_08-en
118	1997-01-08	https://www.who.int/emergencies/disease-outbreak-news/item/1997_01_09b-en
119	1997-01-09	https://www.who.int/emergencies/disease-outbreak-news/item/1997_01_09a-en
120	1997-01-16	https://www.who.int/emergencies/disease-outbreak-news/item/1997_01_16-en
121	1997-01-17	https://www.who.int/emergencies/disease-outbreak-news/item/1997_01_17a-en
122	1997-01-17	https://www.who.int/emergencies/disease-outbreak-news/item/1997_01_17b-en
123	1997-01-20	https://www.who.int/emergencies/disease-outbreak-news/item/1997_01_20-en
124	1997-01-21	https://www.who.int/emergencies/disease-outbreak-news/item/1997_01_21-en
125	1997-01-22	https://www.who.int/emergencies/disease-outbreak-news/item/1997_01_22-en
126	1997-01-24	https://www.who.int/emergencies/disease-outbreak-news/item/1997_01_24-en
127	1997-01-27	https://www.who.int/emergencies/disease-outbreak-news/item/1997_01_27-en
128	1997-01-31	https://www.who.int/emergencies/disease-outbreak-news/item/1997_01_31a-en
129	1997-01-31	https://www.who.int/emergencies/disease-outbreak-news/item/1997_01_31b-en
130	1997-02-07	https://www.who.int/emergencies/disease-outbreak-news/item/1997_02_07a-en
131	1997-02-07	https://www.who.int/emergencies/disease-outbreak-news/item/1997_02_07b-en
132	1997-02-07	https://www.who.int/emergencies/disease-outbreak-news/item/1997_02_07c-en
133	1997-02-20	https://www.who.int/emergencies/disease-outbreak-news/item/1997_02_20-en
134	1997-02-28	https://www.who.int/emergencies/disease-outbreak-news/item/1997_02_28a-en
135	1997-02-28	https://www.who.int/emergencies/disease-outbreak-news/item/1997_02_28c-en
136	1997-02-28	https://www.who.int/emergencies/disease-outbreak-news/item/1997_02_28b-en
137	1997-03-02	https://www.who.int/emergencies/disease-outbreak-news/item/1997_03_02-en
138	1997-03-06	https://www.who.int/emergencies/disease-outbreak-news/item/1997_03_06-en
139	1997-03-11	https://www.who.int/emergencies/disease-outbreak-news/item/1997_03_11-en
140	1997-03-12	https://www.who.int/emergencies/disease-outbreak-news/item/1997_03_12-en
141	1997-03-21	https://www.who.int/emergencies/disease-outbreak-news/item/1997_03_21a-en
142	1997-03-21	https://www.who.int/emergencies/disease-outbreak-news/item/1997_03_21b-en
143	1997-03-21	https://www.who.int/emergencies/disease-outbreak-news/item/1997_03_21c-en
144	1997-03-27	https://www.who.int/emergencies/disease-outbreak-news/item/1997_03_27-en
145	1997-03-28	https://www.who.int/emergencies/disease-outbreak-news/item/1997_03_28-en
146	1997-04-11	https://www.who.int/emergencies/disease-outbreak-news/item/1997_04_11-en
147	1997-04-14	https://www.who.int/emergencies/disease-outbreak-news/item/1997_04_14-en
148	1997-04-15	https://www.who.int/emergencies/disease-outbreak-news/item/1997_04_15a-en

149	1997-04-15	https://www.who.int/emergencies/disease-outbreak-news/item/1997_04_15b-en
150	1997-04-24	https://www.who.int/emergencies/disease-outbreak-news/item/1997_04_24a-en
151	1997-04-24	https://www.who.int/emergencies/disease-outbreak-news/item/1997_04_24b-en
152	1997-05-01	https://www.who.int/emergencies/disease-outbreak-news/item/1997_05_01-en
153	1997-05-06	https://www.who.int/emergencies/disease-outbreak-news/item/1997_05_06a-en
154	1997-05-06	https://www.who.int/emergencies/disease-outbreak-news/item/1997_05_06b-en
155	1997-05-06	https://www.who.int/emergencies/disease-outbreak-news/item/1997_05_06c-en
156	1997-05-09	https://www.who.int/emergencies/disease-outbreak-news/item/1997_05_09-en
157	1997-05-20	https://www.who.int/emergencies/disease-outbreak-news/item/1997_05_20-en
158	1997-05-22	https://www.who.int/emergencies/disease-outbreak-news/item/1997_05_22-en
159	1997-06-04	https://www.who.int/emergencies/disease-outbreak-news/item/1997_06_04-en
160	1997-06-05	https://www.who.int/emergencies/disease-outbreak-news/item/1997_06_05-en
161	1997-06-06	https://www.who.int/emergencies/disease-outbreak-news/item/1997_06_06-en
162	1997-06-10	https://www.who.int/emergencies/disease-outbreak-news/item/1997_06_10-en
163	1997-06-18	https://www.who.int/emergencies/disease-outbreak-news/item/1997_06_18-en
164	1997-06-19	https://www.who.int/emergencies/disease-outbreak-news/item/1997_06_19a-en
165	1997-06-19	https://www.who.int/emergencies/disease-outbreak-news/item/1997_06_19b-en
166	1997-06-26	https://www.who.int/emergencies/disease-outbreak-news/item/1997_06_26-en
167	1997-07-03	https://www.who.int/emergencies/disease-outbreak-news/item/1997_07_03-en
168	1997-07-07	https://www.who.int/emergencies/disease-outbreak-news/item/1997_07_07-en
169	1997-07-30	https://www.who.int/emergencies/disease-outbreak-news/item/1997_07_30-en
170	1997-07-31	https://www.who.int/emergencies/disease-outbreak-news/item/1997_07_31a-en
171	1997-07-31	https://www.who.int/emergencies/disease-outbreak-news/item/1997_07_31b-en
172	1997-08-08	https://www.who.int/emergencies/disease-outbreak-news/item/1997_08_08-en
173	1997-08-11	https://www.who.int/emergencies/disease-outbreak-news/item/1997_08_11-en
174	1997-09-02	https://www.who.int/emergencies/disease-outbreak-news/item/1997_09_02a-en
175	1997-09-02	https://www.who.int/emergencies/disease-outbreak-news/item/1997_09_02b-en
176	1997-09-02	https://www.who.int/emergencies/disease-outbreak-news/item/1997_09_02c-en
177	1997-09-22	https://www.who.int/emergencies/disease-outbreak-news/item/1997_09_22-en
178	1997-10-02	https://www.who.int/emergencies/disease-outbreak-news/item/1997_10_02a-en
179	1997-10-02	https://www.who.int/emergencies/disease-outbreak-news/item/1997_10_02b-en
180	1997-10-10	https://www.who.int/emergencies/disease-outbreak-news/item/1997_10_10-en
181	1997-10-27	https://www.who.int/emergencies/disease-outbreak-news/item/1997_10_27-en
182	1997-10-28	https://www.who.int/emergencies/disease-outbreak-news/item/1997_10_28a-en
183	1997-10-28	https://www.who.int/emergencies/disease-outbreak-news/item/1997_10_28b-en
184	1997-10-30	https://www.who.int/emergencies/disease-outbreak-news/item/1997_10_30-en
185	1997-10-31	https://www.who.int/emergencies/disease-outbreak-news/item/1997_10_31-en
186	1997-11-14	https://www.who.int/emergencies/disease-outbreak-news/item/1997_11_14a-en
187	1997-11-14	https://www.who.int/emergencies/disease-outbreak-news/item/1997_11_14b-en

188	1997-11-19	https://www.who.int/emergencies/disease-outbreak-news/item/1997_11_19-en
189	1997-11-21	https://www.who.int/emergencies/disease-outbreak-news/item/1997_11_21a-en
190	1997-11-21	https://www.who.int/emergencies/disease-outbreak-news/item/1997_11_21b-en
191	1997-11-26	https://www.who.int/emergencies/disease-outbreak-news/item/1997_11_26a-en
192	1997-11-26	https://www.who.int/emergencies/disease-outbreak-news/item/1997_11_26b-en
193	1998-01-05	https://www.who.int/emergencies/disease-outbreak-news/item/1998_01_05-en
194	1998-01-06	https://www.who.int/emergencies/disease-outbreak-news/item/1998_01_06a-en
195	1998-01-06	https://www.who.int/emergencies/disease-outbreak-news/item/1998_01_06b-en
196	1998-01-07	https://www.who.int/emergencies/disease-outbreak-news/item/1998_01_07a-en
197	1998-01-07	https://www.who.int/emergencies/disease-outbreak-news/item/1998_01_07b-en
198	1998-01-09	https://www.who.int/emergencies/disease-outbreak-news/item/1998_01_09a-en
199	1998-01-09	https://www.who.int/emergencies/disease-outbreak-news/item/1998_01_09b-en
200	1998-01-09	https://www.who.int/emergencies/disease-outbreak-news/item/1998_01_09c-en
201	1998-01-12	https://www.who.int/emergencies/disease-outbreak-news/item/1998_01_12a-en
202	1998-01-12	https://www.who.int/emergencies/disease-outbreak-news/item/1998_01_12b-en
203	1998-01-13	https://www.who.int/emergencies/disease-outbreak-news/item/1998_01_13-en
204	1998-01-14	https://www.who.int/emergencies/disease-outbreak-news/item/1998_01_14a-en
205	1998-01-14	https://www.who.int/emergencies/disease-outbreak-news/item/1998_01_14b-en
206	1998-01-15	https://www.who.int/emergencies/disease-outbreak-news/item/1998_01_15-en
207	1998-01-16	https://www.who.int/emergencies/disease-outbreak-news/item/1998_01_16-en
208	1998-01-19	https://www.who.int/emergencies/disease-outbreak-news/item/1998_01_19-en
209	1998-01-20	https://www.who.int/emergencies/disease-outbreak-news/item/1998_01_20-en
210	1998-01-28	https://www.who.int/emergencies/disease-outbreak-news/item/1998_01_28-en
211	1998-02-03	https://www.who.int/emergencies/disease-outbreak-news/item/1998_02_03a-en
212	1998-02-03	https://www.who.int/emergencies/disease-outbreak-news/item/1998_02_03b-en
213	1998-02-10	https://www.who.int/emergencies/disease-outbreak-news/item/1998_02_10a-en
214	1998-02-10	https://www.who.int/emergencies/disease-outbreak-news/item/1998_02_10c-en
215	1998-02-13	https://www.who.int/emergencies/disease-outbreak-news/item/1998_02_13-en
216	1998-02-19	https://www.who.int/emergencies/disease-outbreak-news/item/1998_02_19-en
217	1998-02-20	https://www.who.int/emergencies/disease-outbreak-news/item/1998_02_20a-en
218	1998-02-20	https://www.who.int/emergencies/disease-outbreak-news/item/1998_02_20b-en
219	1998-02-25	https://www.who.int/emergencies/disease-outbreak-news/item/1998_02_25-en
220	1998-03-10	https://www.who.int/emergencies/disease-outbreak-news/item/1998_02_10b-en
221	1998-03-12	https://www.who.int/emergencies/disease-outbreak-news/item/1998_03_12-en
222	1998-03-16	https://www.who.int/emergencies/disease-outbreak-news/item/1998_03_16-en
223	1998-03-31	https://www.who.int/emergencies/disease-outbreak-news/item/1998_03_31-en
224	1998-04-07	https://www.who.int/emergencies/disease-outbreak-news/item/1998_04_07-en
225	1998-04-07	https://www.who.int/emergencies/disease-outbreak-news/item/1998_04_07b-en
226	1998-04-15	https://www.who.int/emergencies/disease-outbreak-news/item/1998_04_15-en

227	1998-04-22	https://www.who.int/emergencies/disease-outbreak-news/item/1998_04_22-en
228	1998-04-29	https://www.who.int/emergencies/disease-outbreak-news/item/1998_04_29-en
229	1998-05-08	https://www.who.int/emergencies/disease-outbreak-news/item/1998_05_08a-en
230	1998-05-08	https://www.who.int/emergencies/disease-outbreak-news/item/1998_05_08b-en
231	1998-05-19	https://www.who.int/emergencies/disease-outbreak-news/item/1998_05_19-en
232	1998-05-19	https://www.who.int/emergencies/disease-outbreak-news/item/1998_05_19b-en
233	1998-05-19	https://www.who.int/emergencies/disease-outbreak-news/item/1998_05_19c-en
234	1998-05-28	https://www.who.int/emergencies/disease-outbreak-news/item/1998_05_28-en
235	1998-06-08	https://www.who.int/emergencies/disease-outbreak-news/item/1998_06_08e-en
236	1998-06-17	https://www.who.int/emergencies/disease-outbreak-news/item/1998_06_17e-en
237	1998-07-03	https://www.who.int/emergencies/disease-outbreak-news/item/1998_07_03-en
238	1998-07-04	https://www.who.int/emergencies/disease-outbreak-news/item/1998_07_04-en
239	1998-07-15	https://www.who.int/emergencies/disease-outbreak-news/item/1998_07_15-en
240	1998-07-20	https://www.who.int/emergencies/disease-outbreak-news/item/1998_07_20-en
241	1998-08-03	https://www.who.int/emergencies/disease-outbreak-news/item/1998_08_03-en
242	1998-08-06	https://www.who.int/emergencies/disease-outbreak-news/item/1998_08_06-en
243	1998-08-11	https://www.who.int/emergencies/disease-outbreak-news/item/1998_08_11-en
244	1998-08-13	https://www.who.int/emergencies/disease-outbreak-news/item/1998_08_13-en
245	1998-08-14	https://www.who.int/emergencies/disease-outbreak-news/item/1998_08_14-en
246	1998-08-18	https://www.who.int/emergencies/disease-outbreak-news/item/1998_08_18-en
247	1998-08-19	https://www.who.int/emergencies/disease-outbreak-news/item/1998_08_19-en
248	1998-08-19	https://www.who.int/emergencies/disease-outbreak-news/item/1998_08_19b-en
249	1998-08-21	https://www.who.int/emergencies/disease-outbreak-news/item/1998_08_21-en
250	1998-08-24	https://www.who.int/emergencies/disease-outbreak-news/item/1998_08_24-en
251	1998-08-26	https://www.who.int/emergencies/disease-outbreak-news/item/1998_08_26-en
252	1998-08-31	https://www.who.int/emergencies/disease-outbreak-news/item/1998_08_31-en
253	1998-09-02	https://www.who.int/emergencies/disease-outbreak-news/item/1998_09_02-en
254	1998-09-09	https://www.who.int/emergencies/disease-outbreak-news/item/1998_09_09-en
255	1998-09-11	https://www.who.int/emergencies/disease-outbreak-news/item/1998_09_11-en
256	1998-09-21	https://www.who.int/emergencies/disease-outbreak-news/item/1998_09_21a-en
257	1998-09-22	https://www.who.int/emergencies/disease-outbreak-news/item/1998_09_22-en
258	1998-09-23	https://www.who.int/emergencies/disease-outbreak-news/item/1998_09_23-en
259	1998-09-30	https://www.who.int/emergencies/disease-outbreak-news/item/1998_09_30-en
260	1998-10-14	https://www.who.int/emergencies/disease-outbreak-news/item/1998_10_14-en
261	1998-10-15	https://www.who.int/emergencies/disease-outbreak-news/item/1998_10_15-en
262	1998-10-16	https://www.who.int/emergencies/disease-outbreak-news/item/1998_10_16-en
263	1998-10-22	https://www.who.int/emergencies/disease-outbreak-news/item/1998_10_22a-en
264	1998-10-22	https://www.who.int/emergencies/disease-outbreak-news/item/1998_10_22b-en
265	1998-10-22	https://www.who.int/emergencies/disease-outbreak-news/item/1998_10_22c-en

266	1998-10-23	https://www.who.int/emergencies/disease-outbreak-news/item/1998_10_23-en
267	1998-10-26	https://www.who.int/emergencies/disease-outbreak-news/item/1998_10_26-en
268	1998-11-04	https://www.who.int/emergencies/disease-outbreak-news/item/1998_11_04-en
269	1998-11-06	https://www.who.int/emergencies/disease-outbreak-news/item/1998_11_06a-en
270	1998-11-10	https://www.who.int/emergencies/disease-outbreak-news/item/1998_11_10a-en
271	1998-11-11	https://www.who.int/emergencies/disease-outbreak-news/item/1998_11_11-en
272	1998-11-23	https://www.who.int/emergencies/disease-outbreak-news/item/1998_11_23-en
273	1998-11-24	https://www.who.int/emergencies/disease-outbreak-news/item/1998_11_24-en
274	1998-11-25	https://www.who.int/emergencies/disease-outbreak-news/item/1998_11_25a-en
275	1998-11-26	https://www.who.int/emergencies/disease-outbreak-news/item/1998_11_26-en
276	1998-12-02	https://www.who.int/emergencies/disease-outbreak-news/item/1998_12_02-en
277	1998-12-04	https://www.who.int/emergencies/disease-outbreak-news/item/1998_12_04-en
278	1998-12-18	https://www.who.int/emergencies/disease-outbreak-news/item/1998_12_18-en
279	1998-12-22	https://www.who.int/emergencies/disease-outbreak-news/item/1998_12_22a-en
280	1998-12-22	https://www.who.int/emergencies/disease-outbreak-news/item/1998_12_22b-en
281	1998-12-23	https://www.who.int/emergencies/disease-outbreak-news/item/1998_12_23-en
282	1999-01-01	https://www.who.int/emergencies/disease-outbreak-news/item/2000_01_01-en
283	1999-01-06	https://www.who.int/emergencies/disease-outbreak-news/item/1999_01_06-en
284	1999-01-12	https://www.who.int/emergencies/disease-outbreak-news/item/1999_01_12-en
285	1999-01-20	https://www.who.int/emergencies/disease-outbreak-news/item/1999_01_20-en
286	1999-01-22	https://www.who.int/emergencies/disease-outbreak-news/item/1999_01_22-en
287	1999-01-27	https://www.who.int/emergencies/disease-outbreak-news/item/1999_01_27-en
288	1999-02-10	https://www.who.int/emergencies/disease-outbreak-news/item/1999_02_10-en
289	1999-02-11	https://www.who.int/emergencies/disease-outbreak-news/item/1999_02_11-en
290	1999-02-12	https://www.who.int/emergencies/disease-outbreak-news/item/1999_03_12b-en
291	1999-02-24	https://www.who.int/emergencies/disease-outbreak-news/item/1999_02_24-en
292	1999-02-26	https://www.who.int/emergencies/disease-outbreak-news/item/1999_02_26a-en
293	1999-02-26	https://www.who.int/emergencies/disease-outbreak-news/item/1999_02_26b-en
294	1999-03-01	https://www.who.int/emergencies/disease-outbreak-news/item/1999_03_01-en
295	1999-03-12	https://www.who.int/emergencies/disease-outbreak-news/item/1999_03_12a-en
296	1999-03-17	https://www.who.int/emergencies/disease-outbreak-news/item/1999_03_17a-en
297	1999-03-17	https://www.who.int/emergencies/disease-outbreak-news/item/1999_03_17b-en
298	1999-03-17	https://www.who.int/emergencies/disease-outbreak-news/item/1999_03_17c-en
299	1999-03-18	https://www.who.int/emergencies/disease-outbreak-news/item/1999_03_18a-en
300	1999-03-18	https://www.who.int/emergencies/disease-outbreak-news/item/1999_03_18b-en
301	1999-03-19	https://www.who.int/emergencies/disease-outbreak-news/item/1999_03_19a-en
302	1999-03-19	https://www.who.int/emergencies/disease-outbreak-news/item/1999_03_19b-en
303	1999-03-22	https://www.who.int/emergencies/disease-outbreak-news/item/1999_03_22a-en
304	1999-03-22	https://www.who.int/emergencies/disease-outbreak-news/item/1999_03_22b-en

305	1999-03-26	https://www.who.int/emergencies/disease-outbreak-news/item/1999_03_26a-en
306	1999-03-29	https://www.who.int/emergencies/disease-outbreak-news/item/1999_03_29-en
307	1999-04-06	https://www.who.int/emergencies/disease-outbreak-news/item/1999_04_06-en
308	1999-04-07	https://www.who.int/emergencies/disease-outbreak-news/item/1999_04_07b-en
309	1999-04-07	https://www.who.int/emergencies/disease-outbreak-news/item/1999_04_07c-en
310	1999-04-08	https://www.who.int/emergencies/disease-outbreak-news/item/1999_04_08-en
311	1999-04-13	https://www.who.int/emergencies/disease-outbreak-news/item/1999_04_13-en
312	1999-04-14	https://www.who.int/emergencies/disease-outbreak-news/item/1999_04_14a-en
313	1999-04-14	https://www.who.int/emergencies/disease-outbreak-news/item/1999_04_14b-en
314	1999-04-15	https://www.who.int/emergencies/disease-outbreak-news/item/1999_04_15-en
315	1999-04-20	https://www.who.int/emergencies/disease-outbreak-news/item/1999_04_20-en
316	1999-04-28	https://www.who.int/emergencies/disease-outbreak-news/item/1999_04_28a-en
317	1999-04-28	https://www.who.int/emergencies/disease-outbreak-news/item/1999_04_28b-en
318	1999-04-29	https://www.who.int/emergencies/disease-outbreak-news/item/1999_04_29-en
319	1999-04-30	https://www.who.int/emergencies/disease-outbreak-news/item/1999_04_30a-en
320	1999-04-30	https://www.who.int/emergencies/disease-outbreak-news/item/1999_04_30b-en
321	1999-05-05	https://www.who.int/emergencies/disease-outbreak-news/item/1999_05_05-en
322	1999-05-06	https://www.who.int/emergencies/disease-outbreak-news/item/1999_05_06-en
323	1999-05-07	https://www.who.int/emergencies/disease-outbreak-news/item/1999_04_07a-en
324	1999-05-10	https://www.who.int/emergencies/disease-outbreak-news/item/1999_05_10-en
325	1999-05-11	https://www.who.int/emergencies/disease-outbreak-news/item/1999_05_11-en
326	1999-05-12	https://www.who.int/emergencies/disease-outbreak-news/item/1999_05_12a-en
327	1999-05-12	https://www.who.int/emergencies/disease-outbreak-news/item/1999_05_12b-en
328	1999-05-14	https://www.who.int/emergencies/disease-outbreak-news/item/1999_05_14-en
329	1999-05-17	https://www.who.int/emergencies/disease-outbreak-news/item/1999_05_17-en
330	1999-05-18	https://www.who.int/emergencies/disease-outbreak-news/item/1999_05_18a-en
331	1999-05-20	https://www.who.int/emergencies/disease-outbreak-news/item/1999_05_20-en
332	1999-05-21	https://www.who.int/emergencies/disease-outbreak-news/item/1999_05_21-en
333	1999-05-28	https://www.who.int/emergencies/disease-outbreak-news/item/1999_05_28-en
334	1999-06-16	https://www.who.int/emergencies/disease-outbreak-news/item/1999_06_16-en
335	1999-06-23	https://www.who.int/emergencies/disease-outbreak-news/item/1999_06_23-en
336	1999-06-25	https://www.who.int/emergencies/disease-outbreak-news/item/1999_06_25-en
337	1999-07-14	https://www.who.int/emergencies/disease-outbreak-news/item/1999_07_14a-en
338	1999-07-20	https://www.who.int/emergencies/disease-outbreak-news/item/1999_07_20-en
339	1999-07-26	https://www.who.int/emergencies/disease-outbreak-news/item/1999_07_26-en
340	1999-07-27	https://www.who.int/emergencies/disease-outbreak-news/item/1999_07_27-en
341	1999-08-03	https://www.who.int/emergencies/disease-outbreak-news/item/1999_08_03-en
342	1999-08-04	https://www.who.int/emergencies/disease-outbreak-news/item/1999_08_04-en
343	1999-08-06	https://www.who.int/emergencies/disease-outbreak-news/item/1999_08_06-en

344	1999-08-09	https://www.who.int/emergencies/disease-outbreak-news/item/1999_08_09-en
345	1999-08-25	https://www.who.int/emergencies/disease-outbreak-news/item/1999_08_25-en
346	1999-08-30	https://www.who.int/emergencies/disease-outbreak-news/item/1999_08_30-en
347	1999-09-02	https://www.who.int/emergencies/disease-outbreak-news/item/1999_09_02-en
348	1999-09-07	https://www.who.int/emergencies/disease-outbreak-news/item/1999_09_07-en
349	1999-09-08	https://www.who.int/emergencies/disease-outbreak-news/item/1999_09_08-en
350	1999-09-10	https://www.who.int/emergencies/disease-outbreak-news/item/1999_09_10-en
351	1999-09-14	https://www.who.int/emergencies/disease-outbreak-news/item/1999_09_14a-en
352	1999-09-24	https://www.who.int/emergencies/disease-outbreak-news/item/1999_09_24-en
353	1999-10-06	https://www.who.int/emergencies/disease-outbreak-news/item/1999_10_06-en
354	1999-10-20	https://www.who.int/emergencies/disease-outbreak-news/item/1999_10_20-en
355	1999-10-21	https://www.who.int/emergencies/disease-outbreak-news/item/1999_10_21-en
356	1999-10-27	https://www.who.int/emergencies/disease-outbreak-news/item/1999_10_27-en
357	1999-11-18	https://www.who.int/emergencies/disease-outbreak-news/item/1999_11_18-en
358	1999-11-18	https://www.who.int/emergencies/disease-outbreak-news/item/1999_11_18b-en
359	1999-11-19	https://www.who.int/emergencies/disease-outbreak-news/item/1999_11_19-en
360	1999-11-23	https://www.who.int/emergencies/disease-outbreak-news/item/1999_11_23-en
361	1999-12-09	https://www.who.int/emergencies/disease-outbreak-news/item/1999_12_09-en
362	1999-12-10	https://www.who.int/emergencies/disease-outbreak-news/item/1999_12_10-en
363	1999-12-17	https://www.who.int/emergencies/disease-outbreak-news/item/1999_12_17-en
364	1999-12-22	https://www.who.int/emergencies/disease-outbreak-news/item/1999_12_22-en
365	2000-01-13	https://www.who.int/emergencies/disease-outbreak-news/item/2000_01_13-en
366	2000-01-14	https://www.who.int/emergencies/disease-outbreak-news/item/2000_01_14-en
367	2000-01-18	https://www.who.int/emergencies/disease-outbreak-news/item/2000_01_18a-en
368	2000-01-20	https://www.who.int/emergencies/disease-outbreak-news/item/2000_01_20-en
369	2000-01-24	https://www.who.int/emergencies/disease-outbreak-news/item/2000_01_24a-en
370	2000-01-28	https://www.who.int/emergencies/disease-outbreak-news/item/2000_01_28a-en
371	2000-02-04	https://www.who.int/emergencies/disease-outbreak-news/item/2000_02_04-en
372	2000-02-11	https://www.who.int/emergencies/disease-outbreak-news/item/2000_02_11-en
373	2000-02-16	https://www.who.int/emergencies/disease-outbreak-news/item/2000_02_16-en
374	2000-02-25	https://www.who.int/emergencies/disease-outbreak-news/item/2000_02_25-en
375	2000-02-29	https://www.who.int/emergencies/disease-outbreak-news/item/2000_02_29-en
376	2000-03-02	https://www.who.int/emergencies/disease-outbreak-news/item/2000_03_02-en
377	2000-03-02	https://www.who.int/emergencies/disease-outbreak-news/item/2000_03_02a-en
378	2000-03-08	https://www.who.int/emergencies/disease-outbreak-news/item/2000_03_08-en
379	2000-03-13	https://www.who.int/emergencies/disease-outbreak-news/item/2000_03_13-en
380	2000-03-14	https://www.who.int/emergencies/disease-outbreak-news/item/2000_03_14-en
381	2000-03-16	https://www.who.int/emergencies/disease-outbreak-news/item/2000_03_16-en
382	2000-03-17	https://www.who.int/emergencies/disease-outbreak-news/item/2000_03_17-en

383	2000-03-21	https://www.who.int/emergencies/disease-outbreak-news/item/2000_03_21-en
384	2000-03-31	https://www.who.int/emergencies/disease-outbreak-news/item/2000_03_31-en
385	2000-04-04	https://www.who.int/emergencies/disease-outbreak-news/item/2000_04_04-en
386	2000-04-07	https://www.who.int/emergencies/disease-outbreak-news/item/2000_04_07-en
387	2000-04-10	https://www.who.int/emergencies/disease-outbreak-news/item/2000_04_10-en
388	2000-04-11	https://www.who.int/emergencies/disease-outbreak-news/item/2000_04_11-en
389	2000-04-21	https://www.who.int/emergencies/disease-outbreak-news/item/2000_04_21a-en
390	2000-04-21	https://www.who.int/emergencies/disease-outbreak-news/item/2000_04_21b-en
391	2000-04-26	https://www.who.int/emergencies/disease-outbreak-news/item/2000_04_26-en
392	2000-04-27	https://www.who.int/emergencies/disease-outbreak-news/item/2000_04_27a-en
393	2000-05-02	https://www.who.int/emergencies/disease-outbreak-news/item/2000_05_02-en
394	2000-05-03	https://www.who.int/emergencies/disease-outbreak-news/item/2000_05_03-en
395	2000-05-04	https://www.who.int/emergencies/disease-outbreak-news/item/2000_05_04-en
396	2000-05-11	https://www.who.int/emergencies/disease-outbreak-news/item/2000_05_11-en
397	2000-05-19	https://www.who.int/emergencies/disease-outbreak-news/item/2000_05_19-en
398	2000-05-30	https://www.who.int/emergencies/disease-outbreak-news/item/2000_05_30-en
399	2000-06-19	https://www.who.int/emergencies/disease-outbreak-news/item/2000_06_19e-en
400	2000-06-20	https://www.who.int/emergencies/disease-outbreak-news/item/2000_06_20e-en
401	2000-06-20	https://www.who.int/emergencies/disease-outbreak-news/item/2000_07_20-en
402	2000-07-06	https://www.who.int/emergencies/disease-outbreak-news/item/2000_07_06-en
403	2000-07-10	https://www.who.int/emergencies/disease-outbreak-news/item/2000_07_10-en
404	2000-07-11	https://www.who.int/emergencies/disease-outbreak-news/item/2000_07_11-en
405	2000-07-25	https://www.who.int/emergencies/disease-outbreak-news/item/2000_07_25-en
406	2000-07-26	https://www.who.int/emergencies/disease-outbreak-news/item/2000_07_26-en
407	2000-08-09	https://www.who.int/emergencies/disease-outbreak-news/item/2000_08_09-en
408	2000-08-17	https://www.who.int/emergencies/disease-outbreak-news/item/2000_08_17-en
409	2000-08-24	https://www.who.int/emergencies/disease-outbreak-news/item/2000_08_24a-en
410	2000-08-24	https://www.who.int/emergencies/disease-outbreak-news/item/2000_08_24b-en
411	2000-09-08	https://www.who.int/emergencies/disease-outbreak-news/item/2000_09_08-en
412	2000-09-11	https://www.who.int/emergencies/disease-outbreak-news/item/2000_09_11-en
413	2000-09-12	https://www.who.int/emergencies/disease-outbreak-news/item/2000_09_13-en
414	2000-09-15	https://www.who.int/emergencies/disease-outbreak-news/item/2000_09_15-en
415	2000-09-18	https://www.who.int/emergencies/disease-outbreak-news/item/2000_09_18-en
416	2000-09-21	https://www.who.int/emergencies/disease-outbreak-news/item/2000_09_21a-en
417	2000-09-22	https://www.who.int/emergencies/disease-outbreak-news/item/2000_09_22-en
418	2000-09-26	https://www.who.int/emergencies/disease-outbreak-news/item/2000_09_26-en
419	2000-09-29	https://www.who.int/emergencies/disease-outbreak-news/item/2000_09_29-en
420	2000-10-03	https://www.who.int/emergencies/disease-outbreak-news/item/2000_10_03-en
421	2000-10-09	https://www.who.int/emergencies/disease-outbreak-news/item/2000_10_09-en

422	2000-10-09	https://www.who.int/emergencies/disease-outbreak-news/item/2000_10_10-en
423	2000-10-13	https://www.who.int/emergencies/disease-outbreak-news/item/2000_10_13-en
424	2000-10-16	https://www.who.int/emergencies/disease-outbreak-news/item/2000_10_16-en
425	2000-10-17	https://www.who.int/emergencies/disease-outbreak-news/item/2000_10_17-en
426	2000-10-18	https://www.who.int/emergencies/disease-outbreak-news/item/2000_10_18a-en
427	2000-10-19	https://www.who.int/emergencies/disease-outbreak-news/item/2000_10_19a-en
428	2000-10-20	https://www.who.int/emergencies/disease-outbreak-news/item/2000_10_20a-en
429	2000-10-20	https://www.who.int/emergencies/disease-outbreak-news/item/2000_10_20b-en
430	2000-10-21	https://www.who.int/emergencies/disease-outbreak-news/item/2000_10_21-en
431	2000-10-22	https://www.who.int/emergencies/disease-outbreak-news/item/2000_10_22-en
432	2000-10-23	https://www.who.int/emergencies/disease-outbreak-news/item/2000_10_23-en
433	2000-10-24	https://www.who.int/emergencies/disease-outbreak-news/item/2000_10_24-en
434	2000-10-25	https://www.who.int/emergencies/disease-outbreak-news/item/2000_10_25a-en
435	2000-10-26	https://www.who.int/emergencies/disease-outbreak-news/item/2000_10_26a-en
436	2000-10-27	https://www.who.int/emergencies/disease-outbreak-news/item/2000_10_27a-en
437	2000-10-28	https://www.who.int/emergencies/disease-outbreak-news/item/2000_10_28-en
438	2000-10-29	https://www.who.int/emergencies/disease-outbreak-news/item/2000_10_29-en
439	2000-10-30	https://www.who.int/emergencies/disease-outbreak-news/item/2000_10_30-en
440	2000-10-31	https://www.who.int/emergencies/disease-outbreak-news/item/2000_10_31-en
441	2000-11-01	https://www.who.int/emergencies/disease-outbreak-news/item/2000_11_01-en
442	2000-11-02	https://www.who.int/emergencies/disease-outbreak-news/item/2000_11_02-en
443	2000-11-03	https://www.who.int/emergencies/disease-outbreak-news/item/2000_11_03a-en
444	2000-11-03	https://www.who.int/emergencies/disease-outbreak-news/item/2000_11_03b-en
445	2000-11-05	https://www.who.int/emergencies/disease-outbreak-news/item/2000_11_05-en
446	2000-11-06	https://www.who.int/emergencies/disease-outbreak-news/item/2000_11_06-en
447	2000-11-07	https://www.who.int/emergencies/disease-outbreak-news/item/2000_11_07-en
448	2000-11-08	https://www.who.int/emergencies/disease-outbreak-news/item/2000_11_08-en
449	2000-11-09	https://www.who.int/emergencies/disease-outbreak-news/item/2000_11_09-en
450	2000-11-10	https://www.who.int/emergencies/disease-outbreak-news/item/2000_11_10a-en
451	2000-11-11	https://www.who.int/emergencies/disease-outbreak-news/item/2000_11_11-en
452	2000-11-12	https://www.who.int/emergencies/disease-outbreak-news/item/2000_11_12-en
453	2000-11-14	https://www.who.int/emergencies/disease-outbreak-news/item/2000_11_14-en
454	2000-11-15	https://www.who.int/emergencies/disease-outbreak-news/item/2000_11_15-en
455	2000-11-17	https://www.who.int/emergencies/disease-outbreak-news/item/2000_11_17-en
456	2000-11-20	https://www.who.int/emergencies/disease-outbreak-news/item/2000_11_20-en
457	2000-11-22	https://www.who.int/emergencies/disease-outbreak-news/item/2000_11_22-en
458	2000-11-24	https://www.who.int/emergencies/disease-outbreak-news/item/2000_11_24-en
459	2000-11-27	https://www.who.int/emergencies/disease-outbreak-news/item/2000_11_27-en
460	2000-11-30	https://www.who.int/emergencies/disease-outbreak-news/item/2000_11_30-en

461	2000-12-05	https://www.who.int/emergencies/disease-outbreak-news/item/2000_12_05-en
462	2000-12-06	https://www.who.int/emergencies/disease-outbreak-news/item/2000_12_06-en
463	2000-12-08	https://www.who.int/emergencies/disease-outbreak-news/item/2000_12_08-en
464	2000-12-19	https://www.who.int/emergencies/disease-outbreak-news/item/2000_12_19a-en
465	2000-12-29	https://www.who.int/emergencies/disease-outbreak-news/item/2000_12_29a-en
466	2000-12-29	https://www.who.int/emergencies/disease-outbreak-news/item/2000_12_29b-en
467	2001-01-03	https://www.who.int/emergencies/disease-outbreak-news/item/2001_01_03-en
468	2001-01-05	https://www.who.int/emergencies/disease-outbreak-news/item/2001_01_05-en
469	2001-01-09	https://www.who.int/emergencies/disease-outbreak-news/item/2001_01_09a-en
470	2001-01-16	https://www.who.int/emergencies/disease-outbreak-news/item/2001_01_16-en
471	2001-01-17	https://www.who.int/emergencies/disease-outbreak-news/item/2001_01_17-en
472	2001-01-24	https://www.who.int/emergencies/disease-outbreak-news/item/2001_01_24-en
473	2001-01-26	https://www.who.int/emergencies/disease-outbreak-news/item/2001_01_26-en
474	2001-02-05	https://www.who.int/emergencies/disease-outbreak-news/item/2001_02_05-en
475	2001-02-09	https://www.who.int/emergencies/disease-outbreak-news/item/2001_02_09a-en
476	2001-02-16	https://www.who.int/emergencies/disease-outbreak-news/item/2001_02_16-en
477	2001-02-20	https://www.who.int/emergencies/disease-outbreak-news/item/2001_02_20-en
478	2001-02-23	https://www.who.int/emergencies/disease-outbreak-news/item/2001_02_23-en
479	2001-02-28	https://www.who.int/emergencies/disease-outbreak-news/item/2001_02_28a-en
480	2001-02-28	https://www.who.int/emergencies/disease-outbreak-news/item/2001_02_28b-en
481	2001-03-05	https://www.who.int/emergencies/disease-outbreak-news/item/2001_03_05-en
482	2001-03-07	https://www.who.int/emergencies/disease-outbreak-news/item/2001_03_07-en
483	2001-03-08	https://www.who.int/emergencies/disease-outbreak-news/item/2001_03_08-en
484	2001-03-16	https://www.who.int/emergencies/disease-outbreak-news/item/2001_03_16-en
485	2001-03-19	https://www.who.int/emergencies/disease-outbreak-news/item/2001_03_19a-en
486	2001-03-20	https://www.who.int/emergencies/disease-outbreak-news/item/2001_03_20a-en
487	2001-03-26	https://www.who.int/emergencies/disease-outbreak-news/item/2001_03_26-en
488	2001-03-27	https://www.who.int/emergencies/disease-outbreak-news/item/2001_03_27-en
489	2001-03-28	https://www.who.int/emergencies/disease-outbreak-news/item/2001_03_28-en
490	2001-04-05	https://www.who.int/emergencies/disease-outbreak-news/item/2001_04_05-en
491	2001-04-09	https://www.who.int/emergencies/disease-outbreak-news/item/2001_04_09-en
492	2001-04-17	https://www.who.int/emergencies/disease-outbreak-news/item/2001_04_17-en
493	2001-04-23	https://www.who.int/emergencies/disease-outbreak-news/item/2001_04_23-en
494	2001-04-27	https://www.who.int/emergencies/disease-outbreak-news/item/2001_04_27-en
495	2001-05-16	https://www.who.int/emergencies/disease-outbreak-news/item/2001_05_16-en
496	2001-05-17	https://www.who.int/emergencies/disease-outbreak-news/item/2001_05_17-en
497	2001-05-18	https://www.who.int/emergencies/disease-outbreak-news/item/2001_05_18-en
498	2001-05-25	https://www.who.int/emergencies/disease-outbreak-news/item/2001_05_25a-en
499	2001-05-30	https://www.who.int/emergencies/disease-outbreak-news/item/2001_05_30-en

500	2001-05-31	https://www.who.int/emergencies/disease-outbreak-news/item/2001_05_31-en
501	2001-06-05	https://www.who.int/emergencies/disease-outbreak-news/item/2001_06_05e-en
502	2001-06-07	https://www.who.int/emergencies/disease-outbreak-news/item/2001_06_07e-en
503	2001-06-08	https://www.who.int/emergencies/disease-outbreak-news/item/2001_06_08e-en
504	2001-06-11	https://www.who.int/emergencies/disease-outbreak-news/item/2001_06_11e-en
505	2001-06-14	https://www.who.int/emergencies/disease-outbreak-news/item/2001_06_14a-en
506	2001-06-15	https://www.who.int/emergencies/disease-outbreak-news/item/2001_06_15e-en
507	2001-06-20	https://www.who.int/emergencies/disease-outbreak-news/item/2001_06_20e-en
508	2001-06-22	https://www.who.int/emergencies/disease-outbreak-news/item/2001_06_22e-en
509	2001-06-29	https://www.who.int/emergencies/disease-outbreak-news/item/2001_06_29e-en
510	2001-07-03	https://www.who.int/emergencies/disease-outbreak-news/item/2001_07_03-en
511	2001-07-12	https://www.who.int/emergencies/disease-outbreak-news/item/2001_07_12-en
512	2001-07-18	https://www.who.int/emergencies/disease-outbreak-news/item/2001_07_18-en
513	2001-07-26	https://www.who.int/emergencies/disease-outbreak-news/item/2001_07_26a-en
514	2001-07-26	https://www.who.int/emergencies/disease-outbreak-news/item/2001_07_26b-en
515	2001-08-06	https://www.who.int/emergencies/disease-outbreak-news/item/2001_08_06-en
516	2001-08-14	https://www.who.int/emergencies/disease-outbreak-news/item/2001_08_14-en
517	2001-08-15	https://www.who.int/emergencies/disease-outbreak-news/item/2001_08_15a-en
518	2001-08-22	https://www.who.int/emergencies/disease-outbreak-news/item/2001_08_22-en
519	2001-08-23	https://www.who.int/emergencies/disease-outbreak-news/item/2001_08_23-en
520	2001-09-04	https://www.who.int/emergencies/disease-outbreak-news/item/2001_09_04-en
521	2001-09-05	https://www.who.int/emergencies/disease-outbreak-news/item/2001_09_05a-en
522	2001-09-05	https://www.who.int/emergencies/disease-outbreak-news/item/2001_09_05b-en
523	2001-09-12	https://www.who.int/emergencies/disease-outbreak-news/item/2001_09_12-en
524	2001-09-13	https://www.who.int/emergencies/disease-outbreak-news/item/2001_09_13-en
525	2001-09-17	https://www.who.int/emergencies/disease-outbreak-news/item/2001_09_17a-en
526	2001-09-18	https://www.who.int/emergencies/disease-outbreak-news/item/2001_09_18-en
527	2001-09-25	https://www.who.int/emergencies/disease-outbreak-news/item/2001_09_25a-en
528	2001-09-26	https://www.who.int/emergencies/disease-outbreak-news/item/2001_09_26-en
529	2001-10-02	https://www.who.int/emergencies/disease-outbreak-news/item/2001_10_02-en
530	2001-10-10	https://www.who.int/emergencies/disease-outbreak-news/item/2001_10_10a-en
531	2001-10-10	https://www.who.int/emergencies/disease-outbreak-news/item/2001_10_10b-en
532	2001-10-10	https://www.who.int/emergencies/disease-outbreak-news/item/2001_10_10c-en
533	2001-10-11	https://www.who.int/emergencies/disease-outbreak-news/item/2001_10_11-en
534	2001-10-15	https://www.who.int/emergencies/disease-outbreak-news/item/2001_10_15-en
535	2001-10-17	https://www.who.int/emergencies/disease-outbreak-news/item/2001_10_17-en
536	2001-10-18	https://www.who.int/emergencies/disease-outbreak-news/item/2001_10_18-en
537	2001-10-19	https://www.who.int/emergencies/disease-outbreak-news/item/2001_10_19-en
538	2001-10-22	https://www.who.int/emergencies/disease-outbreak-news/item/2001_10_22a-en

539	2001-10-23	https://www.who.int/emergencies/disease-outbreak-news/item/2001_10_23-en
540	2001-10-24	https://www.who.int/emergencies/disease-outbreak-news/item/2001_10_24-en
541	2001-10-26	https://www.who.int/emergencies/disease-outbreak-news/item/2001_10_26-en
542	2001-10-29	https://www.who.int/emergencies/disease-outbreak-news/item/2001_10_29-en
543	2001-10-30	https://www.who.int/emergencies/disease-outbreak-news/item/2001_10_30-en
544	2001-10-31	https://www.who.int/emergencies/disease-outbreak-news/item/2001_10_31-en
545	2001-11-02	https://www.who.int/emergencies/disease-outbreak-news/item/2001_11_02-en
546	2001-11-05	https://www.who.int/emergencies/disease-outbreak-news/item/2001_11_05-en
547	2001-11-12	https://www.who.int/emergencies/disease-outbreak-news/item/2001_11_12-en
548	2001-11-15	https://www.who.int/emergencies/disease-outbreak-news/item/2001_11_15-en
549	2001-11-23	https://www.who.int/emergencies/disease-outbreak-news/item/2001_11_23-en
550	2001-11-27	https://www.who.int/emergencies/disease-outbreak-news/item/2001_11_27-en
551	2001-12-03	https://www.who.int/emergencies/disease-outbreak-news/item/2001_12_03-en
552	2001-12-05	https://www.who.int/emergencies/disease-outbreak-news/item/2001_12_05-en
553	2001-12-07	https://www.who.int/emergencies/disease-outbreak-news/item/2001_12_07-en
554	2001-12-11	https://www.who.int/emergencies/disease-outbreak-news/item/2001_12_11-en
555	2001-12-12	https://www.who.int/emergencies/disease-outbreak-news/item/2001_12_12a-en
556	2001-12-13	https://www.who.int/emergencies/disease-outbreak-news/item/2001_12_13-en
557	2001-12-16	https://www.who.int/emergencies/disease-outbreak-news/item/2001_12_16-en
558	2001-12-17	https://www.who.int/emergencies/disease-outbreak-news/item/2001_12_17-en
559	2001-12-18	https://www.who.int/emergencies/disease-outbreak-news/item/2001_12_18a-en
560	2001-12-20	https://www.who.int/emergencies/disease-outbreak-news/item/2001_12_20-en
561	2001-12-24	https://www.who.int/emergencies/disease-outbreak-news/item/2001_12_24-en
562	2001-12-28	https://www.who.int/emergencies/disease-outbreak-news/item/2001_12_28-en
563	2002-01-02	https://www.who.int/emergencies/disease-outbreak-news/item/2002_01_02-en
564	2002-01-04	https://www.who.int/emergencies/disease-outbreak-news/item/2002_01_04-en
565	2002-01-07	https://www.who.int/emergencies/disease-outbreak-news/item/2002_01_07-en
566	2002-01-09	https://www.who.int/emergencies/disease-outbreak-news/item/2002_01_09-en
567	2002-01-16	https://www.who.int/emergencies/disease-outbreak-news/item/2002_01_16-en
568	2002-01-17	https://www.who.int/emergencies/disease-outbreak-news/item/2002_01_17-en
569	2002-01-18	https://www.who.int/emergencies/disease-outbreak-news/item/2002_01_18-en
570	2002-01-21	https://www.who.int/emergencies/disease-outbreak-news/item/2002_01_21-en
571	2002-01-24	https://www.who.int/emergencies/disease-outbreak-news/item/2002_01_24-en
572	2002-01-25	https://www.who.int/emergencies/disease-outbreak-news/item/2002_01_25-en
573	2002-01-28	https://www.who.int/emergencies/disease-outbreak-news/item/2002_01_28-en
574	2002-02-06	https://www.who.int/emergencies/disease-outbreak-news/item/2002_02_06-en
575	2002-02-07	https://www.who.int/emergencies/disease-outbreak-news/item/2002_02_07-en
576	2002-02-08	https://www.who.int/emergencies/disease-outbreak-news/item/2002_02_08-en
577	2002-02-12	https://www.who.int/emergencies/disease-outbreak-news/item/2002_02_12-en

578	2002-02-15	https://www.who.int/emergencies/disease-outbreak-news/item/2002_02_15-en
579	2002-02-20	https://www.who.int/emergencies/disease-outbreak-news/item/2002_02_20-en
580	2002-02-21	https://www.who.int/emergencies/disease-outbreak-news/item/2002_02_21a-en
581	2002-02-25	https://www.who.int/emergencies/disease-outbreak-news/item/2002_02_25-en
582	2002-03-05	https://www.who.int/emergencies/disease-outbreak-news/item/2002_03_05-en
583	2002-03-06	https://www.who.int/emergencies/disease-outbreak-news/item/2002_03_06-en
584	2002-03-07	https://www.who.int/emergencies/disease-outbreak-news/item/2002_03_07-en
585	2002-03-08	https://www.who.int/emergencies/disease-outbreak-news/item/2002_03_08a-en
586	2002-03-14	https://www.who.int/emergencies/disease-outbreak-news/item/2002_03_14-en
587	2002-03-15	https://www.who.int/emergencies/disease-outbreak-news/item/2002_03_15-en
588	2002-03-22	https://www.who.int/emergencies/disease-outbreak-news/item/2002_03_22-en
589	2002-03-26	https://www.who.int/emergencies/disease-outbreak-news/item/2002_03_26-en
590	2002-04-25	https://www.who.int/emergencies/disease-outbreak-news/item/2002_04_25-en
591	2002-04-26	https://www.who.int/emergencies/disease-outbreak-news/item/2002_04_26-en
592	2002-05-03	https://www.who.int/emergencies/disease-outbreak-news/item/2002_05_03-en
593	2002-05-06	https://www.who.int/emergencies/disease-outbreak-news/item/2002_05_06-en
594	2002-05-08	https://www.who.int/emergencies/disease-outbreak-news/item/2002_05_08-en
595	2002-05-09	https://www.who.int/emergencies/disease-outbreak-news/item/2002_05_09-en
596	2002-05-17	https://www.who.int/emergencies/disease-outbreak-news/item/2002_05_17-en
597	2002-05-22	https://www.who.int/emergencies/disease-outbreak-news/item/2002_05_22-en
598	2002-06-05	https://www.who.int/emergencies/disease-outbreak-news/item/2002_06_05e-en
599	2002-06-12	https://www.who.int/emergencies/disease-outbreak-news/item/2002_06_12e-en
600	2002-06-19	https://www.who.int/emergencies/disease-outbreak-news/item/2002_06_19e-en
601	2002-06-21	https://www.who.int/emergencies/disease-outbreak-news/item/2002_06_21e-en
602	2002-06-24	https://www.who.int/emergencies/disease-outbreak-news/item/2002_06_24e-en
603	2002-06-28	https://www.who.int/emergencies/disease-outbreak-news/item/2002_06_28a-en
604	2002-06-28	https://www.who.int/emergencies/disease-outbreak-news/item/2002_06_28b-en
605	2002-07-10	https://www.who.int/emergencies/disease-outbreak-news/item/2002_07_10-en
606	2002-07-12	https://www.who.int/emergencies/disease-outbreak-news/item/2002_07_12-en
607	2002-07-17	https://www.who.int/emergencies/disease-outbreak-news/item/2002_07_17-en
608	2002-07-19	https://www.who.int/emergencies/disease-outbreak-news/item/2002_07_19a-en
609	2002-07-19	https://www.who.int/emergencies/disease-outbreak-news/item/2002_07_19b-en
610	2002-07-24	https://www.who.int/emergencies/disease-outbreak-news/item/2002_07_24-en
611	2002-08-05	https://www.who.int/emergencies/disease-outbreak-news/item/2002_08_05-en
612	2002-08-07	https://www.who.int/emergencies/disease-outbreak-news/item/2002_08_07-en
613	2002-08-13	https://www.who.int/emergencies/disease-outbreak-news/item/2002_08_13a-en
614	2002-08-14	https://www.who.int/emergencies/disease-outbreak-news/item/2002_08_14-en
615	2002-08-16	https://www.who.int/emergencies/disease-outbreak-news/item/2002_08_16a-en
616	2002-08-21	https://www.who.int/emergencies/disease-outbreak-news/item/2002_03_21a-en

617	2002-08-23	https://www.who.int/emergencies/disease-outbreak-news/item/2002_08_23a-en
618	2002-08-27	https://www.who.int/emergencies/disease-outbreak-news/item/2002_08_27-en
619	2002-08-28	https://www.who.int/emergencies/disease-outbreak-news/item/2002_08_28-en
620	2002-08-29	https://www.who.int/emergencies/disease-outbreak-news/item/2002_08_29-en
621	2002-08-30	https://www.who.int/emergencies/disease-outbreak-news/item/2002_08_30a-en
622	2002-09-03	https://www.who.int/emergencies/disease-outbreak-news/item/2002_09_03a-en
623	2002-09-04	https://www.who.int/emergencies/disease-outbreak-news/item/2002_09_04-en
624	2002-09-06	https://www.who.int/emergencies/disease-outbreak-news/item/2002_09_06-en
625	2002-09-12	https://www.who.int/emergencies/disease-outbreak-news/item/2002_09_12a-en
626	2002-09-12	https://www.who.int/emergencies/disease-outbreak-news/item/2002_09_12b-en
627	2002-09-13	https://www.who.int/emergencies/disease-outbreak-news/item/2002_09_13a-en
628	2002-09-16	https://www.who.int/emergencies/disease-outbreak-news/item/2002_09_16-en
629	2002-09-17	https://www.who.int/emergencies/disease-outbreak-news/item/2002_09_17-en
630	2002-09-18	https://www.who.int/emergencies/disease-outbreak-news/item/2002_09_18-en
631	2002-09-20	https://www.who.int/emergencies/disease-outbreak-news/item/2002_09_20-en
632	2002-09-24	https://www.who.int/emergencies/disease-outbreak-news/item/2002_09_24-en
633	2002-10-01	https://www.who.int/emergencies/disease-outbreak-news/item/2002_10_01a-en
634	2002-10-02	https://www.who.int/emergencies/disease-outbreak-news/item/2002_10_02-en
635	2002-10-04	https://www.who.int/emergencies/disease-outbreak-news/item/2002_10_04-en
636	2002-10-11	https://www.who.int/emergencies/disease-outbreak-news/item/2002_10_11-en
637	2002-10-15	https://www.who.int/emergencies/disease-outbreak-news/item/2002_10_15a-en
638	2002-10-18	https://www.who.int/emergencies/disease-outbreak-news/item/2002_10_18-en
639	2002-10-22	https://www.who.int/emergencies/disease-outbreak-news/item/2002_10_22a-en
640	2002-10-24	https://www.who.int/emergencies/disease-outbreak-news/item/2002_10_24a-en
641	2002-10-25	https://www.who.int/emergencies/disease-outbreak-news/item/2002_10_25-en
642	2002-10-30	https://www.who.int/emergencies/disease-outbreak-news/item/2002_10_30a-en
643	2002-11-01	https://www.who.int/emergencies/disease-outbreak-news/item/2002_11_01-en
644	2002-11-06	https://www.who.int/emergencies/disease-outbreak-news/item/2002_11_06-en
645	2002-11-13	https://www.who.int/emergencies/disease-outbreak-news/item/2002_11_13-en
646	2002-11-14	https://www.who.int/emergencies/disease-outbreak-news/item/2002_11_14-en
647	2002-11-15	https://www.who.int/emergencies/disease-outbreak-news/item/2002_11_15-en
648	2002-11-20	https://www.who.int/emergencies/disease-outbreak-news/item/2002_11_20-en
649	2002-11-29	https://www.who.int/emergencies/disease-outbreak-news/item/2002_11_29a-en
650	2002-12-12	https://www.who.int/emergencies/disease-outbreak-news/item/2002_12_12-en
651	2002-12-20	https://www.who.int/emergencies/disease-outbreak-news/item/2002_12_20-en
652	2002-12-31	https://www.who.int/emergencies/disease-outbreak-news/item/2001_12_31-en
653	2003-01-08	https://www.who.int/emergencies/disease-outbreak-news/item/2003_01_08a-en
654	2003-01-17	https://www.who.int/emergencies/disease-outbreak-news/item/2003_01_17-en
655	2003-01-21	https://www.who.int/emergencies/disease-outbreak-news/item/2003_01_21-en

656	2003-02-03	https://www.who.int/emergencies/disease-outbreak-news/item/2003_02_03-en
657	2003-02-04	https://www.who.int/emergencies/disease-outbreak-news/item/2003_02_04-en
658	2003-02-07	https://www.who.int/emergencies/disease-outbreak-news/item/2003_02_07a-en
659	2003-02-10	https://www.who.int/emergencies/disease-outbreak-news/item/2003_02_10a-en
660	2003-02-11	https://www.who.int/emergencies/disease-outbreak-news/item/2003_02_11-en
661	2003-02-12	https://www.who.int/emergencies/disease-outbreak-news/item/2003_02_12a-en
662	2003-02-12	https://www.who.int/emergencies/disease-outbreak-news/item/2003_02_12b-en
663	2003-02-14	https://www.who.int/emergencies/disease-outbreak-news/item/2003_02_14a-en
664	2003-02-18	https://www.who.int/emergencies/disease-outbreak-news/item/2003_02_18-en
665	2003-02-19	https://www.who.int/emergencies/disease-outbreak-news/item/2003_2_19-en
666	2003-02-20	https://www.who.int/emergencies/disease-outbreak-news/item/2003_02_20-en
667	2003-02-20	https://www.who.int/emergencies/disease-outbreak-news/item/2003_2_20-en
668	2003-02-26	https://www.who.int/emergencies/disease-outbreak-news/item/2003_02_26-en
669	2003-02-27	https://www.who.int/emergencies/disease-outbreak-news/item/2003_02_27a-en
670	2003-02-28	https://www.who.int/emergencies/disease-outbreak-news/item/2003_02_28-en
671	2003-03-07	https://www.who.int/emergencies/disease-outbreak-news/item/2003_03_7a-en
672	2003-03-11	https://www.who.int/emergencies/disease-outbreak-news/item/2003_03_11-en
673	2003-03-12	https://www.who.int/emergencies/disease-outbreak-news/item/2003_03_12-en
674	2003-03-13	https://www.who.int/emergencies/disease-outbreak-news/item/2003_03_13-en
675	2003-03-14	https://www.who.int/emergencies/disease-outbreak-news/item/2003_03_14-en
676	2003-03-16	https://www.who.int/emergencies/disease-outbreak-news/item/2003_03_16-en
677	2003-03-17	https://www.who.int/emergencies/disease-outbreak-news/item/2003_03_17-en
678	2003-03-18	https://www.who.int/emergencies/disease-outbreak-news/item/2003_03_18-en
679	2003-03-19	https://www.who.int/emergencies/disease-outbreak-news/item/2003_03_19-en
680	2003-03-20	https://www.who.int/emergencies/disease-outbreak-news/item/2003_03_20-en
681	2003-03-21	https://www.who.int/emergencies/disease-outbreak-news/item/2003_03_21-en
682	2003-03-22	https://www.who.int/emergencies/disease-outbreak-news/item/2003_03_22-en
683	2003-03-24	https://www.who.int/emergencies/disease-outbreak-news/item/2003_03_24a-en
684	2003-03-24	https://www.who.int/emergencies/disease-outbreak-news/item/2003_03_24B-en
685	2003-03-25	https://www.who.int/emergencies/disease-outbreak-news/item/2003_03_25-en
686	2003-03-26	https://www.who.int/emergencies/disease-outbreak-news/item/2003_03_26-en
687	2003-03-27	https://www.who.int/emergencies/disease-outbreak-news/item/2003_03_27b-en
688	2003-03-28	https://www.who.int/emergencies/disease-outbreak-news/item/2003_03_28-en
689	2003-03-29	https://www.who.int/emergencies/disease-outbreak-news/item/2003_03_29-en
690	2003-03-31	https://www.who.int/emergencies/disease-outbreak-news/item/2003_03_31-en
691	2003-04-01	https://www.who.int/emergencies/disease-outbreak-news/item/2003_04_01-en
692	2003-04-02	https://www.who.int/emergencies/disease-outbreak-news/item/2003_02_02a-en
693	2003-04-02	https://www.who.int/emergencies/disease-outbreak-news/item/2003_04_02c-en
694	2003-04-02	https://www.who.int/emergencies/disease-outbreak-news/item/2003_04_02b-en

695	2003-04-03	https://www.who.int/emergencies/disease-outbreak-news/item/2003_04_03a-en
696	2003-04-04	https://www.who.int/emergencies/disease-outbreak-news/item/2003_04_04-en
697	2003-04-05	https://www.who.int/emergencies/disease-outbreak-news/item/2003_04_05-en
698	2003-04-07	https://www.who.int/emergencies/disease-outbreak-news/item/2003_04_07-en
699	2003-04-08	https://www.who.int/emergencies/disease-outbreak-news/item/2003_04_08-en
700	2003-04-09	https://www.who.int/emergencies/disease-outbreak-news/item/2003_04_09-en
701	2003-04-10	https://www.who.int/emergencies/disease-outbreak-news/item/2003_04_10-en
702	2003-04-11	https://www.who.int/emergencies/disease-outbreak-news/item/2003_04_11-en
703	2003-04-12	https://www.who.int/emergencies/disease-outbreak-news/item/2003_04_12-en
704	2003-04-14	https://www.who.int/emergencies/disease-outbreak-news/item/2003_04_14a-en
705	2003-04-15	https://www.who.int/emergencies/disease-outbreak-news/item/2003_04_15-en
706	2003-04-16	https://www.who.int/emergencies/disease-outbreak-news/item/2003_04_16-en
707	2003-04-17	https://www.who.int/emergencies/disease-outbreak-news/item/2003_04_17-en
708	2003-04-18	https://www.who.int/emergencies/disease-outbreak-news/item/2003_04_18-en
709	2003-04-19	https://www.who.int/emergencies/disease-outbreak-news/item/2003_04_19-en
710	2003-04-21	https://www.who.int/emergencies/disease-outbreak-news/item/2003_04_21-en
711	2003-04-22	https://www.who.int/emergencies/disease-outbreak-news/item/2003_04_22-en
712	2003-04-23	https://www.who.int/emergencies/disease-outbreak-news/item/2003_04_23-en
713	2003-04-24	https://www.who.int/emergencies/disease-outbreak-news/item/2003_04_24a-en
714	2003-04-25	https://www.who.int/emergencies/disease-outbreak-news/item/2003_04_25-en
715	2003-04-26	https://www.who.int/emergencies/disease-outbreak-news/item/2003_04_26-en
716	2003-04-28	https://www.who.int/emergencies/disease-outbreak-news/item/2003_04_28-en
717	2003-04-29	https://www.who.int/emergencies/disease-outbreak-news/item/2003_04_29-en
718	2003-04-30	https://www.who.int/emergencies/disease-outbreak-news/item/2003_04_30-en
719	2003-05-01	https://www.who.int/emergencies/disease-outbreak-news/item/2003_05_01-en
720	2003-05-02	https://www.who.int/emergencies/disease-outbreak-news/item/2003_05_02a-en
721	2003-05-03	https://www.who.int/emergencies/disease-outbreak-news/item/2003_05_03-en
722	2003-05-05	https://www.who.int/emergencies/disease-outbreak-news/item/2003_05_05-en
723	2003-05-06	https://www.who.int/emergencies/disease-outbreak-news/item/2003_05_06-en
724	2003-05-07	https://www.who.int/emergencies/disease-outbreak-news/item/2003_05_07a-en
725	2003-05-08	https://www.who.int/emergencies/disease-outbreak-news/item/2003_05_08a-en
726	2003-05-09	https://www.who.int/emergencies/disease-outbreak-news/item/2003_05_09b-en
727	2003-05-10	https://www.who.int/emergencies/disease-outbreak-news/item/2003_05_10-en
728	2003-05-12	https://www.who.int/emergencies/disease-outbreak-news/item/2003_05_12-en
729	2003-05-13	https://www.who.int/emergencies/disease-outbreak-news/item/2003_05_13b-en
730	2003-05-13	https://www.who.int/emergencies/disease-outbreak-news/item/2003_05_13a-en
731	2003-05-14	https://www.who.int/emergencies/disease-outbreak-news/item/2003_05_14a-en
732	2003-05-15	https://www.who.int/emergencies/disease-outbreak-news/item/2003_05_15-en
733	2003-05-16	https://www.who.int/emergencies/disease-outbreak-news/item/2003_05_16-en

734	2003-05-17	https://www.who.int/emergencies/disease-outbreak-news/item/2003_05_17-en
735	2003-05-19	https://www.who.int/emergencies/disease-outbreak-news/item/2003_05_19a-en
736	2003-05-20	https://www.who.int/emergencies/disease-outbreak-news/item/2003_05_20-en
737	2003-05-21	https://www.who.int/emergencies/disease-outbreak-news/item/2003_05_21a-en
738	2003-05-22	https://www.who.int/emergencies/disease-outbreak-news/item/2003_05_22-en
739	2003-05-23	https://www.who.int/emergencies/disease-outbreak-news/item/2003_05_23a-en
740	2003-05-23	https://www.who.int/emergencies/disease-outbreak-news/item/2003_05_23b-en
741	2003-05-24	https://www.who.int/emergencies/disease-outbreak-news/item/2003_05_24-en
742	2003-05-26	https://www.who.int/emergencies/disease-outbreak-news/item/2003_05_26-en
743	2003-05-27	https://www.who.int/emergencies/disease-outbreak-news/item/2003_05_27a-en
744	2003-05-28	https://www.who.int/emergencies/disease-outbreak-news/item/2003_05_28-en
745	2003-05-29	https://www.who.int/emergencies/disease-outbreak-news/item/2003_05_29-en
746	2003-05-30	https://www.who.int/emergencies/disease-outbreak-news/item/2003_05_30a-en
747	2003-06-02	https://www.who.int/emergencies/disease-outbreak-news/item/2003_06_02a-en
748	2003-06-03	https://www.who.int/emergencies/disease-outbreak-news/item/2003_06_03-en
749	2003-06-04	https://www.who.int/emergencies/disease-outbreak-news/item/2003_06_04-en
750	2003-06-05	https://www.who.int/emergencies/disease-outbreak-news/item/2003_06_05-en
751	2003-06-06	https://www.who.int/emergencies/disease-outbreak-news/item/2003_06_06-en
752	2003-06-09	https://www.who.int/emergencies/disease-outbreak-news/item/2003_06_09-en
753	2003-06-10	https://www.who.int/emergencies/disease-outbreak-news/item/2003_06_10-en
754	2003-06-11	https://www.who.int/emergencies/disease-outbreak-news/item/2003_06_11a-en
755	2003-06-12	https://www.who.int/emergencies/disease-outbreak-news/item/2003_06_12-en
756	2003-06-13	https://www.who.int/emergencies/disease-outbreak-news/item/2003_06_13-en
757	2003-06-13	https://www.who.int/emergencies/disease-outbreak-news/item/2003_06_13a-en
758	2003-06-16	https://www.who.int/emergencies/disease-outbreak-news/item/2003_06_16a-en
759	2003-06-18	https://www.who.int/emergencies/disease-outbreak-news/item/2003_06_18-en
760	2003-06-19	https://www.who.int/emergencies/disease-outbreak-news/item/2003_06_17A-en
761	2003-06-19	https://www.who.int/emergencies/disease-outbreak-news/item/2003_06_19-en
762	2003-06-20	https://www.who.int/emergencies/disease-outbreak-news/item/2003_06_20-en
763	2003-06-23	https://www.who.int/emergencies/disease-outbreak-news/item/2003_06_23-en
764	2003-06-24	https://www.who.int/emergencies/disease-outbreak-news/item/2003_06_24-en
765	2003-06-24	https://www.who.int/emergencies/disease-outbreak-news/item/2003_06_24a-en
766	2003-06-25	https://www.who.int/emergencies/disease-outbreak-news/item/2003_06_25-en
767	2003-06-26	https://www.who.int/emergencies/disease-outbreak-news/item/2003_06_26-en
768	2003-06-27	https://www.who.int/emergencies/disease-outbreak-news/item/2003_06_27-en
769	2003-06-30	https://www.who.int/emergencies/disease-outbreak-news/item/2003_06_30-en
770	2003-07-01	https://www.who.int/emergencies/disease-outbreak-news/item/2003_07_01-en
771	2003-07-02	https://www.who.int/emergencies/disease-outbreak-news/item/2003_07_02-en
772	2003-07-03	https://www.who.int/emergencies/disease-outbreak-news/item/2003_07_03-en

773	2003-07-03	https://www.who.int/emergencies/disease-outbreak-news/item/2003_07_03a-en
774	2003-07-03	https://www.who.int/emergencies/disease-outbreak-news/item/2003_07_03b-en
775	2003-07-04	https://www.who.int/emergencies/disease-outbreak-news/item/2003_07_04-en
776	2003-07-05	https://www.who.int/emergencies/disease-outbreak-news/item/2003_07_05-en
777	2003-07-10	https://www.who.int/emergencies/disease-outbreak-news/item/2003_07_10-en
778	2003-07-15	https://www.who.int/emergencies/disease-outbreak-news/item/2003_07_15-en
779	2003-07-28	https://www.who.int/emergencies/disease-outbreak-news/item/2003_07_28-en
780	2003-08-13	https://www.who.int/emergencies/disease-outbreak-news/item/2003_08_13-en
781	2003-08-14	https://www.who.int/emergencies/disease-outbreak-news/item/2003_08_14a-en
782	2003-08-18	https://www.who.int/emergencies/disease-outbreak-news/item/2003_08_18a-en
783	2003-08-19	https://www.who.int/emergencies/disease-outbreak-news/item/2003_08_19-en
784	2003-08-25	https://www.who.int/emergencies/disease-outbreak-news/item/2003_08_25a-en
785	2003-08-29	https://www.who.int/emergencies/disease-outbreak-news/item/2003_08_29-en
786	2003-09-02	https://www.who.int/emergencies/disease-outbreak-news/item/2003_09_02-en
787	2003-09-10	https://www.who.int/emergencies/disease-outbreak-news/item/2003_09_10-en
788	2003-09-16	https://www.who.int/emergencies/disease-outbreak-news/item/2003_09_16-en
789	2003-09-24	https://www.who.int/emergencies/disease-outbreak-news/item/2003_09_24-en
790	2003-09-30	https://www.who.int/emergencies/disease-outbreak-news/item/2003_09_30a-en
791	2003-10-09	https://www.who.int/emergencies/disease-outbreak-news/item/2003_10_09-en
792	2003-10-10	https://www.who.int/emergencies/disease-outbreak-news/item/2003_10_10-en
793	2003-10-29	https://www.who.int/emergencies/disease-outbreak-news/item/2003_10_29-en
794	2003-10-30	https://www.who.int/emergencies/disease-outbreak-news/item/2003_10_30-en
795	2003-11-05	https://www.who.int/emergencies/disease-outbreak-news/item/2003_11_05a-en
796	2003-11-07	https://www.who.int/emergencies/disease-outbreak-news/item/2003_11_07-en
797	2003-11-12	https://www.who.int/emergencies/disease-outbreak-news/item/2003_11_12-en
798	2003-11-17	https://www.who.int/emergencies/disease-outbreak-news/item/2003_11_17-en
799	2003-11-20	https://www.who.int/emergencies/disease-outbreak-news/item/2003_11_20-en
800	2003-11-21	https://www.who.int/emergencies/disease-outbreak-news/item/2003_11_21a-en
801	2003-11-25	https://www.who.int/emergencies/disease-outbreak-news/item/2003_11_25a-en
802	2003-12-03	https://www.who.int/emergencies/disease-outbreak-news/item/2003_12_03-en
803	2003-12-04	https://www.who.int/emergencies/disease-outbreak-news/item/2003_12_04a-en
804	2003-12-10	https://www.who.int/emergencies/disease-outbreak-news/item/2003_12_10a-en
805	2003-12-12	https://www.who.int/emergencies/disease-outbreak-news/item/2003_12_12-en
806	2003-12-17	https://www.who.int/emergencies/disease-outbreak-news/item/2003_12_17a-en
807	2003-12-17	https://www.who.int/emergencies/disease-outbreak-news/item/2003_12_17c-en
808	2003-12-23	https://www.who.int/emergencies/disease-outbreak-news/item/2003_12_23-en
809	2003-12-28	https://www.who.int/emergencies/disease-outbreak-news/item/2003_12_28-en
810	2003-12-30	https://www.who.int/emergencies/disease-outbreak-news/item/2003_12_30-en
811	2004-01-05	https://www.who.int/emergencies/disease-outbreak-news/item/2004_01_05-en

812	2004-01-06	https://www.who.int/emergencies/disease-outbreak-news/item/2004_01_06-en
813	2004-01-07	https://www.who.int/emergencies/disease-outbreak-news/item/2004_01_07-en
814	2004-01-08	https://www.who.int/emergencies/disease-outbreak-news/item/2004_01_08-en
815	2004-01-13	https://www.who.int/emergencies/disease-outbreak-news/item/2004_01_13a-en
816	2004-01-14	https://www.who.int/emergencies/disease-outbreak-news/item/2004_01_14-en
817	2004-01-16	https://www.who.int/emergencies/disease-outbreak-news/item/2004_01_16-en
818	2004-01-19	https://www.who.int/emergencies/disease-outbreak-news/item/2004_01_19-en
819	2004-01-20	https://www.who.int/emergencies/disease-outbreak-news/item/2004_01_20-en
820	2004-01-21	https://www.who.int/emergencies/disease-outbreak-news/item/2004_01_21-en
821	2004-01-22	https://www.who.int/emergencies/disease-outbreak-news/item/2004_01_22-en
822	2004-01-23	https://www.who.int/emergencies/disease-outbreak-news/item/2004_01_23-en
823	2004-01-24	https://www.who.int/emergencies/disease-outbreak-news/item/2004_01_24-en
824	2004-01-26	https://www.who.int/emergencies/disease-outbreak-news/item/2004_01_26-en
825	2004-01-27	https://www.who.int/emergencies/disease-outbreak-news/item/2004_01_27a-en
826	2004-01-27	https://www.who.int/emergencies/disease-outbreak-news/item/2004_01_27b-en
827	2004-01-28	https://www.who.int/emergencies/disease-outbreak-news/item/2004_01_28a-en
828	2004-01-28	https://www.who.int/emergencies/disease-outbreak-news/item/2004_01_28-en
829	2004-01-29	https://www.who.int/emergencies/disease-outbreak-news/item/2004_01_29-en
830	2004-01-30	https://www.who.int/emergencies/disease-outbreak-news/item/2004_01_30-en
831	2004-01-31	https://www.who.int/emergencies/disease-outbreak-news/item/2004_01_31-en
832	2004-02-01	https://www.who.int/emergencies/disease-outbreak-news/item/2004_02_01-en
833	2004-02-02	https://www.who.int/emergencies/disease-outbreak-news/item/2004_02_02-en
834	2004-02-03	https://www.who.int/emergencies/disease-outbreak-news/item/2004_02_03-en
835	2004-02-04	https://www.who.int/emergencies/disease-outbreak-news/item/2004_02_04-en
836	2004-02-05	https://www.who.int/emergencies/disease-outbreak-news/item/2004_02_05-en
837	2004-02-06	https://www.who.int/emergencies/disease-outbreak-news/item/2004_02_06-en
838	2004-02-09	https://www.who.int/emergencies/disease-outbreak-news/item/2004_02_09-en
839	2004-02-11	https://www.who.int/emergencies/disease-outbreak-news/item/2004_02_11-en
840	2004-02-12	https://www.who.int/emergencies/disease-outbreak-news/item/2004_02_12-en
841	2004-02-12	https://www.who.int/emergencies/disease-outbreak-news/item/2004_02_12a-en
842	2004-02-13	https://www.who.int/emergencies/disease-outbreak-news/item/2004_02_13-en
843	2004-02-16	https://www.who.int/emergencies/disease-outbreak-news/item/2004_02_16-en
844	2004-02-17	https://www.who.int/emergencies/disease-outbreak-news/item/2004_02_17-en
845	2004-02-18	https://www.who.int/emergencies/disease-outbreak-news/item/2004_02_18a-en
846	2004-02-19	https://www.who.int/emergencies/disease-outbreak-news/item/2004_02_19-en
847	2004-02-20	https://www.who.int/emergencies/disease-outbreak-news/item/2004_02_20-en
848	2004-02-23	https://www.who.int/emergencies/disease-outbreak-news/item/2004_02_23-en
849	2004-02-25	https://www.who.int/emergencies/disease-outbreak-news/item/2004_02_25a-en
850	2004-02-26	https://www.who.int/emergencies/disease-outbreak-news/item/2004_02_26a-en

851	2004-02-27	https://www.who.int/emergencies/disease-outbreak-news/item/2004_02_27-en
852	2004-03-02	https://www.who.int/emergencies/disease-outbreak-news/item/2004_03_02-en
853	2004-03-04	https://www.who.int/emergencies/disease-outbreak-news/item/2004_03_04-en
854	2004-03-05	https://www.who.int/emergencies/disease-outbreak-news/item/2004_03_05-en
855	2004-03-09	https://www.who.int/emergencies/disease-outbreak-news/item/2004_03_09-en
856	2004-03-11	https://www.who.int/emergencies/disease-outbreak-news/item/2004_03_11-en
857	2004-03-17	https://www.who.int/emergencies/disease-outbreak-news/item/2004_03_17-en
858	2004-03-22	https://www.who.int/emergencies/disease-outbreak-news/item/2004_03_22a-en
859	2004-03-23	https://www.who.int/emergencies/disease-outbreak-news/item/2004_03_23-en
860	2004-03-26	https://www.who.int/emergencies/disease-outbreak-news/item/2004_03_26-en
861	2004-03-26	https://www.who.int/emergencies/disease-outbreak-news/item/2004_03_26a-en
862	2004-03-29	https://www.who.int/emergencies/disease-outbreak-news/item/2004_03_29-en
863	2004-04-05	https://www.who.int/emergencies/disease-outbreak-news/item/2004_04_05-en
864	2004-04-08	https://www.who.int/emergencies/disease-outbreak-news/item/2004_04_08a-en
865	2004-04-08	https://www.who.int/emergencies/disease-outbreak-news/item/2004_04_08b-en
866	2004-04-20	https://www.who.int/emergencies/disease-outbreak-news/item/2004_04_20a-en
867	2004-04-22	https://www.who.int/emergencies/disease-outbreak-news/item/2004_04_22-en
868	2004-04-23	https://www.who.int/emergencies/disease-outbreak-news/item/2004_04_23-en
869	2004-04-26	https://www.who.int/emergencies/disease-outbreak-news/item/2004_04_26-en
870	2004-04-28	https://www.who.int/emergencies/disease-outbreak-news/item/2004_04_28-en
871	2004-04-29	https://www.who.int/emergencies/disease-outbreak-news/item/2004_04_29-en
872	2004-04-30	https://www.who.int/emergencies/disease-outbreak-news/item/2004_04_30-en
873	2004-05-05	https://www.who.int/emergencies/disease-outbreak-news/item/2004_05_05-en
874	2004-05-11	https://www.who.int/emergencies/disease-outbreak-news/item/2004_05_11a-en
875	2004-05-18	https://www.who.int/emergencies/disease-outbreak-news/item/2004_05_18a-en
876	2004-05-24	https://www.who.int/emergencies/disease-outbreak-news/item/2004_05_24-en
877	2004-05-26	https://www.who.int/emergencies/disease-outbreak-news/item/2004_05_26-en
878	2004-06-01	https://www.who.int/emergencies/disease-outbreak-news/item/2004_06_01a-en
879	2004-06-02	https://www.who.int/emergencies/disease-outbreak-news/item/2004_06_02a-en
880	2004-06-07	https://www.who.int/emergencies/disease-outbreak-news/item/2004_06_07-en
881	2004-06-11	https://www.who.int/emergencies/disease-outbreak-news/item/2004_06_11-en
882	2004-06-15	https://www.who.int/emergencies/disease-outbreak-news/item/2004_06_15-en
883	2004-06-17	https://www.who.int/emergencies/disease-outbreak-news/item/2004_06_17a-en
884	2004-06-22	https://www.who.int/emergencies/disease-outbreak-news/item/2004_06_22-en
885	2004-07-06	https://www.who.int/emergencies/disease-outbreak-news/item/2004_07_06-en
886	2004-07-08	https://www.who.int/emergencies/disease-outbreak-news/item/2004_07_08-en
887	2004-07-14	https://www.who.int/emergencies/disease-outbreak-news/item/2004_07_14-en
888	2004-07-16	https://www.who.int/emergencies/disease-outbreak-news/item/2004_07_16-en
889	2004-07-30	https://www.who.int/emergencies/disease-outbreak-news/item/2004_07_30-en

890	2004-08-07	https://www.who.int/emergencies/disease-outbreak-news/item/2004_08_07-en
891	2004-08-10	https://www.who.int/emergencies/disease-outbreak-news/item/2004_08_10-en
892	2004-08-12	https://www.who.int/emergencies/disease-outbreak-news/item/2004_08_12-en
893	2004-08-13	https://www.who.int/emergencies/disease-outbreak-news/item/2004_08_13-en
894	2004-08-18	https://www.who.int/emergencies/disease-outbreak-news/item/2004_08_18-en
895	2004-08-19	https://www.who.int/emergencies/disease-outbreak-news/item/2004_08_19-en
896	2004-08-20	https://www.who.int/emergencies/disease-outbreak-news/item/2004_08_20-en
897	2004-08-25	https://www.who.int/emergencies/disease-outbreak-news/item/2004_08_25-en
898	2004-08-30	https://www.who.int/emergencies/disease-outbreak-news/item/2004_08_30-en
899	2004-08-31	https://www.who.int/emergencies/disease-outbreak-news/item/2004_08_31-en
900	2004-09-01	https://www.who.int/emergencies/disease-outbreak-news/item/2004_09_01a-en
901	2004-09-06	https://www.who.int/emergencies/disease-outbreak-news/item/2004_09_06-en
902	2004-09-07	https://www.who.int/emergencies/disease-outbreak-news/item/2004_09_07-en
903	2004-09-08	https://www.who.int/emergencies/disease-outbreak-news/item/2004_09_08-en
904	2004-09-09	https://www.who.int/emergencies/disease-outbreak-news/item/2004_09_09-en
905	2004-09-14	https://www.who.int/emergencies/disease-outbreak-news/item/2004_09_14-en
906	2004-09-15	https://www.who.int/emergencies/disease-outbreak-news/item/2004_09_15-en
907	2004-09-27	https://www.who.int/emergencies/disease-outbreak-news/item/2004_09_27a-en
908	2004-09-28	https://www.who.int/emergencies/disease-outbreak-news/item/2004_09_28a-en
909	2004-10-04	https://www.who.int/emergencies/disease-outbreak-news/item/2004_10_04-en
910	2004-10-25	https://www.who.int/emergencies/disease-outbreak-news/item/2004_10_25-en
911	2004-10-29	https://www.who.int/emergencies/disease-outbreak-news/item/2004_10_29-en
912	2004-11-02	https://www.who.int/emergencies/disease-outbreak-news/item/2004_11_02-en
913	2004-11-12	https://www.who.int/emergencies/disease-outbreak-news/item/2004_11_12-en
914	2004-11-24	https://www.who.int/emergencies/disease-outbreak-news/item/2004_11_24-en
915	2004-12-03	https://www.who.int/emergencies/disease-outbreak-news/item/2004_12_03-en
916	2004-12-15	https://www.who.int/emergencies/disease-outbreak-news/item/2004_12_15-en
917	2004-12-30	https://www.who.int/emergencies/disease-outbreak-news/item/2004_12_30-en
918	2005-01-06	https://www.who.int/emergencies/disease-outbreak-news/item/2005_01_06a-en
919	2005-01-07	https://www.who.int/emergencies/disease-outbreak-news/item/2005_01_07-en
920	2005-01-11	https://www.who.int/emergencies/disease-outbreak-news/item/2005_01_11-en
921	2005-01-11	https://www.who.int/emergencies/disease-outbreak-news/item/2005_01_11a-en
922	2005-01-13	https://www.who.int/emergencies/disease-outbreak-news/item/2005_01_13-en
923	2005-01-14	https://www.who.int/emergencies/disease-outbreak-news/item/2005_01_14-en
924	2005-01-19	https://www.who.int/emergencies/disease-outbreak-news/item/2005_01_19a-en
925	2005-01-19	https://www.who.int/emergencies/disease-outbreak-news/item/2005_01_19b-en
926	2005-01-20	https://www.who.int/emergencies/disease-outbreak-news/item/2005_01_20-en
927	2005-01-21	https://www.who.int/emergencies/disease-outbreak-news/item/2005_01_21-en
928	2005-01-26	https://www.who.int/emergencies/disease-outbreak-news/item/2005_01_26-en

929	2005-01-28	https://www.who.int/emergencies/disease-outbreak-news/item/2005_01_28-en
930	2005-01-28	https://www.who.int/emergencies/disease-outbreak-news/item/2005_01_28a-en
931	2005-01-28	https://www.who.int/emergencies/disease-outbreak-news/item/2005_01_28b-en
932	2005-01-31	https://www.who.int/emergencies/disease-outbreak-news/item/2005_01_31-en
933	2005-02-02	https://www.who.int/emergencies/disease-outbreak-news/item/2005_02_02-en
934	2005-02-03	https://www.who.int/emergencies/disease-outbreak-news/item/2005_02_03-en
935	2005-02-09	https://www.who.int/emergencies/disease-outbreak-news/item/2005_02_09-en
936	2005-02-10	https://www.who.int/emergencies/disease-outbreak-news/item/2005_02_10-en
937	2005-02-14	https://www.who.int/emergencies/disease-outbreak-news/item/2005_02_14-en
938	2005-02-15	https://www.who.int/emergencies/disease-outbreak-news/item/2005_02_15-en
939	2005-02-18	https://www.who.int/emergencies/disease-outbreak-news/item/2005_02_18-en
940	2005-03-01	https://www.who.int/emergencies/disease-outbreak-news/item/2005_03_01a-en
941	2005-03-04	https://www.who.int/emergencies/disease-outbreak-news/item/2005_03_04-en
942	2005-03-07	https://www.who.int/emergencies/disease-outbreak-news/item/2005_03_07-en
943	2005-03-09	https://www.who.int/emergencies/disease-outbreak-news/item/2005_03_09-en
944	2005-03-11	https://www.who.int/emergencies/disease-outbreak-news/item/2005_03_11-en
945	2005-03-15	https://www.who.int/emergencies/disease-outbreak-news/item/2005_03_15-en
946	2005-03-17	https://www.who.int/emergencies/disease-outbreak-news/item/2005_03_17a-en
947	2005-03-17	https://www.who.int/emergencies/disease-outbreak-news/item/2005_03_17b-en
948	2005-03-23	https://www.who.int/emergencies/disease-outbreak-news/item/2005_03_23a-en
949	2005-03-24	https://www.who.int/emergencies/disease-outbreak-news/item/2005_03_24-en
950	2005-03-24	https://www.who.int/emergencies/disease-outbreak-news/item/2005_03_24a-en
951	2005-03-29	https://www.who.int/emergencies/disease-outbreak-news/item/2005_03_29-en
952	2005-03-29	https://www.who.int/emergencies/disease-outbreak-news/item/2005_03_29a-en
953	2005-03-29	https://www.who.int/emergencies/disease-outbreak-news/item/2005_03_29b-en
954	2005-03-30	https://www.who.int/emergencies/disease-outbreak-news/item/2005_03_30-en
955	2005-03-31	https://www.who.int/emergencies/disease-outbreak-news/item/2005_03_31a-en
956	2005-04-01	https://www.who.int/emergencies/disease-outbreak-news/item/2005_04_01-en
957	2005-04-04	https://www.who.int/emergencies/disease-outbreak-news/item/2005_04_04a-en
958	2005-04-05	https://www.who.int/emergencies/disease-outbreak-news/item/2005_04_05-en
959	2005-04-06	https://www.who.int/emergencies/disease-outbreak-news/item/2005_04_06-en
960	2005-04-06	https://www.who.int/emergencies/disease-outbreak-news/item/2005_04_06a-en
961	2005-04-07	https://www.who.int/emergencies/disease-outbreak-news/item/2005_04_07-en
962	2005-04-08	https://www.who.int/emergencies/disease-outbreak-news/item/2005_04_08a-en
963	2005-04-11	https://www.who.int/emergencies/disease-outbreak-news/item/2005_04_11-en
964	2005-04-12	https://www.who.int/emergencies/disease-outbreak-news/item/2005_04_12-en
965	2005-04-13	https://www.who.int/emergencies/disease-outbreak-news/item/2005_04_13-en
966	2005-04-14	https://www.who.int/emergencies/disease-outbreak-news/item/2005_04_14a-en
967	2005-04-15	https://www.who.int/emergencies/disease-outbreak-news/item/2005_04_15-en

968	2005-04-20	https://www.who.int/emergencies/disease-outbreak-news/item/2005_04_20-en
969	2005-04-21	https://www.who.int/emergencies/disease-outbreak-news/item/2005_04_21-en
970	2005-04-22	https://www.who.int/emergencies/disease-outbreak-news/item/2005_04_22-en
971	2005-04-25	https://www.who.int/emergencies/disease-outbreak-news/item/2005_04_25-en
972	2005-04-29	https://www.who.int/emergencies/disease-outbreak-news/item/2005_04_29-en
973	2005-05-04	https://www.who.int/emergencies/disease-outbreak-news/item/2005_05_04-en
974	2005-05-06	https://www.who.int/emergencies/disease-outbreak-news/item/2005_05_06-en
975	2005-05-09	https://www.who.int/emergencies/disease-outbreak-news/item/2005_05_09-en
976	2005-05-11	https://www.who.int/emergencies/disease-outbreak-news/item/2005_05_11-en
977	2005-05-12	https://www.who.int/emergencies/disease-outbreak-news/item/2005_05_12-en
978	2005-05-13	https://www.who.int/emergencies/disease-outbreak-news/item/2005_05_13a-en
979	2005-05-17	https://www.who.int/emergencies/disease-outbreak-news/item/2005_05_17-en
980	2005-05-18	https://www.who.int/emergencies/disease-outbreak-news/item/2005_05_18a-en
981	2005-05-19	https://www.who.int/emergencies/disease-outbreak-news/item/2005_05_19-en
982	2005-05-25	https://www.who.int/emergencies/disease-outbreak-news/item/2005_05_25-en
983	2005-05-27	https://www.who.int/emergencies/disease-outbreak-news/item/2005_05_27a-en
984	2005-05-30	https://www.who.int/emergencies/disease-outbreak-news/item/2005_05_30a-en
985	2005-06-06	https://www.who.int/emergencies/disease-outbreak-news/item/2005_06_06-en
986	2005-06-07	https://www.who.int/emergencies/disease-outbreak-news/item/2005_06_07-en
987	2005-06-08	https://www.who.int/emergencies/disease-outbreak-news/item/2005_06_08a-en
988	2005-06-09	https://www.who.int/emergencies/disease-outbreak-news/item/2005_06_09-en
989	2005-06-14	https://www.who.int/emergencies/disease-outbreak-news/item/2005_06_14a-en
990	2005-06-14	https://www.who.int/emergencies/disease-outbreak-news/item/2005_06_14b-en
991	2005-06-15	https://www.who.int/emergencies/disease-outbreak-news/item/2005_06_15-en
992	2005-06-16	https://www.who.int/emergencies/disease-outbreak-news/item/2005_06_16a-en
993	2005-06-17	https://www.who.int/emergencies/disease-outbreak-news/item/2005_06_17a-en
994	2005-06-20	https://www.who.int/emergencies/disease-outbreak-news/item/2005_06_20-en
995	2005-06-21	https://www.who.int/emergencies/disease-outbreak-news/item/2005_06_21-en
996	2005-06-27	https://www.who.int/emergencies/disease-outbreak-news/item/2005_06_27-en
997	2005-06-28	https://www.who.int/emergencies/disease-outbreak-news/item/2005_06_28-en
998	2005-06-30	https://www.who.int/emergencies/disease-outbreak-news/item/2005_06_30-en
999	2005-07-01	https://www.who.int/emergencies/disease-outbreak-news/item/2005_07_01a-en
1000	2005-07-04	https://www.who.int/emergencies/disease-outbreak-news/item/2005_07_04-en
1001	2005-07-05	https://www.who.int/emergencies/disease-outbreak-news/item/2005_07_05-en
1002	2005-07-08	https://www.who.int/emergencies/disease-outbreak-news/item/2005_07_08a-en
1003	2005-07-13	https://www.who.int/emergencies/disease-outbreak-news/item/2005_07_13-en
1004	2005-07-15	https://www.who.int/emergencies/disease-outbreak-news/item/2005_07_15-en
1005	2005-07-21	https://www.who.int/emergencies/disease-outbreak-news/item/2005_07_21a-en
1006	2005-07-29	https://www.who.int/emergencies/disease-outbreak-news/item/2005_07_29a-en

1007	2005-07-29	https://www.who.int/emergencies/disease-outbreak-news/item/2005_07_29b-en
1008	2005-07-29	https://www.who.int/emergencies/disease-outbreak-news/item/2005_07_29c-en
1009	2005-08-03	https://www.who.int/emergencies/disease-outbreak-news/item/2005_08_03-en
1010	2005-08-05	https://www.who.int/emergencies/disease-outbreak-news/item/2005_08_05-en
1011	2005-08-17	https://www.who.int/emergencies/disease-outbreak-news/item/2005_08_17-en
1012	2005-08-18	https://www.who.int/emergencies/disease-outbreak-news/item/2005_08_18-en
1013	2005-08-24	https://www.who.int/emergencies/disease-outbreak-news/item/2005_08_24-en
1014	2005-08-25	https://www.who.int/emergencies/disease-outbreak-news/item/2005_08_25-en
1015	2005-08-25	https://www.who.int/emergencies/disease-outbreak-news/item/2005_08_25a-en
1016	2005-08-26	https://www.who.int/emergencies/disease-outbreak-news/item/2005_08_26-en
1017	2005-08-31	https://www.who.int/emergencies/disease-outbreak-news/item/2005_08_31-en
1018	2005-09-06	https://www.who.int/emergencies/disease-outbreak-news/item/2005_09_06-en
1019	2005-09-13	https://www.who.int/emergencies/disease-outbreak-news/item/2005_09_13a-en
1020	2005-09-16	https://www.who.int/emergencies/disease-outbreak-news/item/2005_09_16-en
1021	2005-09-19	https://www.who.int/emergencies/disease-outbreak-news/item/2005_09_19-en
1022	2005-09-22	https://www.who.int/emergencies/disease-outbreak-news/item/2005_09_22a-en
1023	2005-09-23	https://www.who.int/emergencies/disease-outbreak-news/item/2005_09_23-en
1024	2005-09-29	https://www.who.int/emergencies/disease-outbreak-news/item/2005_09_29-en
1025	2005-09-30	https://www.who.int/emergencies/disease-outbreak-news/item/2005_09_30-en
1026	2005-10-10	https://www.who.int/emergencies/disease-outbreak-news/item/2005_10_10-en
1027	2005-10-13	https://www.who.int/emergencies/disease-outbreak-news/item/2005_10_13-en
1028	2005-10-20	https://www.who.int/emergencies/disease-outbreak-news/item/2005_10_20a-en
1029	2005-10-24	https://www.who.int/emergencies/disease-outbreak-news/item/2005_10_24-en
1030	2005-11-01	https://www.who.int/emergencies/disease-outbreak-news/item/2005_11_01-en
1031	2005-11-02	https://www.who.int/emergencies/disease-outbreak-news/item/2005_11_02-en
1032	2005-11-03	https://www.who.int/emergencies/disease-outbreak-news/item/2005_11_03-en
1033	2005-11-07	https://www.who.int/emergencies/disease-outbreak-news/item/2005_11_07-en
1034	2005-11-07	https://www.who.int/emergencies/disease-outbreak-news/item/2005_11_07a-en
1035	2005-11-09	https://www.who.int/emergencies/disease-outbreak-news/item/2005_11_09-en
1036	2005-11-10	https://www.who.int/emergencies/disease-outbreak-news/item/2005_11_10-en
1037	2005-11-14	https://www.who.int/emergencies/disease-outbreak-news/item/2005_11_14-en
1038	2005-11-16	https://www.who.int/emergencies/disease-outbreak-news/item/2005_11_16-en
1039	2005-11-17	https://www.who.int/emergencies/disease-outbreak-news/item/2005_11_17-en
1040	2005-11-21	https://www.who.int/emergencies/disease-outbreak-news/item/2005_11_21-en
1041	2005-11-24	https://www.who.int/emergencies/disease-outbreak-news/item/2005_11_24-en
1042	2005-11-25	https://www.who.int/emergencies/disease-outbreak-news/item/2005_11_25-en
1043	2005-11-28	https://www.who.int/emergencies/disease-outbreak-news/item/2005_11_28a-en
1044	2005-11-29	https://www.who.int/emergencies/disease-outbreak-news/item/2005_11_29-en
1045	2005-12-06	https://www.who.int/emergencies/disease-outbreak-news/item/2005_12_06-en

1046	2005-12-07	https://www.who.int/emergencies/disease-outbreak-news/item/2005_12_07a-en
1047	2005-12-09	https://www.who.int/emergencies/disease-outbreak-news/item/2005_12_09a-en
1048	2005-12-09	https://www.who.int/emergencies/disease-outbreak-news/item/2005_12_09-en
1049	2005-12-14	https://www.who.int/emergencies/disease-outbreak-news/item/2005_12_14-en
1050	2005-12-16	https://www.who.int/emergencies/disease-outbreak-news/item/2005_12_16-en
1051	2005-12-19	https://www.who.int/emergencies/disease-outbreak-news/item/2005_12_19-en
1052	2005-12-23	https://www.who.int/emergencies/disease-outbreak-news/item/2005_12_23-en
1053	2005-12-30	https://www.who.int/emergencies/disease-outbreak-news/item/2005_12_30-en
1054	2006-01-05	https://www.who.int/emergencies/disease-outbreak-news/item/2006_01_05-en
1055	2006-01-07	https://www.who.int/emergencies/disease-outbreak-news/item/2006_01_07-en
1056	2006-01-09	https://www.who.int/emergencies/disease-outbreak-news/item/2006_01_09-en
1057	2006-01-10	https://www.who.int/emergencies/disease-outbreak-news/item/2006_01_10a-en
1058	2006-01-12	https://www.who.int/emergencies/disease-outbreak-news/item/2006_01_12-en
1059	2006-01-14	https://www.who.int/emergencies/disease-outbreak-news/item/2006_01_14-en
1060	2006-01-16	https://www.who.int/emergencies/disease-outbreak-news/item/2006_01_16-en
1061	2006-01-18	https://www.who.int/emergencies/disease-outbreak-news/item/2006_01_18-en
1062	2006-01-19	https://www.who.int/emergencies/disease-outbreak-news/item/2006_01_19-en
1063	2006-01-23	https://www.who.int/emergencies/disease-outbreak-news/item/2006_01_23-en
1064	2006-01-25	https://www.who.int/emergencies/disease-outbreak-news/item/2006_01_25a-en
1065	2006-01-30	https://www.who.int/emergencies/disease-outbreak-news/item/2006_01_30-en
1066	2006-01-30	https://www.who.int/emergencies/disease-outbreak-news/item/2006_01_30a-en
1067	2006-02-02	https://www.who.int/emergencies/disease-outbreak-news/item/2006_02_02-en
1068	2006-02-03	https://www.who.int/emergencies/disease-outbreak-news/item/2006_02_03-en
1069	2006-02-06	https://www.who.int/emergencies/disease-outbreak-news/item/2006_02_06-en
1070	2006-02-07	https://www.who.int/emergencies/disease-outbreak-news/item/2006_02_07-en
1071	2006-02-08	https://www.who.int/emergencies/disease-outbreak-news/item/2006_02_08-en
1072	2006-02-09	https://www.who.int/emergencies/disease-outbreak-news/item/2006_02_09-en
1073	2006-02-10	https://www.who.int/emergencies/disease-outbreak-news/item/2006_02_10-en
1074	2006-02-13	https://www.who.int/emergencies/disease-outbreak-news/item/2006_02_13a-en
1075	2006-02-17	https://www.who.int/emergencies/disease-outbreak-news/item/2006_02_17-en
1076	2006-02-17	https://www.who.int/emergencies/disease-outbreak-news/item/2006_02_17a-en
1077	2006-02-20	https://www.who.int/emergencies/disease-outbreak-news/item/2006_02_20-en
1078	2006-02-21	https://www.who.int/emergencies/disease-outbreak-news/item/2006_02_21-en
1079	2006-02-21	https://www.who.int/emergencies/disease-outbreak-news/item/2006_02_21b-en
1080	2006-02-22	https://www.who.int/emergencies/disease-outbreak-news/item/2006_02_22-en
1081	2006-02-23	https://www.who.int/emergencies/disease-outbreak-news/item/2006_02_23-en
1082	2006-02-24	https://www.who.int/emergencies/disease-outbreak-news/item/2006_02_24-en
1083	2006-02-27	https://www.who.int/emergencies/disease-outbreak-news/item/2006_02_27a-en
1084	2006-02-28	https://www.who.int/emergencies/disease-outbreak-news/item/2006_02_28a-en

1085	2006-03-01	https://www.who.int/emergencies/disease-outbreak-news/item/2006_03_01a-en
1086	2006-03-03	https://www.who.int/emergencies/disease-outbreak-news/item/2006_03_03a-en
1087	2006-03-06	https://www.who.int/emergencies/disease-outbreak-news/item/2006_03_06a-en
1088	2006-03-08	https://www.who.int/emergencies/disease-outbreak-news/item/2006_03_08-en
1089	2006-03-09	https://www.who.int/emergencies/disease-outbreak-news/item/2006_03_09a-en
1090	2006-03-10	https://www.who.int/emergencies/disease-outbreak-news/item/2006_03_10a-en
1091	2006-03-13	https://www.who.int/emergencies/disease-outbreak-news/item/2006_03_13-en
1092	2006-03-14	https://www.who.int/emergencies/disease-outbreak-news/item/2006_03_14-en
1093	2006-03-16	https://www.who.int/emergencies/disease-outbreak-news/item/2006_03_16-en
1094	2006-03-17	https://www.who.int/emergencies/disease-outbreak-news/item/2006_03_17-en
1095	2006-03-20	https://www.who.int/emergencies/disease-outbreak-news/item/2006_03_20-en
1096	2006-03-21	https://www.who.int/emergencies/disease-outbreak-news/item/2006_03_21-en
1097	2006-03-21	https://www.who.int/emergencies/disease-outbreak-news/item/2006_03_21a-en
1098	2006-03-22	https://www.who.int/emergencies/disease-outbreak-news/item/2006_03_22a-en
1099	2006-03-24	https://www.who.int/emergencies/disease-outbreak-news/item/2006_03_24a-en
1100	2006-03-24	https://www.who.int/emergencies/disease-outbreak-news/item/2006_03_24c-en
1101	2006-03-29	https://www.who.int/emergencies/disease-outbreak-news/item/2006_03_29-en
1102	2006-04-03	https://www.who.int/emergencies/disease-outbreak-news/item/2006_04_03-en
1103	2006-04-04	https://www.who.int/emergencies/disease-outbreak-news/item/2006_04_04-en
1104	2006-04-06	https://www.who.int/emergencies/disease-outbreak-news/item/2006_04_06a-en
1105	2006-04-11	https://www.who.int/emergencies/disease-outbreak-news/item/2006_04_11-en
1106	2006-04-12	https://www.who.int/emergencies/disease-outbreak-news/item/2006_04_12-en
1107	2006-04-13	https://www.who.int/emergencies/disease-outbreak-news/item/2006_04_13-en
1108	2006-04-19	https://www.who.int/emergencies/disease-outbreak-news/item/2006_04_19-en
1109	2006-04-19	https://www.who.int/emergencies/disease-outbreak-news/item/2006_04_19a-en
1110	2006-04-21	https://www.who.int/emergencies/disease-outbreak-news/item/2006_04_21a-en
1111	2006-04-27	https://www.who.int/emergencies/disease-outbreak-news/item/2006_04_27-en
1112	2006-05-04	https://www.who.int/emergencies/disease-outbreak-news/item/2006_05_04-en
1113	2006-05-05	https://www.who.int/emergencies/disease-outbreak-news/item/2006_05_05-en
1114	2006-05-08	https://www.who.int/emergencies/disease-outbreak-news/item/2006_05_08-en
1115	2006-05-10	https://www.who.int/emergencies/disease-outbreak-news/item/2006_05_10-en
1116	2006-05-12	https://www.who.int/emergencies/disease-outbreak-news/item/2006_05_12-en
1117	2006-05-18	https://www.who.int/emergencies/disease-outbreak-news/item/2006_05_18a-en
1118	2006-05-18	https://www.who.int/emergencies/disease-outbreak-news/item/2006_05_18b-en
1119	2006-05-18	https://www.who.int/emergencies/disease-outbreak-news/item/2006_05_18c-en
1120	2006-05-19	https://www.who.int/emergencies/disease-outbreak-news/item/2006_05_19-en
1121	2006-05-22	https://www.who.int/emergencies/disease-outbreak-news/item/2006_05_22-en
1122	2006-05-23	https://www.who.int/emergencies/disease-outbreak-news/item/2006_05_23-en
1123	2006-05-25	https://www.who.int/emergencies/disease-outbreak-news/item/2006_05_25-en

1124	2006-05-29	https://www.who.int/emergencies/disease-outbreak-news/item/2006_05_29-en
1125	2006-05-31	https://www.who.int/emergencies/disease-outbreak-news/item/2006_05_31-en
1126	2006-06-06	https://www.who.int/emergencies/disease-outbreak-news/item/2006_06_06a-en
1127	2006-06-06	https://www.who.int/emergencies/disease-outbreak-news/item/2006_06_06b-en
1128	2006-06-07	https://www.who.int/emergencies/disease-outbreak-news/item/2006_06_07-en
1129	2006-06-09	https://www.who.int/emergencies/disease-outbreak-news/item/2006_06_09-en
1130	2006-06-14	https://www.who.int/emergencies/disease-outbreak-news/item/2006_06_14-en
1131	2006-06-15	https://www.who.int/emergencies/disease-outbreak-news/item/2006_06_15-en
1132	2006-06-16	https://www.who.int/emergencies/disease-outbreak-news/item/2006_06_16-en
1133	2006-06-20	https://www.who.int/emergencies/disease-outbreak-news/item/2006_06_20-en
1134	2006-06-21	https://www.who.int/emergencies/disease-outbreak-news/item/2006_06_21a-en
1135	2006-06-30	https://www.who.int/emergencies/disease-outbreak-news/item/2006_06_30-en
1136	2006-07-04	https://www.who.int/emergencies/disease-outbreak-news/item/2006_07_04-en
1137	2006-07-14	https://www.who.int/emergencies/disease-outbreak-news/item/2006_07_14-en
1138	2006-07-20	https://www.who.int/emergencies/disease-outbreak-news/item/2006_07_20-en
1139	2006-07-25	https://www.who.int/emergencies/disease-outbreak-news/item/2006_07_25-en
1140	2006-07-26	https://www.who.int/emergencies/disease-outbreak-news/item/2006_07_26-en
1141	2006-08-07	https://www.who.int/emergencies/disease-outbreak-news/item/2006_08_07-en
1142	2006-08-08	https://www.who.int/emergencies/disease-outbreak-news/item/2006_08_08a-en
1143	2006-08-08	https://www.who.int/emergencies/disease-outbreak-news/item/2006_08_08b-en
1144	2006-08-08	https://www.who.int/emergencies/disease-outbreak-news/item/2006_08_08-en
1145	2006-08-09	https://www.who.int/emergencies/disease-outbreak-news/item/2006_08_09-en
1146	2006-08-14	https://www.who.int/emergencies/disease-outbreak-news/item/2006_08_14-en
1147	2006-08-14	https://www.who.int/emergencies/disease-outbreak-news/item/2006_08_14a-en
1148	2006-08-17	https://www.who.int/emergencies/disease-outbreak-news/item/2006_08_17-en
1149	2006-08-21	https://www.who.int/emergencies/disease-outbreak-news/item/2006_08_21-en
1150	2006-08-23	https://www.who.int/emergencies/disease-outbreak-news/item/2006_08_23-en
1151	2006-09-08	https://www.who.int/emergencies/disease-outbreak-news/item/2006_09_08a-en
1152	2006-09-14	https://www.who.int/emergencies/disease-outbreak-news/item/2006_09_14-en
1153	2006-09-19	https://www.who.int/emergencies/disease-outbreak-news/item/2006_09_19-en
1154	2006-09-25	https://www.who.int/emergencies/disease-outbreak-news/item/2006_09_25-en
1155	2006-09-27	https://www.who.int/emergencies/disease-outbreak-news/item/2006_09_27a-en
1156	2006-09-28	https://www.who.int/emergencies/disease-outbreak-news/item/2006_09_28-en
1157	2006-10-03	https://www.who.int/emergencies/disease-outbreak-news/item/2006_10_03-en
1158	2006-10-04	https://www.who.int/emergencies/disease-outbreak-news/item/2006_10_04-en
1159	2006-10-11	https://www.who.int/emergencies/disease-outbreak-news/item/2006_10_11-en
1160	2006-10-11	https://www.who.int/emergencies/disease-outbreak-news/item/2006_10_11a-en
1161	2006-10-13	https://www.who.int/emergencies/disease-outbreak-news/item/2006_10_13-en
1162	2006-10-16	https://www.who.int/emergencies/disease-outbreak-news/item/2006_10_16-en

1163	2006-10-17	https://www.who.int/emergencies/disease-outbreak-news/item/2006_10_17-en
1164	2006-10-19	https://www.who.int/emergencies/disease-outbreak-news/item/2006_10_19a-en
1165	2006-10-31	https://www.who.int/emergencies/disease-outbreak-news/item/2006_10_31-en
1166	2006-11-07	https://www.who.int/emergencies/disease-outbreak-news/item/2006_11_07-en
1167	2006-11-13	https://www.who.int/emergencies/disease-outbreak-news/item/2006_11_13-en
1168	2006-11-21	https://www.who.int/emergencies/disease-outbreak-news/item/2006_11_21-en
1169	2006-11-29	https://www.who.int/emergencies/disease-outbreak-news/item/2006_11_29-en
1170	2006-12-19	https://www.who.int/emergencies/disease-outbreak-news/item/2006_12_19-en
1171	2006-12-26	https://www.who.int/emergencies/disease-outbreak-news/item/2006_12_26-en
1172	2006-12-27	https://www.who.int/emergencies/disease-outbreak-news/item/2006_12_27a-en
1173	2007-01-09	https://www.who.int/emergencies/disease-outbreak-news/item/2007_01_09-en
1174	2007-01-10	https://www.who.int/emergencies/disease-outbreak-news/item/2007_01_10-en
1175	2007-01-11	https://www.who.int/emergencies/disease-outbreak-news/item/2007_01_11-en
1176	2007-01-11	https://www.who.int/emergencies/disease-outbreak-news/item/2007_01_11a-en
1177	2007-01-12	https://www.who.int/emergencies/disease-outbreak-news/item/2007_01_12-en
1178	2007-01-15	https://www.who.int/emergencies/disease-outbreak-news/item/2007_01_15a-en
1179	2007-01-18	https://www.who.int/emergencies/disease-outbreak-news/item/2007_01_18-en
1180	2007-01-22	https://www.who.int/emergencies/disease-outbreak-news/item/2007_01_22-en
1181	2007-01-22	https://www.who.int/emergencies/disease-outbreak-news/item/2007_01_22a-en
1182	2007-01-24	https://www.who.int/emergencies/disease-outbreak-news/item/2007_01_24-en
1183	2007-01-29	https://www.who.int/emergencies/disease-outbreak-news/item/2007_01_29-en
1184	2007-01-29	https://www.who.int/emergencies/disease-outbreak-news/item/2007_01_29a-en
1185	2007-01-31	https://www.who.int/emergencies/disease-outbreak-news/item/2007_01_31a-en
1186	2007-02-02	https://www.who.int/emergencies/disease-outbreak-news/item/2007_02_02-en
1187	2007-02-03	https://www.who.int/emergencies/disease-outbreak-news/item/2007_02_03-en
1188	2007-02-06	https://www.who.int/emergencies/disease-outbreak-news/item/2007_02_06-en
1189	2007-02-07	https://www.who.int/emergencies/disease-outbreak-news/item/2007_02_07a-en
1190	2007-02-09	https://www.who.int/emergencies/disease-outbreak-news/item/2007_02_09-en
1191	2007-02-12	https://www.who.int/emergencies/disease-outbreak-news/item/2007_02_12-en
1192	2007-02-15	https://www.who.int/emergencies/disease-outbreak-news/item/2007_02_15-en
1193	2007-02-15	https://www.who.int/emergencies/disease-outbreak-news/item/2007_2_15-en
1194	2007-02-16	https://www.who.int/emergencies/disease-outbreak-news/item/2007_02_16-en
1195	2007-02-19	https://www.who.int/emergencies/disease-outbreak-news/item/2007_02_19-en
1196	2007-02-27	https://www.who.int/emergencies/disease-outbreak-news/item/2007_02_27-en
1197	2007-03-01	https://www.who.int/emergencies/disease-outbreak-news/item/2007_03_01a-en
1198	2007-03-08	https://www.who.int/emergencies/disease-outbreak-news/item/2007_03_08-en
1199	2007-03-12	https://www.who.int/emergencies/disease-outbreak-news/item/2007_03_12-en
1200	2007-03-15	https://www.who.int/emergencies/disease-outbreak-news/item/2007_03_15-en
1201	2007-03-15	https://www.who.int/emergencies/disease-outbreak-news/item/2007_3_15a-en

1202	2007-03-16	https://www.who.int/emergencies/disease-outbreak-news/item/2007_03_16-en
1203	2007-03-19	https://www.who.int/emergencies/disease-outbreak-news/item/2007_03_19a-en
1204	2007-03-20	https://www.who.int/emergencies/disease-outbreak-news/item/2007_03_20-en
1205	2007-03-23	https://www.who.int/emergencies/disease-outbreak-news/item/2007_03_23-en
1206	2007-03-27	https://www.who.int/emergencies/disease-outbreak-news/item/2007_03_27-en
1207	2007-03-28	https://www.who.int/emergencies/disease-outbreak-news/item/2007_03_28-en
1208	2007-03-29	https://www.who.int/emergencies/disease-outbreak-news/item/2007_03_29-en
1209	2007-04-02	https://www.who.int/emergencies/disease-outbreak-news/item/2007_04_02-en
1210	2007-04-10	https://www.who.int/emergencies/disease-outbreak-news/item/2007_04_10a-en
1211	2007-04-11	https://www.who.int/emergencies/disease-outbreak-news/item/2007_04_11-en
1212	2007-04-17	https://www.who.int/emergencies/disease-outbreak-news/item/2007_04_17-en
1213	2007-05-09	https://www.who.int/emergencies/disease-outbreak-news/item/2007_05_09-en
1214	2007-05-16	https://www.who.int/emergencies/disease-outbreak-news/item/2007_05_16-en
1215	2007-05-24	https://www.who.int/emergencies/disease-outbreak-news/item/2007_05_24-en
1216	2007-05-29	https://www.who.int/emergencies/disease-outbreak-news/item/2007_05_29-en
1217	2007-05-30	https://www.who.int/emergencies/disease-outbreak-news/item/2007_05_30a-en
1218	2007-05-31	https://www.who.int/emergencies/disease-outbreak-news/item/2007_05_31-en
1219	2007-06-04	https://www.who.int/emergencies/disease-outbreak-news/item/2007_06_04a-en
1220	2007-06-06	https://www.who.int/emergencies/disease-outbreak-news/item/2007_06_06-en
1221	2007-06-11	https://www.who.int/emergencies/disease-outbreak-news/item/2007_06_11-en
1222	2007-06-12	https://www.who.int/emergencies/disease-outbreak-news/item/2007_06_12-en
1223	2007-06-15	https://www.who.int/emergencies/disease-outbreak-news/item/2007_06_15-en
1224	2007-06-25	https://www.who.int/emergencies/disease-outbreak-news/item/2007_06_25a-en
1225	2007-06-29	https://www.who.int/emergencies/disease-outbreak-news/item/2007_06_29-en
1226	2007-07-11	https://www.who.int/emergencies/disease-outbreak-news/item/2007_07_11-en
1227	2007-07-25	https://www.who.int/emergencies/disease-outbreak-news/item/2007_07_25-en
1228	2007-07-30	https://www.who.int/emergencies/disease-outbreak-news/item/2007_07_30-en
1229	2007-08-03	https://www.who.int/emergencies/disease-outbreak-news/item/2007_08_03-en
1230	2007-08-09	https://www.who.int/emergencies/disease-outbreak-news/item/2007_08_09-en
1231	2007-08-14	https://www.who.int/emergencies/disease-outbreak-news/item/2007_08_14a-en
1232	2007-08-14	https://www.who.int/emergencies/disease-outbreak-news/item/2007_08_14b-en
1233	2007-08-16	https://www.who.int/emergencies/disease-outbreak-news/item/2007_08_16-en
1234	2007-08-21	https://www.who.int/emergencies/disease-outbreak-news/item/2007_08_21-en
1235	2007-08-23	https://www.who.int/emergencies/disease-outbreak-news/item/2007_08_23-en
1236	2007-08-31	https://www.who.int/emergencies/disease-outbreak-news/item/2007_08_31a-en
1237	2007-09-10	https://www.who.int/emergencies/disease-outbreak-news/item/2007_09_10a-en
1238	2007-09-11	https://www.who.int/emergencies/disease-outbreak-news/item/2007_09_11-en
1239	2007-09-13	https://www.who.int/emergencies/disease-outbreak-news/item/2007_09_13-en
1240	2007-09-14	https://www.who.int/emergencies/disease-outbreak-news/item/2007_09_14-en

1241	2007-09-20	https://www.who.int/emergencies/disease-outbreak-news/item/2007_09_20-en
1242	2007-09-25	https://www.who.int/emergencies/disease-outbreak-news/item/2007_09_25-en
1243	2007-09-25	https://www.who.int/emergencies/disease-outbreak-news/item/testpicturenov07-en
1244	2007-09-27	https://www.who.int/emergencies/disease-outbreak-news/item/2007_09_27-en
1245	2007-10-02	https://www.who.int/emergencies/disease-outbreak-news/item/2007_10_02-en
1246	2007-10-03	https://www.who.int/emergencies/disease-outbreak-news/item/2007_10_03-en
1247	2007-10-03	https://www.who.int/emergencies/disease-outbreak-news/item/2007_10_03a-en
1248	2007-10-08	https://www.who.int/emergencies/disease-outbreak-news/item/2007_10_08-en
1249	2007-10-12	https://www.who.int/emergencies/disease-outbreak-news/item/2007_10_12-en
1250	2007-10-17	https://www.who.int/emergencies/disease-outbreak-news/item/2007_10_17-en
1251	2007-10-25	https://www.who.int/emergencies/disease-outbreak-news/item/2007_10_25-en
1252	2007-10-31	https://www.who.int/emergencies/disease-outbreak-news/item/2007_10_31-en
1253	2007-11-05	https://www.who.int/emergencies/disease-outbreak-news/item/2007_11_05a-en
1254	2007-11-07	https://www.who.int/emergencies/disease-outbreak-news/item/2007_11_07-en
1255	2007-11-12	https://www.who.int/emergencies/disease-outbreak-news/item/2007_11_12-en
1256	2007-11-14	https://www.who.int/emergencies/disease-outbreak-news/item/2007_11_14-en
1257	2007-11-16	https://www.who.int/emergencies/disease-outbreak-news/item/2007_11_16-en
1258	2007-11-21	https://www.who.int/emergencies/disease-outbreak-news/item/2007_11_21-en
1259	2007-11-22	https://www.who.int/emergencies/disease-outbreak-news/item/2007_11_22-en
1260	2007-11-30	https://www.who.int/emergencies/disease-outbreak-news/item/2007_11_30a-en
1261	2007-12-04	https://www.who.int/emergencies/disease-outbreak-news/item/2007_12_04-en
1262	2007-12-07	https://www.who.int/emergencies/disease-outbreak-news/item/2007_12_07-en
1263	2007-12-09	https://www.who.int/emergencies/disease-outbreak-news/item/2007_12_09-en
1264	2007-12-12	https://www.who.int/emergencies/disease-outbreak-news/item/2007_12_12-en
1265	2007-12-13	https://www.who.int/emergencies/disease-outbreak-news/item/2007_12_13-en
1266	2007-12-14	https://www.who.int/emergencies/disease-outbreak-news/item/2007_12_14-en
1267	2007-12-15	https://www.who.int/emergencies/disease-outbreak-news/item/2007_12_15-en
1268	2007-12-18	https://www.who.int/emergencies/disease-outbreak-news/item/2007_12_18-en
1269	2007-12-21	https://www.who.int/emergencies/disease-outbreak-news/item/2007_12_21-en
1270	2007-12-26	https://www.who.int/emergencies/disease-outbreak-news/item/2007_12_26-en
1271	2007-12-26	https://www.who.int/emergencies/disease-outbreak-news/item/2007_12_26a-en
1272	2007-12-27	https://www.who.int/emergencies/disease-outbreak-news/item/2007_12_27-en
1273	2007-12-28	https://www.who.int/emergencies/disease-outbreak-news/item/2007_12_28a-en
1274	2008-01-02	https://www.who.int/emergencies/disease-outbreak-news/item/2008_01_02-en
1275	2008-01-03	https://www.who.int/emergencies/disease-outbreak-news/item/2008_01_03-en
1276	2008-01-11	https://www.who.int/emergencies/disease-outbreak-news/item/2008_01_11-en
1277	2008-01-15	https://www.who.int/emergencies/disease-outbreak-news/item/2008_01_15-en
1278	2008-01-18	https://www.who.int/emergencies/disease-outbreak-news/item/2008_01_18-en
1279	2008-01-21	https://www.who.int/emergencies/disease-outbreak-news/item/2008_01_21-en

1280	2008-01-22	https://www.who.int/emergencies/disease-outbreak-news/item/2008_01_22-en
1281	2008-01-23	https://www.who.int/emergencies/disease-outbreak-news/item/2008_01_23-en
1282	2008-01-24	https://www.who.int/emergencies/disease-outbreak-news/item/2008_01_24a-en
1283	2008-01-29	https://www.who.int/emergencies/disease-outbreak-news/item/2008_01_29-en
1284	2008-01-30	https://www.who.int/emergencies/disease-outbreak-news/item/2008_01_30-en
1285	2008-02-01	https://www.who.int/emergencies/disease-outbreak-news/item/2008_02_01-en
1286	2008-02-05	https://www.who.int/emergencies/disease-outbreak-news/item/2008_02_05-en
1287	2008-02-07	https://www.who.int/emergencies/disease-outbreak-news/item/2008_02_07-en
1288	2008-02-12	https://www.who.int/emergencies/disease-outbreak-news/item/2008_02_12-en
1289	2008-02-15	https://www.who.int/emergencies/disease-outbreak-news/item/2008_02_15-en
1290	2008-02-20	https://www.who.int/emergencies/disease-outbreak-news/item/2007_02_20b-en
1291	2008-02-20	https://www.who.int/emergencies/disease-outbreak-news/item/2008_02_20-en
1292	2008-02-20	https://www.who.int/emergencies/disease-outbreak-news/item/2008_02_20a-en
1293	2008-02-21	https://www.who.int/emergencies/disease-outbreak-news/item/2008_02_21a-en
1294	2008-02-22	https://www.who.int/emergencies/disease-outbreak-news/item/2008_02_22a-en
1295	2008-02-26	https://www.who.int/emergencies/disease-outbreak-news/item/2008_02_26b-en
1296	2008-02-28	https://www.who.int/emergencies/disease-outbreak-news/item/2008_02_28-en
1297	2008-02-28	https://www.who.int/emergencies/disease-outbreak-news/item/2008_02_28a-en
1298	2008-03-04	https://www.who.int/emergencies/disease-outbreak-news/item/2008_03_04-en
1299	2008-03-05	https://www.who.int/emergencies/disease-outbreak-news/item/2008_03_05-en
1300	2008-03-07	https://www.who.int/emergencies/disease-outbreak-news/item/2008_03_07-en
1301	2008-03-11	https://www.who.int/emergencies/disease-outbreak-news/item/2008_03_11-en
1302	2008-03-18	https://www.who.int/emergencies/disease-outbreak-news/item/2008_03_18-en
1303	2008-04-02	https://www.who.int/emergencies/disease-outbreak-news/item/2008_04_02-en
1304	2008-04-03	https://www.who.int/emergencies/disease-outbreak-news/item/2008_04_03-en
1305	2008-04-08	https://www.who.int/emergencies/disease-outbreak-news/item/2008_04_08-en
1306	2008-04-10	https://www.who.int/emergencies/disease-outbreak-news/item/2008_04_10-en
1307	2008-04-15	https://www.who.int/emergencies/disease-outbreak-news/item/2008_04_15-en
1308	2008-04-17	https://www.who.int/emergencies/disease-outbreak-news/item/2008_04_17-en
1309	2008-04-18	https://www.who.int/emergencies/disease-outbreak-news/item/2008_04_18a-en
1310	2008-04-22	https://www.who.int/emergencies/disease-outbreak-news/item/2008_04_22-en
1311	2008-04-25	https://www.who.int/emergencies/disease-outbreak-news/item/2008_04_25-en
1312	2008-04-30	https://www.who.int/emergencies/disease-outbreak-news/item/2008_04_30-en
1313	2008-05-01	https://www.who.int/emergencies/disease-outbreak-news/item/2008_05_01-en
1314	2008-05-07	https://www.who.int/emergencies/disease-outbreak-news/item/2008_05_07-en
1315	2008-05-20	https://www.who.int/emergencies/disease-outbreak-news/item/2008_05_20-en
1316	2008-05-21	https://www.who.int/emergencies/disease-outbreak-news/item/2008_05_21-en
1317	2008-05-28	https://www.who.int/emergencies/disease-outbreak-news/item/2008_05_28-en
1318	2008-06-18	https://www.who.int/emergencies/disease-outbreak-news/item/2008_06_18-en

1319	2008-06-19	https://www.who.int/emergencies/disease-outbreak-news/item/2008_06_19-en
1320	2008-06-23	https://www.who.int/emergencies/disease-outbreak-news/item/2008_06_23-en
1321	2008-07-10	https://www.who.int/emergencies/disease-outbreak-news/item/2008_07_10-en
1322	2008-08-08	https://www.who.int/emergencies/disease-outbreak-news/item/2008_08_08-en
1323	2008-09-10	https://www.who.int/emergencies/disease-outbreak-news/item/2008_09_10a-en
1324	2008-09-18	https://www.who.int/emergencies/disease-outbreak-news/item/2008_09_19-en
1325	2008-09-22	https://www.who.int/emergencies/disease-outbreak-news/item/2008_09_22-en
1326	2008-09-24	https://www.who.int/emergencies/disease-outbreak-news/item/2008_09_24-en
1327	2008-09-29	https://www.who.int/emergencies/disease-outbreak-news/item/2008_09_29a-en
1328	2008-09-29	https://www.who.int/emergencies/disease-outbreak-news/item/2008_09_29c-en
1329	2008-10-10	https://www.who.int/emergencies/disease-outbreak-news/item/2008_10_10-en
1330	2008-10-13	https://www.who.int/emergencies/disease-outbreak-news/item/2008_10_13-en
1331	2008-10-30	https://www.who.int/emergencies/disease-outbreak-news/item/2008_10_30-en
1332	2008-11-03	https://www.who.int/emergencies/disease-outbreak-news/item/2008_11_03-en
1333	2008-12-02	https://www.who.int/emergencies/disease-outbreak-news/item/2008_12_02-en
1334	2008-12-09	https://www.who.int/emergencies/disease-outbreak-news/item/2008_12_09-en
1335	2008-12-12	https://www.who.int/emergencies/disease-outbreak-news/item/2008_12_12-en
1336	2008-12-16	https://www.who.int/emergencies/disease-outbreak-news/item/2008_12_16-en
1337	2008-12-26	https://www.who.int/emergencies/disease-outbreak-news/item/2008_12_26-en
1338	2008-12-26	https://www.who.int/emergencies/disease-outbreak-news/item/2008_12_26a-en
1339	2009-01-02	https://www.who.int/emergencies/disease-outbreak-news/item/2009_01_02-en
1340	2009-01-06	https://www.who.int/emergencies/disease-outbreak-news/item/2009_01_06-en
1341	2009-01-06	https://www.who.int/emergencies/disease-outbreak-news/item/2009_01_06a-en
1342	2009-01-07	https://www.who.int/emergencies/disease-outbreak-news/item/2009_01_07a-en
1343	2009-01-11	https://www.who.int/emergencies/disease-outbreak-news/item/2009_01_11-en
1344	2009-01-14	https://www.who.int/emergencies/disease-outbreak-news/item/2009_01_14a-en
1345	2009-01-19	https://www.who.int/emergencies/disease-outbreak-news/item/2009_01_19-en
1346	2009-01-22	https://www.who.int/emergencies/disease-outbreak-news/item/2009_01_22-en
1347	2009-01-24	https://www.who.int/emergencies/disease-outbreak-news/item/2009_01_24-en
1348	2009-01-26	https://www.who.int/emergencies/disease-outbreak-news/item/2009_01_26-en
1349	2009-01-27	https://www.who.int/emergencies/disease-outbreak-news/item/2009_01_27-en
1350	2009-01-30	https://www.who.int/emergencies/disease-outbreak-news/item/2009_01_30-en
1351	2009-02-02	https://www.who.int/emergencies/disease-outbreak-news/item/2009_02_02-en
1352	2009-02-03	https://www.who.int/emergencies/disease-outbreak-news/item/2009_02_03-en
1353	2009-02-05	https://www.who.int/emergencies/disease-outbreak-news/item/2009_02_05-en
1354	2009-02-09	https://www.who.int/emergencies/disease-outbreak-news/item/2009_02_09-en
1355	2009-02-11	https://www.who.int/emergencies/disease-outbreak-news/item/2009_02_11-en
1356	2009-02-17	https://www.who.int/emergencies/disease-outbreak-news/item/2009_02_17-en
1357	2009-02-18	https://www.who.int/emergencies/disease-outbreak-news/item/2009_02_18-en

1358	2009-02-19	https://www.who.int/emergencies/disease-outbreak-news/item/2009_02_19-en
1359	2009-02-20	https://www.who.int/emergencies/disease-outbreak-news/item/2009_02_20-en
1360	2009-02-24	https://www.who.int/emergencies/disease-outbreak-news/item/2009_02_24-en
1361	2009-02-27	https://www.who.int/emergencies/disease-outbreak-news/item/2009_02_27-en
1362	2009-03-02	https://www.who.int/emergencies/disease-outbreak-news/item/2009_03_02-en
1363	2009-03-02	https://www.who.int/emergencies/disease-outbreak-news/item/2009_03_02a-en
1364	2009-03-04	https://www.who.int/emergencies/disease-outbreak-news/item/2009_03_04-en
1365	2009-03-10	https://www.who.int/emergencies/disease-outbreak-news/item/2009_03_10-en
1366	2009-03-11	https://www.who.int/emergencies/disease-outbreak-news/item/2009_03_11-en
1367	2009-03-23	https://www.who.int/emergencies/disease-outbreak-news/item/2009_03_23a-en
1368	2009-03-25	https://www.who.int/emergencies/disease-outbreak-news/item/2009_03_25-en
1369	2009-03-30	https://www.who.int/emergencies/disease-outbreak-news/item/2009_03_30-en
1370	2009-03-31	https://www.who.int/emergencies/disease-outbreak-news/item/2009_03_31-en
1371	2009-04-08	https://www.who.int/emergencies/disease-outbreak-news/item/2009_04_08a-en
1372	2009-04-17	https://www.who.int/emergencies/disease-outbreak-news/item/2009_04_17-en
1373	2009-04-21	https://www.who.int/emergencies/disease-outbreak-news/item/2009_04_21-en
1374	2009-04-23	https://www.who.int/emergencies/disease-outbreak-news/item/2009_04_23-en
1375	2009-04-23	https://www.who.int/emergencies/disease-outbreak-news/item/2009_04_23a-en
1376	2009-04-24	https://www.who.int/emergencies/disease-outbreak-news/item/2009_04_24-en
1377	2009-04-25	https://www.who.int/emergencies/disease-outbreak-news/item/2009_04_25-en
1378	2009-04-26	https://www.who.int/emergencies/disease-outbreak-news/item/2009_04_26-en
1379	2009-04-27	https://www.who.int/emergencies/disease-outbreak-news/item/2009_04_27-en
1380	2009-04-28	https://www.who.int/emergencies/disease-outbreak-news/item/2009_04_28-en
1381	2009-04-29	https://www.who.int/emergencies/disease-outbreak-news/item/2009_04_29-en
1382	2009-04-30	https://www.who.int/emergencies/disease-outbreak-news/item/2009_04_30_a-en
1383	2009-05-01	https://www.who.int/emergencies/disease-outbreak-news/item/2009_05_01a-en
1384	2009-05-02	https://www.who.int/emergencies/disease-outbreak-news/item/2009_05_02a-en
1385	2009-05-03	https://www.who.int/emergencies/disease-outbreak-news/item/2009_05_03a-en
1386	2009-05-04	https://www.who.int/emergencies/disease-outbreak-news/item/2009_05_04a-en
1387	2009-05-05	https://www.who.int/emergencies/disease-outbreak-news/item/2009_05_05a-en
1388	2009-05-06	https://www.who.int/emergencies/disease-outbreak-news/item/2009_05_06a-en
1389	2009-05-06	https://www.who.int/emergencies/disease-outbreak-news/item/2009_05_06b-en
1390	2009-05-06	https://www.who.int/emergencies/disease-outbreak-news/item/2009_05_06c-en
1391	2009-05-06	https://www.who.int/emergencies/disease-outbreak-news/item/2009_05_06d-en
1392	2009-05-07	https://www.who.int/emergencies/disease-outbreak-news/item/2009_05_07a-en
1393	2009-05-08	https://www.who.int/emergencies/disease-outbreak-news/item/2009_05_08a-en
1394	2009-05-09	https://www.who.int/emergencies/disease-outbreak-news/item/2009_05_09-en
1395	2009-05-10	https://www.who.int/emergencies/disease-outbreak-news/item/2009_05_10-en
1396	2009-05-11	https://www.who.int/emergencies/disease-outbreak-news/item/2009_05_11-en

1397	2009-05-12	https://www.who.int/emergencies/disease-outbreak-news/item/2009_05_12-en
1398	2009-05-13	https://www.who.int/emergencies/disease-outbreak-news/item/2009_05_13-en
1399	2009-05-14	https://www.who.int/emergencies/disease-outbreak-news/item/2009_05_14-en
1400	2009-05-15	https://www.who.int/emergencies/disease-outbreak-news/item/2009_05_15-en
1401	2009-05-15	https://www.who.int/emergencies/disease-outbreak-news/item/2009_05_15a-en
1402	2009-05-16	https://www.who.int/emergencies/disease-outbreak-news/item/2009_05_16-en
1403	2009-05-17	https://www.who.int/emergencies/disease-outbreak-news/item/2009_05_17-en
1404	2009-05-18	https://www.who.int/emergencies/disease-outbreak-news/item/2009_05_18-en
1405	2009-05-19	https://www.who.int/emergencies/disease-outbreak-news/item/2009_05_19-en
1406	2009-05-20	https://www.who.int/emergencies/disease-outbreak-news/item/2009_05_20-en
1407	2009-05-21	https://www.who.int/emergencies/disease-outbreak-news/item/2009_05_21-en
1408	2009-05-22	https://www.who.int/emergencies/disease-outbreak-news/item/2009_05_22a-en
1409	2009-05-23	https://www.who.int/emergencies/disease-outbreak-news/item/2009_05_23-en
1410	2009-05-25	https://www.who.int/emergencies/disease-outbreak-news/item/2009_05_25-en
1411	2009-05-26	https://www.who.int/emergencies/disease-outbreak-news/item/2009_05_26-en
1412	2009-05-27	https://www.who.int/emergencies/disease-outbreak-news/item/2009_05_27-en
1413	2009-05-27	https://www.who.int/emergencies/disease-outbreak-news/item/2009_05_27a-en
1414	2009-05-28	https://www.who.int/emergencies/disease-outbreak-news/item/2009_05_28-en
1415	2009-05-29	https://www.who.int/emergencies/disease-outbreak-news/item/2009_05_29-en
1416	2009-06-01	https://www.who.int/emergencies/disease-outbreak-news/item/2009_06_01a-en
1417	2009-06-02	https://www.who.int/emergencies/disease-outbreak-news/item/2009_06_02-en
1418	2009-06-03	https://www.who.int/emergencies/disease-outbreak-news/item/2009_06_03-en
1419	2009-06-05	https://www.who.int/emergencies/disease-outbreak-news/item/2009_06_05-en
1420	2009-06-08	https://www.who.int/emergencies/disease-outbreak-news/item/2009_06_08-en
1421	2009-06-09	https://www.who.int/emergencies/disease-outbreak-news/item/2009_06_09-en
1422	2009-06-10	https://www.who.int/emergencies/disease-outbreak-news/item/2009_06_10a-en
1423	2009-06-11	https://www.who.int/emergencies/disease-outbreak-news/item/2009_06_11-en
1424	2009-06-12	https://www.who.int/emergencies/disease-outbreak-news/item/2009_06_12-en
1425	2009-06-15	https://www.who.int/emergencies/disease-outbreak-news/item/2009_06_15-en
1426	2009-06-17	https://www.who.int/emergencies/disease-outbreak-news/item/2009_06_17-en
1427	2009-06-19	https://www.who.int/emergencies/disease-outbreak-news/item/2009_06_19-en
1428	2009-06-22	https://www.who.int/emergencies/disease-outbreak-news/item/2009_06_22-en
1429	2009-06-24	https://www.who.int/emergencies/disease-outbreak-news/item/2009_06_24-en
1430	2009-06-26	https://www.who.int/emergencies/disease-outbreak-news/item/2009_06_26-en
1431	2009-06-29	https://www.who.int/emergencies/disease-outbreak-news/item/2009_06_29-en
1432	2009-07-01	https://www.who.int/emergencies/disease-outbreak-news/item/2009_07_01-en
1433	2009-07-01	https://www.who.int/emergencies/disease-outbreak-news/item/2009_07_01a-en
1434	2009-07-03	https://www.who.int/emergencies/disease-outbreak-news/item/2009_07_03-en
1435	2009-07-06	https://www.who.int/emergencies/disease-outbreak-news/item/2009_07_06-en

1436	2009-07-17	https://www.who.int/emergencies/disease-outbreak-news/item/2009_07_17-en
1437	2009-07-27	https://www.who.int/emergencies/disease-outbreak-news/item/2009_07_27-en
1438	2009-08-04	https://www.who.int/emergencies/disease-outbreak-news/item/2009_08_04-en
1439	2009-08-11	https://www.who.int/emergencies/disease-outbreak-news/item/2009_08_11a-en
1440	2009-08-12	https://www.who.int/emergencies/disease-outbreak-news/item/2009_08_12-en
1441	2009-08-21	https://www.who.int/emergencies/disease-outbreak-news/item/2009_08_21-en
1442	2009-08-28	https://www.who.int/emergencies/disease-outbreak-news/item/2009_08_28-en
1443	2009-08-31	https://www.who.int/emergencies/disease-outbreak-news/item/2009_08_31-en
1444	2009-09-04	https://www.who.int/emergencies/disease-outbreak-news/item/2009_09_04-en
1445	2009-09-11	https://www.who.int/emergencies/disease-outbreak-news/item/2009_09_11-en
1446	2009-09-18	https://www.who.int/emergencies/disease-outbreak-news/item/2009_09_18-en
1447	2009-09-24	https://www.who.int/emergencies/disease-outbreak-news/item/2009_09_24-en
1448	2009-09-25	https://www.who.int/emergencies/disease-outbreak-news/item/2009_09_25-en
1449	2009-10-01	https://www.who.int/emergencies/disease-outbreak-news/item/2009_10_01-en
1450	2009-10-02	https://www.who.int/emergencies/disease-outbreak-news/item/2009_10_02-en
1451	2009-10-09	https://www.who.int/emergencies/disease-outbreak-news/item/2009_10_09-en
1452	2009-10-16	https://www.who.int/emergencies/disease-outbreak-news/item/2009_10_16-en
1453	2009-10-23	https://www.who.int/emergencies/disease-outbreak-news/item/2009_10_23-en
1454	2009-10-30	https://www.who.int/emergencies/disease-outbreak-news/item/2009_10_30a-en
1455	2009-11-01	https://www.who.int/emergencies/disease-outbreak-news/item/2009_11_01-en
1456	2009-11-03	https://www.who.int/emergencies/disease-outbreak-news/item/2009_11_03-en
1457	2009-11-06	https://www.who.int/emergencies/disease-outbreak-news/item/2009_11_06-en
1458	2009-11-13	https://www.who.int/emergencies/disease-outbreak-news/item/2009_11_13-en
1459	2009-11-17	https://www.who.int/emergencies/disease-outbreak-news/item/2009_11_16-en
1460	2009-11-18	https://www.who.int/emergencies/disease-outbreak-news/item/2009_11_18-en
1461	2009-11-20	https://www.who.int/emergencies/disease-outbreak-news/item/2009_11_20-en
1462	2009-11-20	https://www.who.int/emergencies/disease-outbreak-news/item/2009_11_20a-en
1463	2009-11-27	https://www.who.int/emergencies/disease-outbreak-news/item/2009_11_27-en
1464	2009-11-27	https://www.who.int/emergencies/disease-outbreak-news/item/2009_11_27a-en
1465	2009-12-01	https://www.who.int/emergencies/disease-outbreak-news/item/2009_12_01-en
1466	2009-12-04	https://www.who.int/emergencies/disease-outbreak-news/item/2009_12_04-en
1467	2009-12-11	https://www.who.int/emergencies/disease-outbreak-news/item/2009_12_11-en
1468	2009-12-11	https://www.who.int/emergencies/disease-outbreak-news/item/2009_12_11a-en
1469	2009-12-18	https://www.who.int/emergencies/disease-outbreak-news/item/2009_12_18-en
1470	2009-12-18	https://www.who.int/emergencies/disease-outbreak-news/item/2009_12_18a-en
1471	2009-12-21	https://www.who.int/emergencies/disease-outbreak-news/item/2009_12_21-en
1472	2009-12-23	https://www.who.int/emergencies/disease-outbreak-news/item/2009_12_23-en
1473	2009-12-30	https://www.who.int/emergencies/disease-outbreak-news/item/2009_12_30-en
1474	2010-01-08	https://www.who.int/emergencies/disease-outbreak-news/item/2010_01_08a-en

1475	2010-01-12	https://www.who.int/emergencies/disease-outbreak-news/item/2010_01_12-en
1476	2010-01-15	https://www.who.int/emergencies/disease-outbreak-news/item/2010_01_15-en
1477	2010-01-22	https://www.who.int/emergencies/disease-outbreak-news/item/2010_01_22-en
1478	2010-01-28	https://www.who.int/emergencies/disease-outbreak-news/item/2010_01_28-en
1479	2010-01-29	https://www.who.int/emergencies/disease-outbreak-news/item/2010_01_29-en
1480	2010-02-05	https://www.who.int/emergencies/disease-outbreak-news/item/2010_02_5-en
1481	2010-02-08	https://www.who.int/emergencies/disease-outbreak-news/item/2010_02_08-en
1482	2010-02-10	https://www.who.int/emergencies/disease-outbreak-news/item/2010_02_10-en
1483	2010-02-12	https://www.who.int/emergencies/disease-outbreak-news/item/2010_02_12a-en
1484	2010-02-17	https://www.who.int/emergencies/disease-outbreak-news/item/2010_02_17-en
1485	2010-02-19	https://www.who.int/emergencies/disease-outbreak-news/item/2010_02_19-en
1486	2010-02-24	https://www.who.int/emergencies/disease-outbreak-news/item/2010_02_24-en
1487	2010-02-26	https://www.who.int/emergencies/disease-outbreak-news/item/2010_02_26-en
1488	2010-03-04	https://www.who.int/emergencies/disease-outbreak-news/item/2010_03_04a-en
1489	2010-03-05	https://www.who.int/emergencies/disease-outbreak-news/item/2010_03_05-en
1490	2010-03-12	https://www.who.int/emergencies/disease-outbreak-news/item/2010_03_12-en
1491	2010-03-12	https://www.who.int/emergencies/disease-outbreak-news/item/_2010_03_12-en
1492	2010-03-16	https://www.who.int/emergencies/disease-outbreak-news/item/2010_03_16a-en
1493	2010-03-19	https://www.who.int/emergencies/disease-outbreak-news/item/2010_03_19-en
1494	2010-03-26	https://www.who.int/emergencies/disease-outbreak-news/item/2010_03_26-en
1495	2010-03-29	https://www.who.int/emergencies/disease-outbreak-news/item/2010_03_29a-en
1496	2010-03-30	https://www.who.int/emergencies/disease-outbreak-news/item/2010_03_30a-en
1497	2010-03-30	https://www.who.int/emergencies/disease-outbreak-news/item/_2010_03_30-en
1498	2010-04-01	https://www.who.int/emergencies/disease-outbreak-news/item/2010_04_01a-en
1499	2010-04-09	https://www.who.int/emergencies/disease-outbreak-news/item/2010_04_09-en
1500	2010-04-09	https://www.who.int/emergencies/disease-outbreak-news/item/_2010_04_09-en
1501	2010-04-16	https://www.who.int/emergencies/disease-outbreak-news/item/2010_04_16-en
1502	2010-04-21	https://www.who.int/emergencies/disease-outbreak-news/item/2010_04_21-en
1503	2010-04-23	https://www.who.int/emergencies/disease-outbreak-news/item/2010_04_23-en
1504	2010-04-23	https://www.who.int/emergencies/disease-outbreak-news/item/2010_04_23a-en
1505	2010-04-29	https://www.who.int/emergencies/disease-outbreak-news/item/2010_04_29-en
1506	2010-04-30	https://www.who.int/emergencies/disease-outbreak-news/item/2010_04_30a-en
1507	2010-05-04	https://www.who.int/emergencies/disease-outbreak-news/item/2010_05_04a-en
1508	2010-05-06	https://www.who.int/emergencies/disease-outbreak-news/item/2010_05_06-en
1509	2010-05-07	https://www.who.int/emergencies/disease-outbreak-news/item/2010_05_07-en
1510	2010-05-12	https://www.who.int/emergencies/disease-outbreak-news/item/2010_05_12-en
1511	2010-05-14	https://www.who.int/emergencies/disease-outbreak-news/item/2010_05_14-en
1512	2010-05-21	https://www.who.int/emergencies/disease-outbreak-news/item/2010_05_21-en
1513	2010-05-27	https://www.who.int/emergencies/disease-outbreak-news/item/2010_05_27-en

1514	2010-05-28	https://www.who.int/emergencies/disease-outbreak-news/item/2010_05_28-en
1515	2010-06-04	https://www.who.int/emergencies/disease-outbreak-news/item/2010_06_04-en
1516	2010-06-08	https://www.who.int/emergencies/disease-outbreak-news/item/2010_06_08-en
1517	2010-06-11	https://www.who.int/emergencies/disease-outbreak-news/item/2010_06_11-en
1518	2010-06-18	https://www.who.int/emergencies/disease-outbreak-news/item/2010_06_18-en
1519	2010-06-25	https://www.who.int/emergencies/disease-outbreak-news/item/2010_06_25-en
1520	2010-06-29	https://www.who.int/emergencies/disease-outbreak-news/item/2010_06_29-en
1521	2010-07-02	https://www.who.int/emergencies/disease-outbreak-news/item/2010_07_02a-en
1522	2010-07-05	https://www.who.int/emergencies/disease-outbreak-news/item/2010_07_05-en
1523	2010-07-07	https://www.who.int/emergencies/disease-outbreak-news/item/2010_07_07-en
1524	2010-07-09	https://www.who.int/emergencies/disease-outbreak-news/item/2010_07_09-en
1525	2010-07-16	https://www.who.int/emergencies/disease-outbreak-news/item/2010_07_16-en
1526	2010-07-19	https://www.who.int/emergencies/disease-outbreak-news/item/2010_07_19a-en
1527	2010-07-22	https://www.who.int/emergencies/disease-outbreak-news/item/2010_07_22-en
1528	2010-07-23	https://www.who.int/emergencies/disease-outbreak-news/item/2010_07_23-en
1529	2010-07-23	https://www.who.int/emergencies/disease-outbreak-news/item/2010_07_23a-en
1530	2010-07-29	https://www.who.int/emergencies/disease-outbreak-news/item/2010_07_29-en
1531	2010-07-30	https://www.who.int/emergencies/disease-outbreak-news/item/2010_07_30-en
1532	2010-08-03	https://www.who.int/emergencies/disease-outbreak-news/item/2010_08_03-en
1533	2010-08-06	https://www.who.int/emergencies/disease-outbreak-news/item/2010_08_06-en
1534	2010-08-10	https://www.who.int/emergencies/disease-outbreak-news/item/2010_08_10-en
1535	2010-08-12	https://www.who.int/emergencies/disease-outbreak-news/item/2010_08_12-en
1536	2010-08-13	https://www.who.int/emergencies/disease-outbreak-news/item/2010_08_13-en
1537	2010-08-20	https://www.who.int/emergencies/disease-outbreak-news/item/2010_08_20-en
1538	2010-08-27	https://www.who.int/emergencies/disease-outbreak-news/item/2010_08_27-en
1539	2010-08-31	https://www.who.int/emergencies/disease-outbreak-news/item/2010_08_31-en
1540	2010-09-08	https://www.who.int/emergencies/disease-outbreak-news/item/2010_09_08-en
1541	2010-09-10	https://www.who.int/emergencies/disease-outbreak-news/item/2010_09_10-en
1542	2010-10-05	https://www.who.int/emergencies/disease-outbreak-news/item/2010_10_05-en
1543	2010-10-08	https://www.who.int/emergencies/disease-outbreak-news/item/2010_10_08-en
1544	2010-10-18	https://www.who.int/emergencies/disease-outbreak-news/item/2010_10_18-en
1545	2010-10-25	https://www.who.int/emergencies/disease-outbreak-news/item/2010_10_25-en
1546	2010-10-25	https://www.who.int/emergencies/disease-outbreak-news/item/2010_10_25a-en
1547	2010-10-26	https://www.who.int/emergencies/disease-outbreak-news/item/2010_10_26-en
1548	2010-10-28	https://www.who.int/emergencies/disease-outbreak-news/item/2010_10_28-en
1549	2010-11-04	https://www.who.int/emergencies/disease-outbreak-news/item/2010_11_04a-en
1550	2010-11-09	https://www.who.int/emergencies/disease-outbreak-news/item/2010_11_09-en
1551	2010-11-13	https://www.who.int/emergencies/disease-outbreak-news/item/2010_11_13-en
1552	2010-11-17	https://www.who.int/emergencies/disease-outbreak-news/item/2010_11_17-en

1553	2010-11-19	https://www.who.int/emergencies/disease-outbreak-news/item/2010_11_19-en
1554	2010-11-24	https://www.who.int/emergencies/disease-outbreak-news/item/2010_11_24-en
1555	2010-12-08	https://www.who.int/emergencies/disease-outbreak-news/item/2010_12_08-en
1556	2010-12-09	https://www.who.int/emergencies/disease-outbreak-news/item/2010_12_09-en
1557	2010-12-29	https://www.who.int/emergencies/disease-outbreak-news/item/2010_12_29-en
1558	2011-01-05	https://www.who.int/emergencies/disease-outbreak-news/item/2011_01_05-en
1559	2011-01-13	https://www.who.int/emergencies/disease-outbreak-news/item/2011_01_12-en
1560	2011-01-19	https://www.who.int/emergencies/disease-outbreak-news/item/2011_01_19-en
1561	2011-01-20	https://www.who.int/emergencies/disease-outbreak-news/item/2011_01_20-en
1562	2011-01-25	https://www.who.int/emergencies/disease-outbreak-news/item/2011_01_25-en
1563	2011-02-01	https://www.who.int/emergencies/disease-outbreak-news/item/2011_02_01-en
1564	2011-02-02	https://www.who.int/emergencies/disease-outbreak-news/item/2011_02_02-en
1565	2011-02-09	https://www.who.int/emergencies/disease-outbreak-news/item/2011_02_09-en
1566	2011-02-25	https://www.who.int/emergencies/disease-outbreak-news/item/2011_02_25-en
1567	2011-02-28	https://www.who.int/emergencies/disease-outbreak-news/item/2011_02_28-en
1568	2011-03-02	https://www.who.int/emergencies/disease-outbreak-news/item/2011_03_02-en
1569	2011-03-07	https://www.who.int/emergencies/disease-outbreak-news/item/2011_03_07-en
1570	2011-03-08	https://www.who.int/emergencies/disease-outbreak-news/item/2011_03_08-en
1571	2011-03-10	https://www.who.int/emergencies/disease-outbreak-news/item/2011_03_10-en
1572	2011-03-11	https://www.who.int/emergencies/disease-outbreak-news/item/2011_03_11-en
1573	2011-03-14	https://www.who.int/emergencies/disease-outbreak-news/item/2011_03_14-en
1574	2011-03-15	https://www.who.int/emergencies/disease-outbreak-news/item/2011_03_15-en
1575	2011-03-16	https://www.who.int/emergencies/disease-outbreak-news/item/2011_03_16-en
1576	2011-03-25	https://www.who.int/emergencies/disease-outbreak-news/item/2011_03_25-en
1577	2011-03-29	https://www.who.int/emergencies/disease-outbreak-news/item/2011_03_29-en
1578	2011-04-01	https://www.who.int/emergencies/disease-outbreak-news/item/2011_04_01-en
1579	2011-04-06	https://www.who.int/emergencies/disease-outbreak-news/item/2011_04_06-en
1580	2011-04-11	https://www.who.int/emergencies/disease-outbreak-news/item/2011_04_11a-en
1581	2011-04-11	https://www.who.int/emergencies/disease-outbreak-news/item/2011_04_11b-en
1582	2011-04-21	https://www.who.int/emergencies/disease-outbreak-news/item/2011_04_21c-en
1583	2011-04-21	https://www.who.int/emergencies/disease-outbreak-news/item/2011_04_21d-en
1584	2011-04-21	https://www.who.int/emergencies/disease-outbreak-news/item/2011_04_21a-en
1585	2011-05-13	https://www.who.int/emergencies/disease-outbreak-news/item/2011_05_13-en
1586	2011-05-18	https://www.who.int/emergencies/disease-outbreak-news/item/2011_05_18-en
1587	2011-05-27	https://www.who.int/emergencies/disease-outbreak-news/item/2011_05_27-en
1588	2011-06-01	https://www.who.int/emergencies/disease-outbreak-news/item/2011_06_01-en
1589	2011-06-02	https://www.who.int/emergencies/disease-outbreak-news/item/2011_06_02-en
1590	2011-06-03	https://www.who.int/emergencies/disease-outbreak-news/item/2011_06_03-en
1591	2011-06-10	https://www.who.int/emergencies/disease-outbreak-news/item/2011_06_10a-en

1592	2011-06-16	https://www.who.int/emergencies/disease-outbreak-news/item/2011_06_16-en
1593	2011-06-22	https://www.who.int/emergencies/disease-outbreak-news/item/2011_06_22-en
1594	2011-07-07	https://www.who.int/emergencies/disease-outbreak-news/item/2011_07_07-en
1595	2011-07-22	https://www.who.int/emergencies/disease-outbreak-news/item/2011_07_22-en
1596	2011-08-02	https://www.who.int/emergencies/disease-outbreak-news/item/2011_08_02-en
1597	2011-08-09	https://www.who.int/emergencies/disease-outbreak-news/item/2011_08_09-en
1598	2011-08-15	https://www.who.int/emergencies/disease-outbreak-news/item/2011_08_15-en
1599	2011-08-16	https://www.who.int/emergencies/disease-outbreak-news/item/2011_08_16-en
1600	2011-08-19	https://www.who.int/emergencies/disease-outbreak-news/item/2011_08_19-en
1601	2011-09-01	https://www.who.int/emergencies/disease-outbreak-news/item/2011_09_01-en
1602	2011-09-20	https://www.who.int/emergencies/disease-outbreak-news/item/2011_09_20-en
1603	2011-10-07	https://www.who.int/emergencies/disease-outbreak-news/item/2011_10_07-en
1604	2011-10-10	https://www.who.int/emergencies/disease-outbreak-news/item/2011_10_10-en
1605	2011-10-26	https://www.who.int/emergencies/disease-outbreak-news/item/2011_10_26-en
1606	2011-11-02	https://www.who.int/emergencies/disease-outbreak-news/item/2011_11_02-en
1607	2011-11-11	https://www.who.int/emergencies/disease-outbreak-news/item/2011_11_11-en
1608	2011-11-15	https://www.who.int/emergencies/disease-outbreak-news/item/2011_11_15-en
1609	2011-11-24	https://www.who.int/emergencies/disease-outbreak-news/item/2011_11_24-en
1610	2011-11-29	https://www.who.int/emergencies/disease-outbreak-news/item/2011_11_29-en
1611	2011-12-01	https://www.who.int/emergencies/disease-outbreak-news/item/2011_12_01-en
1612	2011-12-15	https://www.who.int/emergencies/disease-outbreak-news/item/2011_12_15-en
1613	2011-12-21	https://www.who.int/emergencies/disease-outbreak-news/item/2011_12_21-en
1614	2012-01-05	https://www.who.int/emergencies/disease-outbreak-news/item/2012_01_05-en
1615	2012-01-05	https://www.who.int/emergencies/disease-outbreak-news/item/2012_01_05b-en
1616	2012-01-11	https://www.who.int/emergencies/disease-outbreak-news/item/2012_01_11-en
1617	2012-01-16	https://www.who.int/emergencies/disease-outbreak-news/item/2012_01_16-en
1618	2012-01-17	https://www.who.int/emergencies/disease-outbreak-news/item/2012_01_17-en
1619	2012-01-18	https://www.who.int/emergencies/disease-outbreak-news/item/2012_01_18-en
1620	2012-01-19	https://www.who.int/emergencies/disease-outbreak-news/item/2012_01_19-en
1621	2012-01-19	https://www.who.int/emergencies/disease-outbreak-news/item/2012_01_19b-en
1622	2012-01-20	https://www.who.int/emergencies/disease-outbreak-news/item/2012_01_20-en
1623	2012-01-24	https://www.who.int/emergencies/disease-outbreak-news/item/2012_01_24-en
1624	2012-02-03	https://www.who.int/emergencies/disease-outbreak-news/item/2012_02_03-en
1625	2012-02-03	https://www.who.int/emergencies/disease-outbreak-news/item/2012_02_03b-en
1626	2012-02-08	https://www.who.int/emergencies/disease-outbreak-news/item/2012_02_08-en
1627	2012-02-21	https://www.who.int/emergencies/disease-outbreak-news/item/2012_02_21-en
1628	2012-02-22	https://www.who.int/emergencies/disease-outbreak-news/item/2012_02_22-en
1629	2012-02-24	https://www.who.int/emergencies/disease-outbreak-news/item/2012_02_24-en
1630	2012-02-28	https://www.who.int/emergencies/disease-outbreak-news/item/2012_02_28-en

1631	2012-03-01	https://www.who.int/emergencies/disease-outbreak-news/item/2012_03_01-en
1632	2012-03-02	https://www.who.int/emergencies/disease-outbreak-news/item/2012_03_02-en
1633	2012-03-05	https://www.who.int/emergencies/disease-outbreak-news/item/2012_03_05-en
1634	2012-03-07	https://www.who.int/emergencies/disease-outbreak-news/item/2012_03_07-en
1635	2012-03-12	https://www.who.int/emergencies/disease-outbreak-news/item/2012_03_12b-en
1636	2012-03-19	https://www.who.int/emergencies/disease-outbreak-news/item/2012_03_19-en
1637	2012-03-23	https://www.who.int/emergencies/disease-outbreak-news/item/2012_03_23-en
1638	2012-03-26	https://www.who.int/emergencies/disease-outbreak-news/item/2012_03_26-en
1639	2012-04-02	https://www.who.int/emergencies/disease-outbreak-news/item/2012_04_02-en
1640	2012-04-04	https://www.who.int/emergencies/disease-outbreak-news/item/2012_04_04-en
1641	2012-04-05	https://www.who.int/emergencies/disease-outbreak-news/item/2012_04_05-en
1642	2012-04-12	https://www.who.int/emergencies/disease-outbreak-news/item/2012_04_12-en
1643	2012-05-02	https://www.who.int/emergencies/disease-outbreak-news/item/2012_05_02-en
1644	2012-05-24	https://www.who.int/emergencies/disease-outbreak-news/item/2012_05_24-en
1645	2012-05-29	https://www.who.int/emergencies/disease-outbreak-news/item/2012_05_29-en
1646	2012-06-05	https://www.who.int/emergencies/disease-outbreak-news/item/2012_06_05-en
1647	2012-06-07	https://www.who.int/emergencies/disease-outbreak-news/item/2012_06_07-en
1648	2012-07-04	https://www.who.int/emergencies/disease-outbreak-news/item/2012_07_04-en
1649	2012-07-06	https://www.who.int/emergencies/disease-outbreak-news/item/2012_07_06-en
1650	2012-07-06	https://www.who.int/emergencies/disease-outbreak-news/item/2012_07_06a-en
1651	2012-07-09	https://www.who.int/emergencies/disease-outbreak-news/item/2012_07_09-en
1652	2012-07-13	https://www.who.int/emergencies/disease-outbreak-news/item/2012_07_13-en
1653	2012-07-23	https://www.who.int/emergencies/disease-outbreak-news/item/2012_07_23-en
1654	2012-07-29	https://www.who.int/emergencies/disease-outbreak-news/item/2012_07_29-en
1655	2012-08-03	https://www.who.int/emergencies/disease-outbreak-news/item/2012_08_03-en
1656	2012-08-10	https://www.who.int/emergencies/disease-outbreak-news/item/2012_08_10b-en
1657	2012-08-14	https://www.who.int/emergencies/disease-outbreak-news/item/2012_08_14-en
1658	2012-08-17	https://www.who.int/emergencies/disease-outbreak-news/item/2012_08_17-en
1659	2012-08-17	https://www.who.int/emergencies/disease-outbreak-news/item/2012_08_18-en
1660	2012-08-21	https://www.who.int/emergencies/disease-outbreak-news/item/2012_08_21-en
1661	2012-08-22	https://www.who.int/emergencies/disease-outbreak-news/item/2012_08_22-en
1662	2012-08-30	https://www.who.int/emergencies/disease-outbreak-news/item/2012_08_30-en
1663	2012-08-30	https://www.who.int/emergencies/disease-outbreak-news/item/2012_08_30a-en
1664	2012-09-03	https://www.who.int/emergencies/disease-outbreak-news/item/2012_09_03-en
1665	2012-09-04	https://www.who.int/emergencies/disease-outbreak-news/item/2012_09_04-en
1666	2012-09-05	https://www.who.int/emergencies/disease-outbreak-news/item/2012_09_05-en
1667	2012-09-08	https://www.who.int/emergencies/disease-outbreak-news/item/2012_09_08-en
1668	2012-09-12	https://www.who.int/emergencies/disease-outbreak-news/item/2012_09_12-en
1669	2012-09-14	https://www.who.int/emergencies/disease-outbreak-news/item/2012_09_14-en

1670	2012-09-18	https://www.who.int/emergencies/disease-outbreak-news/item/2012_09_18b-en
1671	2012-09-23	https://www.who.int/emergencies/disease-outbreak-news/item/2012_09_23-en
1672	2012-09-25	https://www.who.int/emergencies/disease-outbreak-news/item/2012_09_25-en
1673	2012-09-27	https://www.who.int/emergencies/disease-outbreak-news/item/2012_09_27-en
1674	2012-09-28	https://www.who.int/emergencies/disease-outbreak-news/item/2012_09_28-en
1675	2012-09-29	https://www.who.int/emergencies/disease-outbreak-news/item/2012_09_29-en
1676	2012-10-04	https://www.who.int/emergencies/disease-outbreak-news/item/2012_10_04-en
1677	2012-10-08	https://www.who.int/emergencies/disease-outbreak-news/item/2012_10_08a-en
1678	2012-10-10	https://www.who.int/emergencies/disease-outbreak-news/item/2012_10_10-en
1679	2012-10-17	https://www.who.int/emergencies/disease-outbreak-news/item/2012_10_17-en
1680	2012-10-21	https://www.who.int/emergencies/disease-outbreak-news/item/2012_10_21-en
1681	2012-10-22	https://www.who.int/emergencies/disease-outbreak-news/item/2012_10_22-en
1682	2012-10-26	https://www.who.int/emergencies/disease-outbreak-news/item/2012_10_26-en
1683	2012-10-31	https://www.who.int/emergencies/disease-outbreak-news/item/2012_10_31-en
1684	2012-11-01	https://www.who.int/emergencies/disease-outbreak-news/item/2012_11_01-en
1685	2012-11-13	https://www.who.int/emergencies/disease-outbreak-news/item/2012_11_13-en
1686	2012-11-17	https://www.who.int/emergencies/disease-outbreak-news/item/2012_11_17-en
1687	2012-11-22	https://www.who.int/emergencies/disease-outbreak-news/item/2012_11_22-en
1688	2012-11-23	https://www.who.int/emergencies/disease-outbreak-news/item/2012_11_23_ebola-en
1689	2012-11-23	https://www.who.int/emergencies/disease-outbreak-news/item/2012_11_23_update-en
1690	2012-11-30	https://www.who.int/emergencies/disease-outbreak-news/item/2012_11_30_ebola-en
1691	2012-12-06	https://www.who.int/emergencies/disease-outbreak-news/item/2012_12_06-en
1692	2012-12-12	https://www.who.int/emergencies/disease-outbreak-news/item/2012_12_12-en
1693	2013-02-01	https://www.who.int/emergencies/disease-outbreak-news/item/2013_02_01-en
1694	2013-02-11	https://www.who.int/emergencies/disease-outbreak-news/item/2013_02_11b-en
1695	2013-02-12	https://www.who.int/emergencies/disease-outbreak-news/item/2013_02_12-en
1696	2013-02-13	https://www.who.int/emergencies/disease-outbreak-news/item/2013_02_13-en
1697	2013-02-14	https://www.who.int/emergencies/disease-outbreak-news/item/2013_02_14-en
1698	2013-02-16	https://www.who.int/emergencies/disease-outbreak-news/item/2013_02_16-en
1699	2013-02-21	https://www.who.int/emergencies/disease-outbreak-news/item/2013_02_21-en
1700	2013-03-06	https://www.who.int/emergencies/disease-outbreak-news/item/2013_03_06-en
1701	2013-03-12	https://www.who.int/emergencies/disease-outbreak-news/item/2013_03_12-en
1702	2013-03-23	https://www.who.int/emergencies/disease-outbreak-news/item/2013_03_23-en
1703	2013-03-26	https://www.who.int/emergencies/disease-outbreak-news/item/2013_03_26-en
1704	2013-04-01	https://www.who.int/emergencies/disease-outbreak-news/item/2013_04_01-en
1705	2013-04-03	https://www.who.int/emergencies/disease-outbreak-news/item/2013_04_03-en
1706	2013-04-04	https://www.who.int/emergencies/disease-outbreak-news/item/2013_04_04-en
1707	2013-04-05	https://www.who.int/emergencies/disease-outbreak-news/item/2013_04_05-en
1708	2013-04-06	https://www.who.int/emergencies/disease-outbreak-news/item/2013_04_06-en

1709	2013-04-07	https://www.who.int/emergencies/disease-outbreak-news/item/2013_04_07-en
1710	2013-04-09	https://www.who.int/emergencies/disease-outbreak-news/item/2013_04_09-en
1711	2013-04-10	https://www.who.int/emergencies/disease-outbreak-news/item/2013_04_10-en
1712	2013-04-11	https://www.who.int/emergencies/disease-outbreak-news/item/2013_04_11-en
1713	2013-04-12	https://www.who.int/emergencies/disease-outbreak-news/item/2013_04_12-en
1714	2013-04-13	https://www.who.int/emergencies/disease-outbreak-news/item/2013_04_13-en
1715	2013-04-14	https://www.who.int/emergencies/disease-outbreak-news/item/2013_04_14-en
1716	2013-04-15	https://www.who.int/emergencies/disease-outbreak-news/item/2013_04_15-en
1717	2013-04-16	https://www.who.int/emergencies/disease-outbreak-news/item/2013_04_16-en
1718	2013-04-17	https://www.who.int/emergencies/disease-outbreak-news/item/2013_04_17-en
1719	2013-04-18	https://www.who.int/emergencies/disease-outbreak-news/item/2013_04_18-en
1720	2013-04-19	https://www.who.int/emergencies/disease-outbreak-news/item/2013_04_19-en
1721	2013-04-20	https://www.who.int/emergencies/disease-outbreak-news/item/2013_04_20-en
1722	2013-04-21	https://www.who.int/emergencies/disease-outbreak-news/item/2013_04_21-en
1723	2013-04-22	https://www.who.int/emergencies/disease-outbreak-news/item/2013_04_22-en
1724	2013-04-23	https://www.who.int/emergencies/disease-outbreak-news/item/2013_04_23-en
1725	2013-04-25	https://www.who.int/emergencies/disease-outbreak-news/item/2013_04_25-en
1726	2013-04-29	https://www.who.int/emergencies/disease-outbreak-news/item/2013_04_29-en
1727	2013-05-02	https://www.who.int/emergencies/disease-outbreak-news/item/2013_05_02_ncov-en
1728	2013-05-03	https://www.who.int/emergencies/disease-outbreak-news/item/2013_05_03_ncov-en
1729	2013-05-06	https://www.who.int/emergencies/disease-outbreak-news/item/2013_05_06_ncov-en
1730	2013-05-07	https://www.who.int/emergencies/disease-outbreak-news/item/2013_05_07-en
1731	2013-05-08	https://www.who.int/emergencies/disease-outbreak-news/item/2013_05_08_ncov-en
1732	2013-05-09	https://www.who.int/emergencies/disease-outbreak-news/item/2013_05_09_ncov-en
1733	2013-05-11	https://www.who.int/emergencies/disease-outbreak-news/item/2013_05_11-en
1734	2013-05-12	https://www.who.int/emergencies/disease-outbreak-news/item/2013_05_12-en
1735	2013-05-14	https://www.who.int/emergencies/disease-outbreak-news/item/2013_05_14_ncov-en
1736	2013-05-15	https://www.who.int/emergencies/disease-outbreak-news/item/2013_05_15_ncov-en
1737	2013-05-17	https://www.who.int/emergencies/disease-outbreak-news/item/2013_05_17-en
1738	2013-05-18	https://www.who.int/emergencies/disease-outbreak-news/item/2013_05_18_ncov-en
1739	2013-05-22	https://www.who.int/emergencies/disease-outbreak-news/item/2013_05_22_ncov-en
1740	2013-05-23	https://www.who.int/emergencies/disease-outbreak-news/item/2013_05_23_ncov-en
1741	2013-05-29	https://www.who.int/emergencies/disease-outbreak-news/item/2013_05_29_ncov-en
1742	2013-05-31	https://www.who.int/emergencies/disease-outbreak-news/item/2013_05_31_ncov-en
1743	2013-06-01	https://www.who.int/emergencies/disease-outbreak-news/item/2013_06_01_ncov-en
1744	2013-06-02	https://www.who.int/emergencies/disease-outbreak-news/item/2013_06_02_ncov-en
1745	2013-06-03	https://www.who.int/emergencies/disease-outbreak-news/item/2013_06_03-en
1746	2013-06-05	https://www.who.int/emergencies/disease-outbreak-news/item/2013_06_05-en
1747	2013-06-06	https://www.who.int/emergencies/disease-outbreak-news/item/2013_06_06_menin-en

1748	2013-06-07	https://www.who.int/emergencies/disease-outbreak-news/item/2013_06_07-en
1749	2013-06-14	https://www.who.int/emergencies/disease-outbreak-news/item/2013_06_14-en
1750	2013-06-14	https://www.who.int/emergencies/disease-outbreak-news/item/2013_06_14_yellowfever-en
1751	2013-06-15	https://www.who.int/emergencies/disease-outbreak-news/item/2013_06_15-en
1752	2013-06-17	https://www.who.int/emergencies/disease-outbreak-news/item/2013_06_17-en
1753	2013-06-22	https://www.who.int/emergencies/disease-outbreak-news/item/2013_06_22-en
1754	2013-06-23	https://www.who.int/emergencies/disease-outbreak-news/item/2013_06_23-en
1755	2013-06-26	https://www.who.int/emergencies/disease-outbreak-news/item/2013_06_26-en
1756	2013-07-04	https://www.who.int/emergencies/disease-outbreak-news/item/2013_07_04-en
1757	2013-07-05	https://www.who.int/emergencies/disease-outbreak-news/item/2013_07_05-en
1758	2013-07-07	https://www.who.int/emergencies/disease-outbreak-news/item/2013_07_07-en
1759	2013-07-11	https://www.who.int/emergencies/disease-outbreak-news/item/2013_07_11-en
1760	2013-07-13	https://www.who.int/emergencies/disease-outbreak-news/item/2013_07_13-en
1761	2013-07-15	https://www.who.int/emergencies/disease-outbreak-news/item/2013_07_15-en
1762	2013-07-18	https://www.who.int/emergencies/disease-outbreak-news/item/2013_07_18-en
1763	2013-07-19	https://www.who.int/emergencies/disease-outbreak-news/item/2013_07_19-en
1764	2013-07-20	https://www.who.int/emergencies/disease-outbreak-news/item/2013_07_20-en
1765	2013-07-21	https://www.who.int/emergencies/disease-outbreak-news/item/2013_07_21-en
1766	2013-07-29	https://www.who.int/emergencies/disease-outbreak-news/item/2013_07_29-en
1767	2013-08-01	https://www.who.int/emergencies/disease-outbreak-news/item/2013_08_01-en
1768	2013-08-11	https://www.who.int/emergencies/disease-outbreak-news/item/2013_08_11-en
1769	2013-08-15	https://www.who.int/emergencies/disease-outbreak-news/item/2013_08_15-en
1770	2013-08-28	https://www.who.int/emergencies/disease-outbreak-news/item/2013_08_28-en
1771	2013-08-29	https://www.who.int/emergencies/disease-outbreak-news/item/2013_08_29-en
1772	2013-08-30	https://www.who.int/emergencies/disease-outbreak-news/item/2013_08_30-en
1773	2013-09-06	https://www.who.int/emergencies/disease-outbreak-news/item/2013_09_06-en
1774	2013-09-07	https://www.who.int/emergencies/disease-outbreak-news/item/2013_09_07-en
1775	2013-09-19	https://www.who.int/emergencies/disease-outbreak-news/item/2013_09_19-en
1776	2013-09-20	https://www.who.int/emergencies/disease-outbreak-news/item/2013_09_20-en
1777	2013-09-20	https://www.who.int/emergencies/disease-outbreak-news/item/2013_09_20_polio-en
1778	2013-10-01	https://www.who.int/emergencies/disease-outbreak-news/item/2013_10_01-en
1779	2013-10-04	https://www.who.int/emergencies/disease-outbreak-news/item/2013_10_04-en
1780	2013-10-08	https://www.who.int/emergencies/disease-outbreak-news/item/2013_10_08-en
1781	2013-10-14	https://www.who.int/emergencies/disease-outbreak-news/item/2013_10_14-en
1782	2013-10-16	https://www.who.int/emergencies/disease-outbreak-news/item/2013_10_16-en
1783	2013-10-18	https://www.who.int/emergencies/disease-outbreak-news/item/2013_10_18-en
1784	2013-10-19	https://www.who.int/emergencies/disease-outbreak-news/item/2013_10_19_cholera-en
1785	2013-10-19	https://www.who.int/emergencies/disease-outbreak-news/item/2013_10_19_polio-en
1786	2013-10-24	https://www.who.int/emergencies/disease-outbreak-news/item/2013_10_24a-en

1787	2013-10-28	https://www.who.int/emergencies/disease-outbreak-news/item/2013_10_28-en
1788	2013-10-29	https://www.who.int/emergencies/disease-outbreak-news/item/2013_10_29a-en
1789	2013-10-31	https://www.who.int/emergencies/disease-outbreak-news/item/2013_10_31-en
1790	2013-11-04	https://www.who.int/emergencies/disease-outbreak-news/item/2013_11_04-en
1791	2013-11-06	https://www.who.int/emergencies/disease-outbreak-news/item/2013_11_06-en
1792	2013-11-07	https://www.who.int/emergencies/disease-outbreak-news/item/2013_11_07-en
1793	2013-11-10	https://www.who.int/emergencies/disease-outbreak-news/item/2013_11_10-en
1794	2013-11-11	https://www.who.int/emergencies/disease-outbreak-news/item/2013_11_11polio-en
1795	2013-11-11	https://www.who.int/emergencies/disease-outbreak-news/item/2013_11_11_coronavirus-en
1796	2013-11-13	https://www.who.int/emergencies/disease-outbreak-news/item/2013_11_13-en
1797	2013-11-15	https://www.who.int/emergencies/disease-outbreak-news/item/2013_11_15-en
1798	2013-11-18	https://www.who.int/emergencies/disease-outbreak-news/item/2013_11_18-en
1799	2013-11-21	https://www.who.int/emergencies/disease-outbreak-news/item/2013_11_21-en
1800	2013-11-25	https://www.who.int/emergencies/disease-outbreak-news/item/2013_11_25-en
1801	2013-11-26	https://www.who.int/emergencies/disease-outbreak-news/item/2013_11_26polio-en
1802	2013-11-29	https://www.who.int/emergencies/disease-outbreak-news/item/2013_11_29-en
1803	2013-12-02	https://www.who.int/emergencies/disease-outbreak-news/item/2013_12_02-en
1804	2013-12-03	https://www.who.int/emergencies/disease-outbreak-news/item/2012_12_03-en
1805	2013-12-10	https://www.who.int/emergencies/disease-outbreak-news/item/2013_12_10a-en
1806	2013-12-17	https://www.who.int/emergencies/disease-outbreak-news/item/2013_12_17-en
1807	2013-12-17	https://www.who.int/emergencies/disease-outbreak-news/item/2013_12_17influenza-en
1808	2013-12-22	https://www.who.int/emergencies/disease-outbreak-news/item/2013_12_22-en
1809	2013-12-27	https://www.who.int/emergencies/disease-outbreak-news/item/2013_12_27-en
1810	2013-12-31	https://www.who.int/emergencies/disease-outbreak-news/item/2013_12_31-en
1811	2014-01-03	https://www.who.int/emergencies/disease-outbreak-news/item/2014_01_03-en
1812	2014-01-06	https://www.who.int/emergencies/disease-outbreak-news/item/2014_01_06-en
1813	2014-01-07	https://www.who.int/emergencies/disease-outbreak-news/item/2014_01_07-en
1814	2014-01-09	https://www.who.int/emergencies/disease-outbreak-news/item/2014_01_09_h5n1-en
1815	2014-01-09	https://www.who.int/emergencies/disease-outbreak-news/item/2014_01_09_h7n9-en
1816	2014-01-10	https://www.who.int/emergencies/disease-outbreak-news/item/2014_01_10_h7n9-en
1817	2014-01-13	https://www.who.int/emergencies/disease-outbreak-news/item/2014_01_13-en
1818	2014-01-14	https://www.who.int/emergencies/disease-outbreak-news/item/2014_01_14-en
1819	2014-01-15	https://www.who.int/emergencies/disease-outbreak-news/item/2014_01_15-en
1820	2014-01-16	https://www.who.int/emergencies/disease-outbreak-news/item/2014_01_16-en
1821	2014-01-17	https://www.who.int/emergencies/disease-outbreak-news/item/2014_01_17-en
1822	2014-01-20	https://www.who.int/emergencies/disease-outbreak-news/item/2014_01_20bis-en
1823	2014-01-22	https://www.who.int/emergencies/disease-outbreak-news/item/2014_01_22-en
1824	2014-01-23	https://www.who.int/emergencies/disease-outbreak-news/item/2014_01_23-en
1825	2014-01-25	https://www.who.int/emergencies/disease-outbreak-news/item/2014_01_25-en

1826	2014-01-27	https://www.who.int/emergencies/disease-outbreak-news/item/2014_01_27mers-en
1827	2014-01-29	https://www.who.int/emergencies/disease-outbreak-news/item/2014_01_29-en
1828	2014-01-30	https://www.who.int/emergencies/disease-outbreak-news/item/2014_01_30-en
1829	2014-01-31	https://www.who.int/emergencies/disease-outbreak-news/item/2014_01_31-en
1830	2014-02-01	https://www.who.int/emergencies/disease-outbreak-news/item/2014_02_01-en
1831	2014-02-03	https://www.who.int/emergencies/disease-outbreak-news/item/2014_02_03-en
1832	2014-02-04	https://www.who.int/emergencies/disease-outbreak-news/item/2014_02_04mers-en
1833	2014-02-05	https://www.who.int/emergencies/disease-outbreak-news/item/2014_02_05bis-en
1834	2014-02-07	https://www.who.int/emergencies/disease-outbreak-news/item/2014_02_07mers-en
1835	2014-02-10	https://www.who.int/emergencies/disease-outbreak-news/item/2014_02_10-en
1836	2014-02-11	https://www.who.int/emergencies/disease-outbreak-news/item/2014_02_11-en
1837	2014-02-14	https://www.who.int/emergencies/disease-outbreak-news/item/2014_02_14-en
1838	2014-02-17	https://www.who.int/emergencies/disease-outbreak-news/item/2014_02_17-en
1839	2014-02-17	https://www.who.int/emergencies/disease-outbreak-news/item/2014_02_17_ah7n9-en
1840	2014-02-18	https://www.who.int/emergencies/disease-outbreak-news/item/2014_02_18-en
1841	2014-02-24	https://www.who.int/emergencies/disease-outbreak-news/item/2014_02_24-en
1842	2014-02-27	https://www.who.int/emergencies/disease-outbreak-news/item/2014_02_27b-en
1843	2014-02-28	https://www.who.int/emergencies/disease-outbreak-news/item/2014_02_28-en
1844	2014-03-03	https://www.who.int/emergencies/disease-outbreak-news/item/2014_03_03-en
1845	2014-03-05	https://www.who.int/emergencies/disease-outbreak-news/item/2014_03_05-en
1846	2014-03-07	https://www.who.int/emergencies/disease-outbreak-news/item/2014_03_07-en
1847	2014-03-10	https://www.who.int/emergencies/disease-outbreak-news/item/2014_03_10-en
1848	2014-03-11	https://www.who.int/emergencies/disease-outbreak-news/item/2014_03_11b-en
1849	2014-03-11	https://www.who.int/emergencies/disease-outbreak-news/item/2014_03_11c-en
1850	2014-03-12	https://www.who.int/emergencies/disease-outbreak-news/item/2014_03_12-en
1851	2014-03-17	https://www.who.int/emergencies/disease-outbreak-news/item/2014_03_17_h7n9-en
1852	2014-03-17	https://www.who.int/emergencies/disease-outbreak-news/item/2014_03_17_polio-en
1853	2014-03-18	https://www.who.int/emergencies/disease-outbreak-news/item/2014_03_18-en
1854	2014-03-20	https://www.who.int/emergencies/disease-outbreak-news/item/2014_03_20_h7n9bis-en
1855	2014-03-20	https://www.who.int/emergencies/disease-outbreak-news/item/2014_03_20_mers-en
1856	2014-03-21	https://www.who.int/emergencies/disease-outbreak-news/item/2014_3_21polio-en
1857	2014-03-23	https://www.who.int/emergencies/disease-outbreak-news/item/2014_03_23_ebola-en
1858	2014-03-24	https://www.who.int/emergencies/disease-outbreak-news/item/2014_03_24_ebola-en
1859	2014-03-25	https://www.who.int/emergencies/disease-outbreak-news/item/2014_03_25-en
1860	2014-03-25	https://www.who.int/emergencies/disease-outbreak-news/item/2014_03_25_ebola-en
1861	2014-03-26	https://www.who.int/emergencies/disease-outbreak-news/item/2014_03_26-en
1862	2014-03-26	https://www.who.int/emergencies/disease-outbreak-news/item/2014_03_26_ebola-en
1863	2014-03-27	https://www.who.int/emergencies/disease-outbreak-news/item/2014_03_27_ebola-en
1864	2014-03-27	https://www.who.int/emergencies/disease-outbreak-news/item/2014_03_27_mers-en

1865	2014-03-28	https://www.who.int/emergencies/disease-outbreak-news/item/2014_03_28-en
1866	2014-03-30	https://www.who.int/emergencies/disease-outbreak-news/item/2014_03_30_ebola_lbr-en
1867	2014-04-01	https://www.who.int/emergencies/disease-outbreak-news/item/2014_04_01_ebola-en
1868	2014-04-02	https://www.who.int/emergencies/disease-outbreak-news/item/2014_04_02_ebola-en
1869	2014-04-03	https://www.who.int/emergencies/disease-outbreak-news/item/2014_04_03-en
1870	2014-04-03	https://www.who.int/emergencies/disease-outbreak-news/item/2014_04_ebola-en
1871	2014-04-04	https://www.who.int/emergencies/disease-outbreak-news/item/2014_04_04-en
1872	2014-04-05	https://www.who.int/emergencies/disease-outbreak-news/item/2014_04_05_ebola-en
1873	2014-04-07	https://www.who.int/emergencies/disease-outbreak-news/item/2014_04_07_ebola-en
1874	2014-04-08	https://www.who.int/emergencies/disease-outbreak-news/item/2014_04_08-en
1875	2014-04-08	https://www.who.int/emergencies/disease-outbreak-news/item/2014_04_08_HKSAR-en
1876	2014-04-10	https://www.who.int/emergencies/disease-outbreak-news/item/2014_04_10_ebola-en
1877	2014-04-10	https://www.who.int/emergencies/disease-outbreak-news/item/2014_04_10_h7n9-en
1878	2014-04-10	https://www.who.int/emergencies/disease-outbreak-news/item/2014_04_10_mers-en
1879	2014-04-11	https://www.who.int/emergencies/disease-outbreak-news/item/2014_04_11_h7n9-en
1880	2014-04-11	https://www.who.int/emergencies/disease-outbreak-news/item/2014_04_11_mers-en
1881	2014-04-14	https://www.who.int/emergencies/disease-outbreak-news/item/2014_04_14_h7n9-en
1882	2014-04-14	https://www.who.int/emergencies/disease-outbreak-news/item/2014_04_14_mers-en
1883	2014-04-14	https://www.who.int/emergencies/disease-outbreak-news/item/2014_04_14_ebola-en
1884	2014-04-15	https://www.who.int/emergencies/disease-outbreak-news/item/2014_04_15_h7n9-en
1885	2014-04-16	https://www.who.int/emergencies/disease-outbreak-news/item/2014_04_16_h7n9-en
1886	2014-04-16	https://www.who.int/emergencies/disease-outbreak-news/item/2014_04_16_mers-en
1887	2014-04-17	https://www.who.int/emergencies/disease-outbreak-news/item/2014_04_17_mers-en
1888	2014-04-17	https://www.who.int/emergencies/disease-outbreak-news/item/2014_4_17polio-en
1889	2014-04-17	https://www.who.int/emergencies/disease-outbreak-news/item/2014_04_17_ebola-en
1890	2014-04-19	https://www.who.int/emergencies/disease-outbreak-news/item/2014_04_19_ebola-en
1891	2014-04-20	https://www.who.int/emergencies/disease-outbreak-news/item/2014_04_20_mers-en
1892	2014-04-22	https://www.who.int/emergencies/disease-outbreak-news/item/2014_04_22_ebola-en
1893	2014-04-23	https://www.who.int/emergencies/disease-outbreak-news/item/2014_04_23_mers-en
1894	2014-04-24	https://www.who.int/emergencies/disease-outbreak-news/item/2014_04_24_h7n9-en
1895	2014-04-24	https://www.who.int/emergencies/disease-outbreak-news/item/2014_04_24_mers-en
1896	2014-04-24	https://www.who.int/emergencies/disease-outbreak-news/item/2014_04_24_yellowfever-en
1897	2014-04-25	https://www.who.int/emergencies/disease-outbreak-news/item/2014_04_25_ebola-en
1898	2014-04-26	https://www.who.int/emergencies/disease-outbreak-news/item/2014_04_26_mers-en
1899	2014-04-28	https://www.who.int/emergencies/disease-outbreak-news/item/2014_04_28_ebola-en
1900	2014-05-01	https://www.who.int/emergencies/disease-outbreak-news/item/2014_05_01_h7n9-en
1901	2014-05-01	https://www.who.int/emergencies/disease-outbreak-news/item/2014_05_01_mers-en
1902	2014-05-02	https://www.who.int/emergencies/disease-outbreak-news/item/2014_05_02_ebola-en
1903	2014-05-05	https://www.who.int/emergencies/disease-outbreak-news/item/2014_05_05_mers-en

1904	2014-05-05	https://www.who.int/emergencies/disease-outbreak-news/item/2014_05_05_mers_jordan-en
1905	2014-05-06	https://www.who.int/emergencies/disease-outbreak-news/item/2014_05_06_ebola-en
1906	2014-05-07	https://www.who.int/emergencies/disease-outbreak-news/item/2014_05_07_mers_jordan-en
1907	2014-05-07	https://www.who.int/emergencies/disease-outbreak-news/item/2014_05_07_mers_yemen-en
1908	2014-05-08	https://www.who.int/emergencies/disease-outbreak-news/item/2014_05_08_ebola-en
1909	2014-05-09	https://www.who.int/emergencies/disease-outbreak-news/item/2014_05_09_ebola-en
1910	2014-05-12	https://www.who.int/emergencies/disease-outbreak-news/item/2014_05_12_ebola-en
1911	2014-05-14	https://www.who.int/emergencies/disease-outbreak-news/item/2014_05_14_mers-en
1912	2014-05-15	https://www.who.int/emergencies/disease-outbreak-news/item/2014_05_15_ebola-en
1913	2014-05-15	https://www.who.int/emergencies/disease-outbreak-news/item/2014_05_15_h7n9-en
1914	2014-05-15	https://www.who.int/emergencies/disease-outbreak-news/item/2014_05_15_mers-en
1915	2014-05-16	https://www.who.int/emergencies/disease-outbreak-news/item/2014_05_16_mers-en
1916	2014-05-22	https://www.who.int/emergencies/disease-outbreak-news/item/2014_05_22_h7n9-en
1917	2014-05-22	https://www.who.int/emergencies/disease-outbreak-news/item/2014_05_22_mers-en
1918	2014-05-23	https://www.who.int/emergencies/disease-outbreak-news/item/2014_05_23_mers-en
1919	2014-05-24	https://www.who.int/emergencies/disease-outbreak-news/item/2014_05_24_ebola-en
1920	2014-05-28	https://www.who.int/emergencies/disease-outbreak-news/item/2014_05_28_h7n9-en
1921	2014-05-28	https://www.who.int/emergencies/disease-outbreak-news/item/2014_05_28_mers-en
1922	2014-05-28	https://www.who.int/emergencies/disease-outbreak-news/item/2014_05_28_ebola-en
1923	2014-05-30	https://www.who.int/emergencies/disease-outbreak-news/item/2014_05_30_ebola-en
1924	2014-06-04	https://www.who.int/emergencies/disease-outbreak-news/item/2014_06_04_ebola-en
1925	2014-06-04	https://www.who.int/emergencies/disease-outbreak-news/item/2014_06_04_mers-en
1926	2014-06-06	https://www.who.int/emergencies/disease-outbreak-news/item/2014_06_06_ebola-en
1927	2014-06-10	https://www.who.int/emergencies/disease-outbreak-news/item/2014_06_10_ebola-en
1928	2014-06-10	https://www.who.int/emergencies/disease-outbreak-news/item/2014_06_10_h7n9-en
1929	2014-06-11	https://www.who.int/emergencies/disease-outbreak-news/item/2014_06_11_mers-en
1930	2014-06-13	https://www.who.int/emergencies/disease-outbreak-news/item/2014_06_13_mers-en
1931	2014-06-14	https://www.who.int/emergencies/disease-outbreak-news/item/2014_06_14_mers-en
1932	2014-06-16	https://www.who.int/emergencies/disease-outbreak-news/item/2014_06_16_mers-en
1933	2014-06-18	https://www.who.int/emergencies/disease-outbreak-news/item/2014_06_18_avian_influenza-en
1934	2014-06-18	https://www.who.int/emergencies/disease-outbreak-news/item/2014_06_18_ebola-en
1935	2014-06-22	https://www.who.int/emergencies/disease-outbreak-news/item/2014_06_22_ebola-en
1936	2014-06-23	https://www.who.int/emergencies/disease-outbreak-news/item/2014_6_23polio-en
1937	2014-06-24	https://www.who.int/emergencies/disease-outbreak-news/item/2014_06_24_avian_influenza-en
1938	2014-06-24	https://www.who.int/emergencies/disease-outbreak-news/item/2014_06_24_ebola-en
1939	2014-06-25	https://www.who.int/emergencies/disease-outbreak-news/item/2014_06_25_mers-en
1940	2014-06-25	https://www.who.int/emergencies/disease-outbreak-news/item/2014_6_25_polio-en
1941	2014-06-26	https://www.who.int/emergencies/disease-outbreak-news/item/2014_06_26_mers-en
1942	2014-06-27	https://www.who.int/emergencies/disease-outbreak-news/item/2014_06_27_avian_influenza-en

1943	2014-07-01	https://www.who.int/emergencies/disease-outbreak-news/item/2014_07_01_ebola-en
1944	2014-07-02	https://www.who.int/emergencies/disease-outbreak-news/item/2014_07_02_mers-en
1945	2014-07-03	https://www.who.int/emergencies/disease-outbreak-news/item/2014_07_03_ebola-en
1946	2014-07-04	https://www.who.int/emergencies/disease-outbreak-news/item/2014_07_04_mers-en
1947	2014-07-08	https://www.who.int/emergencies/disease-outbreak-news/item/2014_07_08_ebola-en
1948	2014-07-10	https://www.who.int/emergencies/disease-outbreak-news/item/2014_07_10_ebola-en
1949	2014-07-14	https://www.who.int/emergencies/disease-outbreak-news/item/2014_07_14_mers-en
1950	2014-07-14	https://www.who.int/emergencies/disease-outbreak-news/item/2014_07_14_mers2-en
1951	2014-07-15	https://www.who.int/emergencies/disease-outbreak-news/item/2014_07_15_ebola-en
1952	2014-07-17	https://www.who.int/emergencies/disease-outbreak-news/item/2014_07_17_ebola-en
1953	2014-07-17	https://www.who.int/emergencies/disease-outbreak-news/item/2014_07_17_polio-en
1954	2014-07-19	https://www.who.int/emergencies/disease-outbreak-news/item/2014_07_19_ebola-en
1955	2014-07-23	https://www.who.int/emergencies/disease-outbreak-news/item/2014_07_23_mers-en
1956	2014-07-24	https://www.who.int/emergencies/disease-outbreak-news/item/2014_07_24_ebola-en
1957	2014-07-27	https://www.who.int/emergencies/disease-outbreak-news/item/2014_07_27_ebola-en
1958	2014-07-31	https://www.who.int/emergencies/disease-outbreak-news/item/2014_07_31_ebola-en
1959	2014-08-04	https://www.who.int/emergencies/disease-outbreak-news/item/2014_08_04_ebola-en
1960	2014-08-06	https://www.who.int/emergencies/disease-outbreak-news/item/2014_08_06_ebola-en
1961	2014-08-08	https://www.who.int/emergencies/disease-outbreak-news/item/2014_08_08_ebola-en
1962	2014-08-11	https://www.who.int/emergencies/disease-outbreak-news/item/2014_08_11_ebola-en
1963	2014-08-13	https://www.who.int/emergencies/disease-outbreak-news/item/2014_08_13_ebola-en
1964	2014-08-15	https://www.who.int/emergencies/disease-outbreak-news/item/2014_08_15_ebola-en
1965	2014-08-19	https://www.who.int/emergencies/disease-outbreak-news/item/2014_08_19_ebola-en
1966	2014-08-20	https://www.who.int/emergencies/disease-outbreak-news/item/2014_08_20_ebola-en
1967	2014-08-22	https://www.who.int/emergencies/disease-outbreak-news/item/2014_08_22_ebola-en
1968	2014-08-27	https://www.who.int/emergencies/disease-outbreak-news/item/2014_08_27_ebola-en
1969	2014-08-28	https://www.who.int/emergencies/disease-outbreak-news/item/2014_08_28_ebola-en
1970	2014-08-30	https://www.who.int/emergencies/disease-outbreak-news/item/2014_08_30_ebola-en
1971	2014-09-04	https://www.who.int/emergencies/disease-outbreak-news/item/2014_09_04_avian_influenza-en
1972	2014-09-04	https://www.who.int/emergencies/disease-outbreak-news/item/2014_09_04_ebola-en
1973	2014-09-06	https://www.who.int/emergencies/disease-outbreak-news/item/2014_09_06_polio-en
1974	2014-09-10	https://www.who.int/emergencies/disease-outbreak-news/item/2014_09_10_ebola-en
1975	2014-09-17	https://www.who.int/emergencies/disease-outbreak-news/item/17-september-2014-enterovirus-en
1976	2014-10-01	https://www.who.int/emergencies/disease-outbreak-news/item/01-october-2014-ebola-en
1978	2014-10-02	https://www.who.int/emergencies/disease-outbreak-news/item/02-october-2014-mers-austria-en
1977	2014-10-02	https://www.who.int/emergencies/disease-outbreak-news/item/02-october-2014-mers-saudi-arabia-en
1979	2014-10-09	https://www.who.int/emergencies/disease-outbreak-news/item/09-october-2014-ebola-en
1980	2014-10-10	https://www.who.int/emergencies/disease-outbreak-news/item/10-october-2014-marburg-en

1981	2014-10-16	https://www.who.int/emergencies/disease-outbreak-news/item/16-october-2014-mers-en
1982	2014-10-23	https://www.who.int/emergencies/disease-outbreak-news/item/23-october-2014-chikungunya-en
1983	2014-10-24	https://www.who.int/emergencies/disease-outbreak-news/item/24-october-2014-mers-en
1984	2014-10-29	https://www.who.int/emergencies/disease-outbreak-news/item/29-october-2014-avian-influenza-en
1985	2014-10-31	https://www.who.int/emergencies/disease-outbreak-news/item/31-october-2014-ebola-en
1986	2014-10-31	https://www.who.int/emergencies/disease-outbreak-news/item/31-october-2014-mers-en
1987	2014-11-03	https://www.who.int/emergencies/disease-outbreak-news/item/03-november-2014-mers-en
1988	2014-11-07	https://www.who.int/emergencies/disease-outbreak-news/item/07-november-2014-mers-en
1989	2014-11-13	https://www.who.int/emergencies/disease-outbreak-news/item/13-november-2014-legionellosis-en
1990	2014-11-13	https://www.who.int/emergencies/disease-outbreak-news/item/13-november-2014-marburg-en
1991	2014-11-14	https://www.who.int/emergencies/disease-outbreak-news/item/14-november-2014-polio-en
1992	2014-11-18	https://www.who.int/emergencies/disease-outbreak-news/item/18-november-2014-avian-influenza-en
1993	2014-11-21	https://www.who.int/emergencies/disease-outbreak-news/item/21-november-2014-mers-en
1994	2014-11-21	https://www.who.int/emergencies/disease-outbreak-news/item/21-november-2014-plague-en
1995	2014-12-02	https://www.who.int/emergencies/disease-outbreak-news/item/2-december-2014-mers-en
1996	2014-12-15	https://www.who.int/emergencies/disease-outbreak-news/item/15-december-2014-wnv-en
1997	2014-12-17	https://www.who.int/emergencies/disease-outbreak-news/item/17-december-2014-mers-en
1998	2014-12-24	https://www.who.int/emergencies/disease-outbreak-news/item/24-december-2014-avian-influenza-en
1999	2014-12-26	https://www.who.int/emergencies/disease-outbreak-news/item/26-december-2014-mers-en
2000	2014-12-28	https://www.who.int/emergencies/disease-outbreak-news/item/28-december-2014-avian-influenza-en
2001	2014-12-30	https://www.who.int/emergencies/disease-outbreak-news/item/30-december-2014-avian-influenza-en
2002	2014-12-30	https://www.who.int/emergencies/disease-outbreak-news/item/30-december-2014-ebola-en
2003	2015-01-05	https://www.who.int/emergencies/disease-outbreak-news/item/05-january-2015-mers-jordan-en
2004	2015-01-15	https://www.who.int/emergencies/disease-outbreak-news/item/15-january-2015-mers-en
2005	2015-01-16	https://www.who.int/emergencies/disease-outbreak-news/item/16-january-2015-mers-oman-en
2006	2015-01-19	https://www.who.int/emergencies/disease-outbreak-news/item/19-january-2015-avian-influenza-en
2007	2015-01-20	https://www.who.int/emergencies/disease-outbreak-news/item/20-january-2015-mers-en
2008	2015-01-23	https://www.who.int/emergencies/disease-outbreak-news/item/23-january-2015-mers-oman-en
2009	2015-01-27	https://www.who.int/emergencies/disease-outbreak-news/item/27-january-2015-avian-influenza-en
2010	2015-02-01	https://www.who.int/emergencies/disease-outbreak-news/item/01-february-2015-avian-influenza-en
2011	2015-02-03	https://www.who.int/emergencies/disease-outbreak-news/item/03-february-2015-mers-en
2012	2015-02-08	https://www.who.int/emergencies/disease-outbreak-news/item/8-february-2015-avian-influenza-en
2015	2015-02-11	https://www.who.int/emergencies/disease-outbreak-news/item/11-february-2015-mers-are-en
2014	2015-02-11	https://www.who.int/emergencies/disease-outbreak-news/item/11-february-2015-mers-qatar-en
2013	2015-02-11	https://www.who.int/emergencies/disease-outbreak-news/item/11-february-2015-mers-saudi-arabia-en
2016	2015-02-12	https://www.who.int/emergencies/disease-outbreak-news/item/12-february-2015-avian-influenza-en
2017	2015-02-13	https://www.who.int/emergencies/disease-outbreak-news/item/13-february-2015-measles-en

2018	2015-02-13	https://www.who.int/emergencies/disease-outbreak-news/item/13-february-2015-mers-en
2019	2015-02-16	https://www.who.int/emergencies/disease-outbreak-news/item/16-february-2015-mers-saudi-arabia-en
2020	2015-02-23	https://www.who.int/emergencies/disease-outbreak-news/item/23-february-2015-mers-saudi-arabia-en
2021	2015-02-26	https://www.who.int/emergencies/disease-outbreak-news/item/26-february-2015-avian-influenza-en
2022	2015-02-26	https://www.who.int/emergencies/disease-outbreak-news/item/26-february-2015-mers-saudi-arabia-en
2023	2015-03-06	https://www.who.int/emergencies/disease-outbreak-news/item/6-march-2015-measles-en
2024	2015-03-06	https://www.who.int/emergencies/disease-outbreak-news/item/6-march-2015-mers-saudi-arabia-en
2025	2015-03-09	https://www.who.int/emergencies/disease-outbreak-news/item/9-march-2015-mers-germany-en
2026	2015-03-11	https://www.who.int/emergencies/disease-outbreak-news/item/11-march-2015-avian-influenza-china-en
2027	2015-03-11	https://www.who.int/emergencies/disease-outbreak-news/item/11-march-2015-mers-qatar-en
2028	2015-03-11	https://www.who.int/emergencies/disease-outbreak-news/item/11-march-2015-mers-saudi-arabia-en
2029	2015-03-13	https://www.who.int/emergencies/disease-outbreak-news/item/13-march-2015-nigeria-en
2030	2015-03-17	https://www.who.int/emergencies/disease-outbreak-news/item/17-march-2015-uganda-en
2031	2015-03-20	https://www.who.int/emergencies/disease-outbreak-news/item/20-march-2015-mers-saudi-arabia-en
2032	2015-03-26	https://www.who.int/emergencies/disease-outbreak-news/item/26-march-2015-mers-saudi-arabia-en
2033	2015-04-09	https://www.who.int/emergencies/disease-outbreak-news/item/9-april-2015-mers-saudi-arabia-en
2034	2015-04-14	https://www.who.int/emergencies/disease-outbreak-news/item/14-April-2015-avian-influenza-china-en
2035	2015-04-16	https://www.who.int/emergencies/disease-outbreak-news/item/16-april-2015-mers-saudi-arabia-en
2036	2015-04-29	https://www.who.int/emergencies/disease-outbreak-news/item/29-april-2015-mers-saudi-arabia-en
2037	2015-04-29	https://www.who.int/emergencies/disease-outbreak-news/item/29-april-2015-niger-en
2038	2015-05-08	https://www.who.int/emergencies/disease-outbreak-news/item/8-may-2015-mers-iran-en
2039	2015-05-08	https://www.who.int/emergencies/disease-outbreak-news/item/8-may-2015-mers-saudi-arabia-en
2040	2015-05-13	https://www.who.int/emergencies/disease-outbreak-news/item/13-may-2015-ebola-en
2041	2015-05-14	https://www.who.int/emergencies/disease-outbreak-news/item/14-May-2015-avian-influenza-china-en
2042	2015-05-15	https://www.who.int/emergencies/disease-outbreak-news/item/15-may-2015-niger-en
2043	2015-05-17	https://www.who.int/emergencies/disease-outbreak-news/item/17-may-2015-mers-saudi-arabia-en
2044	2015-05-18	https://www.who.int/emergencies/disease-outbreak-news/item/18-may-2015-mers-are-en
2045	2015-05-24	https://www.who.int/emergencies/disease-outbreak-news/item/24-may-2015-mers-are-en
2046	2015-05-24	https://www.who.int/emergencies/disease-outbreak-news/item/24-may-2015-mers-korea-en
2047	2015-05-24	https://www.who.int/emergencies/disease-outbreak-news/item/24-may-2015-mers-qatar-en
2048	2015-05-24	https://www.who.int/emergencies/disease-outbreak-news/item/24-may-2015-mers-saudi-arabia-en
2049	2015-05-25	https://www.who.int/emergencies/disease-outbreak-news/item/25-may-2015-mers-saudi-arabia-en
2050	2015-05-28	https://www.who.int/emergencies/disease-outbreak-news/item/28-may-2015-lassa-fever-usa-en
2051	2015-05-30	https://www.who.int/emergencies/disease-outbreak-news/item/30-may-2015-mers-china-en
2052	2015-05-30	https://www.who.int/emergencies/disease-outbreak-news/item/30-may-2015-mers-korea-en
2053	2015-05-31	https://www.who.int/emergencies/disease-outbreak-news/item/31-may-2015-mers-korea-en

2054	2015-05-31	https://www.who.int/emergencies/disease-outbreak-news/item/31-may-2015-mers-qatar-en
2056	2015-06-01	https://www.who.int/emergencies/disease-outbreak-news/item/01-june-2015-mers-korea-en
2055	2015-06-01	https://www.who.int/emergencies/disease-outbreak-news/item/01-june-2015-mers-saudi-arabia-en
2058	2015-06-04	https://www.who.int/emergencies/disease-outbreak-news/item/04-june-2015-mers-korea-en
2057	2015-06-04	https://www.who.int/emergencies/disease-outbreak-news/item/04-june-2015-mers-oman-en
2059	2015-06-04	https://www.who.int/emergencies/disease-outbreak-news/item/04-june-2015-mers-saudi-arabia-en
2060	2015-06-05	https://www.who.int/emergencies/disease-outbreak-news/item/05-june-2015-mers-korea-en
2061	2015-06-06	https://www.who.int/emergencies/disease-outbreak-news/item/06-june-2015-mers-korea-en
2062	2015-06-06	https://www.who.int/emergencies/disease-outbreak-news/item/06-june-2015-mers-saudi-arabia-en
2063	2015-06-08	https://www.who.int/emergencies/disease-outbreak-news/item/08-june-2015-mers-korea-en
2064	2015-06-09	https://www.who.int/emergencies/disease-outbreak-news/item/09-june-2015-mers-are-en
2065	2015-06-09	https://www.who.int/emergencies/disease-outbreak-news/item/09-june-2015-mers-korea-en
2066	2015-06-11	https://www.who.int/emergencies/disease-outbreak-news/item/11-june-2015-mers-saudi-arabia-en
2067	2015-06-12	https://www.who.int/emergencies/disease-outbreak-news/item/12-june-2015-measles-en
2068	2015-06-12	https://www.who.int/emergencies/disease-outbreak-news/item/12-june-2015-mers-korea-en
2069	2015-06-15	https://www.who.int/emergencies/disease-outbreak-news/item/15-june-2015-avian-influenza-china-en
2070	2015-06-16	https://www.who.int/emergencies/disease-outbreak-news/item/16-june-2015-mers-are-en
2071	2015-06-16	https://www.who.int/emergencies/disease-outbreak-news/item/16-june-2015-mers-korea-en
2072	2015-06-16	https://www.who.int/emergencies/disease-outbreak-news/item/16-june-2015-mers-saudi-arabia-en
2073	2015-06-19	https://www.who.int/emergencies/disease-outbreak-news/item/19-june-2015-mers-korea-en
2074	2015-06-20	https://www.who.int/emergencies/disease-outbreak-news/item/20-june-2015-mers-thailand-en
2075	2015-06-23	https://www.who.int/emergencies/disease-outbreak-news/item/23-june-2015-mers-korea-en
2076	2015-06-23	https://www.who.int/emergencies/disease-outbreak-news/item/23-june-2015-mers-saudi-arabia-en
2077	2015-06-26	https://www.who.int/emergencies/disease-outbreak-news/item/26-june-2015-mers-are-en
2078	2015-06-26	https://www.who.int/emergencies/disease-outbreak-news/item/26-june-2015-mers-korea-en
2079	2015-06-30	https://www.who.int/emergencies/disease-outbreak-news/item/30-june-2015-mers-korea-en
2080	2015-07-03	https://www.who.int/emergencies/disease-outbreak-news/item/03-july-2015-mers-korea-en
2081	2015-07-03	https://www.who.int/emergencies/disease-outbreak-news/item/03-july-2015-mers-saudi-arabia-en
2082	2015-07-07	https://www.who.int/emergencies/disease-outbreak-news/item/07-july-2015-mers-korea-en
2083	2015-07-08	https://www.who.int/emergencies/disease-outbreak-news/item/08-july-2015-mers-philippines-en
2084	2015-07-10	https://www.who.int/emergencies/disease-outbreak-news/item/10-july-2015-mers-korea-en
2085	2015-07-10	https://www.who.int/emergencies/disease-outbreak-news/item/10-july-2015-mers-philippines-en
2086	2015-07-10	https://www.who.int/emergencies/disease-outbreak-news/item/10-july-2015-mers-thailand-en
2087	2015-07-14	https://www.who.int/emergencies/disease-outbreak-news/item/14-july-2015-avian-influenza-en
2088	2015-07-14	https://www.who.int/emergencies/disease-outbreak-news/item/14-july-2015-mers-korea-en
2089	2015-07-17	https://www.who.int/emergencies/disease-outbreak-news/item/17-july-2015-mers-korea-en
2090	2015-07-18	https://www.who.int/emergencies/disease-outbreak-news/item/18-july-2015-avian-influenza-china-en
2091	2015-07-21	https://www.who.int/emergencies/disease-outbreak-news/item/21-july-2015-mers-korea-en

2092	2015-07-23	https://www.who.int/emergencies/disease-outbreak-news/item/23-july-2015-niger-en
2093	2015-07-24	https://www.who.int/emergencies/disease-outbreak-news/item/24-july-2015-mers-saudi-arabia-en
2094	2015-07-24	https://www.who.int/emergencies/disease-outbreak-news/item/24-july-2015-polio-en
2095	2015-07-29	https://www.who.int/emergencies/disease-outbreak-news/item/29-july-2015-mers-saudi-arabia-en
2096	2015-08-06	https://www.who.int/emergencies/disease-outbreak-news/item/06-august-2015-mers-saudi-arabia-en
2097	2015-08-10	https://www.who.int/emergencies/disease-outbreak-news/item/10-august-2015-chikungunya-en
2098	2015-08-12	https://www.who.int/emergencies/disease-outbreak-news/item/12-august-2015-mers-saudi-arabia-en
2099	2015-08-18	https://www.who.int/emergencies/disease-outbreak-news/item/18-august-2015-mers-saudi-arabia-en
2100	2015-08-21	https://www.who.int/emergencies/disease-outbreak-news/item/21-august-2015-mers-saudi-arabia-en
2101	2015-08-26	https://www.who.int/emergencies/disease-outbreak-news/item/26-august-2015-mers-saudi-arabia-en
2102	2015-08-27	https://www.who.int/emergencies/disease-outbreak-news/item/27-august-2015-mers-saudi-arabia-en
2103	2015-09-01	https://www.who.int/emergencies/disease-outbreak-news/item/01-september-2015-mers-jordan-en
2104	2015-09-01	https://www.who.int/emergencies/disease-outbreak-news/item/01-september-2015-polio-en
2105	2015-09-02	https://www.who.int/emergencies/disease-outbreak-news/item/02-september-2015-mers-saudi-arabia-en
2106	2015-09-06	https://www.who.int/emergencies/disease-outbreak-news/item/06-september-2015-mers-jordan-en
2107	2015-09-06	https://www.who.int/emergencies/disease-outbreak-news/item/06-september-2015-plague-en
2108	2015-09-08	https://www.who.int/emergencies/disease-outbreak-news/item/08-september-2015-mers-saudi-arabia-en
2109	2015-09-09	https://www.who.int/emergencies/disease-outbreak-news/item/09-september-2015-mers-saudi-arabia-en
2110	2015-09-11	https://www.who.int/emergencies/disease-outbreak-news/item/11-september-2015-cholera-en
2111	2015-09-14	https://www.who.int/emergencies/disease-outbreak-news/item/14-september-2015-chikungunya-en
2112	2015-09-17	https://www.who.int/emergencies/disease-outbreak-news/item/17-september-2015-chikungunya-en
2113	2015-09-17	https://www.who.int/emergencies/disease-outbreak-news/item/17-september-2015-mers-saudi-arabia-en
2114	2015-09-17	https://www.who.int/emergencies/disease-outbreak-news/item/17-september-2015-wnv-en
2115	2015-09-18	https://www.who.int/emergencies/disease-outbreak-news/item/18-september-2015-mers-jordan-en
2116	2015-09-23	https://www.who.int/emergencies/disease-outbreak-news/item/23-september-2015-mers-kuwait-en
2117	2015-09-27	https://www.who.int/emergencies/disease-outbreak-news/item/27-september-2015-mers-saudi-arabia-en
2118	2015-09-28	https://www.who.int/emergencies/disease-outbreak-news/item/28-september-2015-cholera-en
2119	2015-09-30	https://www.who.int/emergencies/disease-outbreak-news/item/30-september-2015-mers-saudi-arabia-en
2120	2015-10-01	https://www.who.int/emergencies/disease-outbreak-news/item/01-october-2015-mers-jordan-en
2121	2015-10-12	https://www.who.int/emergencies/disease-outbreak-news/item/12-october-2015-cholera-en
2122	2015-10-12	https://www.who.int/emergencies/disease-outbreak-news/item/12-october-2015-mers-jordan-en
2123	2015-10-12	https://www.who.int/emergencies/disease-outbreak-news/item/12-october-2015-mers-saudi-arabia-en
2124	2015-10-12	https://www.who.int/emergencies/disease-outbreak-news/item/12-october-2015-polio-en
2125	2015-10-19	https://www.who.int/emergencies/disease-outbreak-news/item/19-october-2015-avian-influenza-china-en

2126	2015-10-21	https://www.who.int/emergencies/disease-outbreak-news/item/21-october-2015-cholera-en
2127	2015-10-21	https://www.who.int/emergencies/disease-outbreak-news/item/21-october-2015-zika-en
2128	2015-10-22	https://www.who.int/emergencies/disease-outbreak-news/item/22-october-2015-mers-saudi-arabia-en
2129	2015-10-25	https://www.who.int/emergencies/disease-outbreak-news/item/25-october-2015-mers-korea-en
2130	2015-10-29	https://www.who.int/emergencies/disease-outbreak-news/item/29-october-2015-mers-saudi-arabia-en
2131	2015-11-11	https://www.who.int/emergencies/disease-outbreak-news/item/11-november-2015-zika-en
2132	2015-11-12	https://www.who.int/emergencies/disease-outbreak-news/item/12-november-2015-dengue-en
2133	2015-11-13	https://www.who.int/emergencies/disease-outbreak-news/item/13-november-2015-avian-influenza-china-en
2134	2015-11-13	https://www.who.int/emergencies/disease-outbreak-news/item/13-november-2015-mers-saudi-arabia-en
2135	2015-11-13	https://www.who.int/emergencies/disease-outbreak-news/item/13-november-2015-zika-en
2136	2015-11-16	https://www.who.int/emergencies/disease-outbreak-news/item/16-november-2015-acute-respiratory-syndrome-en
2137	2015-11-20	https://www.who.int/emergencies/disease-outbreak-news/item/20-november-2015-microcephaly-en
2138	2015-11-26	https://www.who.int/emergencies/disease-outbreak-news/item/26-november-2015-cholera-tanzania-en
2139	2015-11-26	https://www.who.int/emergencies/disease-outbreak-news/item/26-november-2015-iraq-cholera-en
2140	2015-11-27	https://www.who.int/emergencies/disease-outbreak-news/item/27-november-2015-microcephaly-en
2141	2015-11-27	https://www.who.int/emergencies/disease-outbreak-news/item/27-november-2015-zika-el-salvador-en
2142	2015-11-27	https://www.who.int/emergencies/disease-outbreak-news/item/27-november-2015-zika-guatemala-en
2143	2015-12-03	https://www.who.int/emergencies/disease-outbreak-news/item/03-december-2015-zika-mexico-en
2144	2015-12-03	https://www.who.int/emergencies/disease-outbreak-news/item/03-december-2015-zika-paraguay-en
2145	2015-12-03	https://www.who.int/emergencies/disease-outbreak-news/item/03-december-2015-zika-venezuela-en
2146	2015-12-04	https://www.who.int/emergencies/disease-outbreak-news/item/4-december-2015-mers-saudi-arabia-en
2147	2015-12-05	https://www.who.int/emergencies/disease-outbreak-news/item/05-december-2015-zika-panama-en
2148	2015-12-15	https://www.who.int/emergencies/disease-outbreak-news/item/15-december-2015-cholera-drc-en
2149	2015-12-15	https://www.who.int/emergencies/disease-outbreak-news/item/15-december-2015-microcephaly-brazil-en
2150	2015-12-15	https://www.who.int/emergencies/disease-outbreak-news/item/15-december-2015-polio-lao-en
2151	2015-12-17	https://www.who.int/emergencies/disease-outbreak-news/item/17-december-2015-avian-influenza-china-en
2152	2015-12-21	https://www.who.int/emergencies/disease-outbreak-news/item/21-december-2015-polio-myanmar-en
2153	2015-12-21	https://www.who.int/emergencies/disease-outbreak-news/item/21-december-2015-zika-cape-verde-en
2154	2015-12-21	https://www.who.int/emergencies/disease-outbreak-news/item/21-december-2015-zika-honduras-en
2155	2015-12-22	https://www.who.int/emergencies/disease-outbreak-news/item/22-december-2015-zika-panama-en
2156	2016-01-04	https://www.who.int/emergencies/disease-outbreak-news/item/4-january-2016-avian-influenza-china-en
2157	2016-01-04	https://www.who.int/emergencies/disease-outbreak-news/item/4-january-2016-mers-saudi-arabia-en
2158	2016-01-07	https://www.who.int/emergencies/disease-outbreak-news/item/7-january-2016-mers-oman-en

2159	2016-01-08	https://www.who.int/emergencies/disease-outbreak-news/item/8-january-2016-brazil-microcephaly-en
2160	2016-01-08	https://www.who.int/emergencies/disease-outbreak-news/item/8-january-2016-zika-france-en
2161	2016-01-08	https://www.who.int/emergencies/disease-outbreak-news/item/8-january-2016-zika-usa-en
2162	2016-01-11	https://www.who.int/emergencies/disease-outbreak-news/item/11-january-2016-avian-influenza-china-en
2163	2016-01-19	https://www.who.int/emergencies/disease-outbreak-news/item/19-january-2016-avian-influenza-china-en
2164	2016-01-20	https://www.who.int/emergencies/disease-outbreak-news/item/20-january-2016-zika-bolivia-en
2165	2016-01-20	https://www.who.int/emergencies/disease-outbreak-news/item/20-january-2016-zika-guyana-barbados-ecuador-en
2166	2016-01-21	https://www.who.int/emergencies/disease-outbreak-news/item/21-january-2016-gbs-el-salvador-en
2167	2016-01-21	https://www.who.int/emergencies/disease-outbreak-news/item/21-january-2016-zika-france-en
2168	2016-01-21	https://www.who.int/emergencies/disease-outbreak-news/item/21-january-2016-zika-haiti-en
2169	2016-01-26	https://www.who.int/emergencies/disease-outbreak-news/item/26-january-2016-avian-influenza-china-en
2170	2016-01-26	https://www.who.int/emergencies/disease-outbreak-news/item/26-january-2016-mers-are-en
2171	2016-01-26	https://www.who.int/emergencies/disease-outbreak-news/item/26-january-2016-mers-saudi-arabia-en
2172	2016-01-27	https://www.who.int/emergencies/disease-outbreak-news/item/27-january-2016-lassa-fever-nigeria-en
2173	2016-01-27	https://www.who.int/emergencies/disease-outbreak-news/item/27-january-2016-zika-dominican-republic-en
2174	2016-01-29	https://www.who.int/emergencies/disease-outbreak-news/item/29-january-2016-mers-thailand-en
2175	2016-01-29	https://www.who.int/emergencies/disease-outbreak-news/item/29-january-2016-polio-lao-en
2176	2016-01-29	https://www.who.int/emergencies/disease-outbreak-news/item/29-january-2016-zika-usa-en
2177	2016-02-02	https://www.who.int/emergencies/disease-outbreak-news/item/2-february-2016-mers-saudi-arabia-en
2178	2016-02-08	https://www.who.int/emergencies/disease-outbreak-news/item/8-february-2016-gbs-brazil-en
2179	2016-02-08	https://www.who.int/emergencies/disease-outbreak-news/item/8-february-2016-gbs-france-martinique-en
2180	2016-02-08	https://www.who.int/emergencies/disease-outbreak-news/item/8-february-2016-zika-americas-region-en
2181	2016-02-08	https://www.who.int/emergencies/disease-outbreak-news/item/8-february-2016-zika-maldives-en
2182	2016-02-10	https://www.who.int/emergencies/disease-outbreak-news/item/10-february-2016-avian-influenza-china-en
2183	2016-02-12	https://www.who.int/emergencies/disease-outbreak-news/item/12-february-2016-gbs-colombia-venezuela-en
2184	2016-02-12	https://www.who.int/emergencies/disease-outbreak-news/item/12-february-2016-microcephaly-usa-en
2185	2016-02-12	https://www.who.int/emergencies/disease-outbreak-news/item/12-february-2016-yellow-fever-angola-en
2186	2016-02-12	https://www.who.int/emergencies/disease-outbreak-news/item/12-february-2016-zika-usa-en
2187	2016-02-19	https://www.who.int/emergencies/disease-outbreak-news/item/19-february-2016-lassa-fever-benin-en
2188	2016-02-22	https://www.who.int/emergencies/disease-outbreak-news/item/22-february-2016-zika-netherlands-en
2189	2016-02-25	https://www.who.int/emergencies/disease-outbreak-news/item/25-february-2016-avian-influenza-china-en
2190	2016-02-25	https://www.who.int/emergencies/disease-outbreak-news/item/25-february-2016-dengue-en
2191	2016-02-25	https://www.who.int/emergencies/disease-outbreak-news/item/25-february-2016-polio-lao-en

2192	2016-02-29	https://www.who.int/emergencies/disease-outbreak-news/item/29-february-2016-mers-saudi-arabia-en
2193	2016-02-29	https://www.who.int/emergencies/disease-outbreak-news/item/29-february-2016-zika-trinidad-and-tobago-en
2194	2016-03-01	https://www.who.int/emergencies/disease-outbreak-news/item/1-march-2016-zika-st-vincent-and-grenadines-en
2195	2016-03-04	https://www.who.int/emergencies/disease-outbreak-news/item/4-march-2016-zika-sint-maarten-en
2196	2016-03-07	https://www.who.int/emergencies/disease-outbreak-news/item/7-march-2016-gbs-french-polynesia-en
2197	2016-03-07	https://www.who.int/emergencies/disease-outbreak-news/item/7-march-2016-zika-argentina-and-france-en
2198	2016-03-10	https://www.who.int/emergencies/disease-outbreak-news/item/10-march-2016-dengue-uruguay-en
2199	2016-03-10	https://www.who.int/emergencies/disease-outbreak-news/item/10-march-2016-mers-qatar-en
2200	2016-03-10	https://www.who.int/emergencies/disease-outbreak-news/item/10-march-2016-mers-saudi-arabia-en
2201	2016-03-14	https://www.who.int/emergencies/disease-outbreak-news/item/14-march-2016-chikungunya-argentina-en
2202	2016-03-14	https://www.who.int/emergencies/disease-outbreak-news/item/14-march-2016-mers-saudi-arabia-en
2203	2016-03-16	https://www.who.int/emergencies/disease-outbreak-news/item/16-march-2016-mers-saudi-arabia-en
2204	2016-03-18	https://www.who.int/emergencies/disease-outbreak-news/item/18-march-2016-mers-saudi-arabia-en
2205	2016-03-21	https://www.who.int/emergencies/disease-outbreak-news/item/21-march-2016-mers-saudi-arabia-en
2206	2016-03-21	https://www.who.int/emergencies/disease-outbreak-news/item/21-march-2016-gbs-usa-en
2207	2016-03-22	https://www.who.int/emergencies/disease-outbreak-news/item/22-march-2016-yellow-fever-angola-en
2208	2016-03-23	https://www.who.int/emergencies/disease-outbreak-news/item/23-march-2016-ah5n6-china-en
2209	2016-03-23	https://www.who.int/emergencies/disease-outbreak-news/item/23-march-2016-avian-ah7n9-china-en
2210	2016-03-23	https://www.who.int/emergencies/disease-outbreak-news/item/23-march-2016-lassa-fever-germany-en
2211	2016-03-23	https://www.who.int/emergencies/disease-outbreak-news/item/23-march-2016-lassa-fever-togo-en
2212	2016-03-23	https://www.who.int/emergencies/disease-outbreak-news/item/23-march-2016-mers-saudi-arabia-en
2213	2016-03-29	https://www.who.int/emergencies/disease-outbreak-news/item/29-march-2016-avian-ah7n9-china-en
2214	2016-03-29	https://www.who.int/emergencies/disease-outbreak-news/item/29-march-2016-gbs-panama-en
2215	2016-03-29	https://www.who.int/emergencies/disease-outbreak-news/item/29-march-2016-microcephaly-panama-en
2216	2016-03-29	https://www.who.int/emergencies/disease-outbreak-news/item/29-march-2016-yellow-fever-china-en
2217	2016-03-29	https://www.who.int/emergencies/disease-outbreak-news/item/29-march-2016-zika-dominica-and-cuba-en
2218	2016-04-01	https://www.who.int/emergencies/disease-outbreak-news/item/1-april-2016-microcephaly-france-en
2219	2016-04-06	https://www.who.int/emergencies/disease-outbreak-news/item/6-april-2016-yellow-fever-china-en
2220	2016-04-06	https://www.who.int/emergencies/disease-outbreak-news/item/6-april-2016-yellow-fever-kenya-en
2221	2016-04-08	https://www.who.int/emergencies/disease-outbreak-news/item/8-april-2016-lassa-fever-sweden-en
2222	2016-04-09	https://www.who.int/emergencies/disease-outbreak-news/item/9-april-2016-microcephaly-france-en
2223	2016-04-11	https://www.who.int/emergencies/disease-outbreak-news/item/11-april-2016-yellow-fever-drc-en
2224	2016-04-12	https://www.who.int/emergencies/disease-outbreak-news/item/12-april-2016-zika-viet-nam-en

2225	2016-04-13	https://www.who.int/emergencies/disease-outbreak-news/item/13-april-2016-yellow-fever-angola-en
2226	2016-04-14	https://www.who.int/emergencies/disease-outbreak-news/item/14-april-2016-mers-saudi-arabia-en
2227	2016-04-15	https://www.who.int/emergencies/disease-outbreak-news/item/15-april-2016-zika-chile-en
2228	2016-04-20	https://www.who.int/emergencies/disease-outbreak-news/item/20-april-2016-zika-saint-lucia-en
2229	2016-04-21	https://www.who.int/emergencies/disease-outbreak-news/item/21-april-2016-elizabethkingia-usa-en
2230	2016-04-21	https://www.who.int/emergencies/disease-outbreak-news/item/21-april-2016-zika-peru-en
2231	2016-04-22	https://www.who.int/emergencies/disease-outbreak-news/item/22-april-2016-mers-saudi-arabia-en
2232	2016-04-22	https://www.who.int/emergencies/disease-outbreak-news/item/22-april-2016-yellow-fever-china-en
2233	2016-04-22	https://www.who.int/emergencies/disease-outbreak-news/item/22-april-2016-zika-png-en
2234	2016-04-25	https://www.who.int/emergencies/disease-outbreak-news/item/25-april-2016-mers-bahrain-en
2235	2016-04-26	https://www.who.int/emergencies/disease-outbreak-news/item/26-april-2016-avian-ah7n9-china-en
2236	2016-04-26	https://www.who.int/emergencies/disease-outbreak-news/item/26-april-2016-mers-saudi-arabia-en
2237	2016-04-27	https://www.who.int/emergencies/disease-outbreak-news/item/27-april-2016-lassa-fever-germany-en
2238	2016-04-28	https://www.who.int/emergencies/disease-outbreak-news/item/28-april-2016-salmonellosis-usa-en
2239	2016-05-02	https://www.who.int/emergencies/disease-outbreak-news/item/02-may-2016-yellow-fever-drc-en
2240	2016-05-02	https://www.who.int/emergencies/disease-outbreak-news/item/02-may-2016-yellow-fever-uganda-en
2241	2016-05-03	https://www.who.int/emergencies/disease-outbreak-news/item/3-may-2016-ah7n9-china-en
2242	2016-05-06	https://www.who.int/emergencies/disease-outbreak-news/item/6-may-2016-ah5n6-china-en
2243	2016-05-10	https://www.who.int/emergencies/disease-outbreak-news/item/10-may-2016-ah5n6-china-en
2244	2016-05-16	https://www.who.int/emergencies/disease-outbreak-news/item/16-may-2016-mers-qatar-en
2245	2016-05-16	https://www.who.int/emergencies/disease-outbreak-news/item/16-may-2016-mers-saudi-arabia-en
2246	2016-05-17	https://www.who.int/emergencies/disease-outbreak-news/item/17-may-2016-ah7n9-china-en
2247	2016-05-18	https://www.who.int/emergencies/disease-outbreak-news/item/18-may-2016-lassa-fever-liberia-en
2248	2016-05-19	https://www.who.int/emergencies/disease-outbreak-news/item/19-may-2016-hf-south-sudan-en
2249	2016-05-27	https://www.who.int/emergencies/disease-outbreak-news/item/27-may-2016-lassa-fever-nigeria-en
2250	2016-06-02	https://www.who.int/emergencies/disease-outbreak-news/item/02-june-2016-yellow-fever-drc-en
2251	2016-06-03	https://www.who.int/emergencies/disease-outbreak-news/item/03-june-2016-oropouche-peru-en
2252	2016-06-08	https://www.who.int/emergencies/disease-outbreak-news/item/08-june-2016-ah5n6-china-en
2253	2016-06-13	https://www.who.int/emergencies/disease-outbreak-news/item/13-june-2016-lassa-fever-benin-en
2254	2016-06-14	https://www.who.int/emergencies/disease-outbreak-news/item/14-june-2016-chikungunya-usa-en
2255	2016-06-14	https://www.who.int/emergencies/disease-outbreak-news/item/14-june-2016-yellow-fever-angola-en
2256	2016-06-19	https://www.who.int/emergencies/disease-outbreak-news/item/19-june-2016-mers-saudi-arabia-en
2257	2016-06-21	https://www.who.int/emergencies/disease-outbreak-news/item/21-june-2016-mers-saudi-arabia-en
2258	2016-06-21	https://www.who.int/emergencies/disease-outbreak-news/item/21-june-2016-mers-uae-en
2259	2016-06-22	https://www.who.int/emergencies/disease-outbreak-news/item/22-june-2016-mers-saudi-arabia-en
2260	2016-06-29	https://www.who.int/emergencies/disease-outbreak-news/item/29-june-2016-mers-qatar-en
2261	2016-07-01	https://www.who.int/emergencies/disease-outbreak-news/item/01-july-2016-ah7n9-china-en

2262	2016-07-06	https://www.who.int/emergencies/disease-outbreak-news/item/06-july-2016-mers-saudi-arabia-en
2263	2016-07-20	https://www.who.int/emergencies/disease-outbreak-news/item/20-july-2016-ehec-uk-en
2264	2016-07-22	https://www.who.int/emergencies/disease-outbreak-news/item/22-july-2016-ah7n9-china-en
2265	2016-07-25	https://www.who.int/emergencies/disease-outbreak-news/item/25-july-2016-mers-saudi-arabia-en
2266	2016-08-02	https://www.who.int/emergencies/disease-outbreak-news/item/02-august-2016-rift-valley-fever-china-en
2267	2016-08-09	https://www.who.int/emergencies/disease-outbreak-news/item/09-august-2016-chikungunya-kenya-en
2268	2016-08-17	https://www.who.int/emergencies/disease-outbreak-news/item/17-august-2016-ah7n9-china-en
2269	2016-08-26	https://www.who.int/emergencies/disease-outbreak-news/item/26-august-2016-mers-thailand-en
2270	2016-09-16	https://www.who.int/emergencies/disease-outbreak-news/item/16-september-2016-mers-saudi-arabia-en
2271	2016-09-20	https://www.who.int/emergencies/disease-outbreak-news/item/20-september-2016-mers-austria-en
2272	2016-09-21	https://www.who.int/emergencies/disease-outbreak-news/item/21-september-2016-mers-saudi-arabia-en
2273	2016-09-29	https://www.who.int/emergencies/disease-outbreak-news/item/29-september-2016-rift-valley-fever-niger-en
2274	2016-10-06	https://www.who.int/emergencies/disease-outbreak-news/item/06-october-2016-polio-nigeria-en
2275	2016-10-13	https://www.who.int/emergencies/disease-outbreak-news/item/13-october-2016-monkeypox-caf-en
2276	2016-10-31	https://www.who.int/emergencies/disease-outbreak-news/item/31-october-2016-mers-saudi-arabia-en
2277	2016-11-11	https://www.who.int/emergencies/disease-outbreak-news/item/11-november-2016-mers-saudi-arabia-en
2278	2016-11-17	https://www.who.int/emergencies/disease-outbreak-news/item/17-november-2016-ah7n9-china-en
2279	2016-11-18	https://www.who.int/emergencies/disease-outbreak-news/item/18-november-2016-dengue-burkina-faso-en
2280	2016-11-24	https://www.who.int/emergencies/disease-outbreak-news/item/24-november-2016-rift-valley-fever-niger-en
2281	2016-11-28	https://www.who.int/emergencies/disease-outbreak-news/item/28-november-2016-mers-saudi-arabia-en
2282	2016-12-05	https://www.who.int/emergencies/disease-outbreak-news/item/5-december-2016-mers-saudi-arabia-en
2283	2016-12-07	https://www.who.int/emergencies/disease-outbreak-news/item/07-december-2016-ah5n6-china-en
2284	2016-12-08	https://www.who.int/emergencies/disease-outbreak-news/item/8-december-2016-mers-oman-en
2285	2016-12-19	https://www.who.int/emergencies/disease-outbreak-news/item/19-december-2016-1-ah7n9-china-en
2286	2016-12-19	https://www.who.int/emergencies/disease-outbreak-news/item/19-december-2016-1-mers-saudi-arabia-en
2287	2016-12-19	https://www.who.int/emergencies/disease-outbreak-news/item/19-december-2016-2-ah7n9-china-en
2288	2016-12-19	https://www.who.int/emergencies/disease-outbreak-news/item/19-december-2016-2-mers-saudi-arabia-en
2289	2016-12-23	https://www.who.int/emergencies/disease-outbreak-news/item/23-december-2016-ah7n9-china-en
2290	2016-12-27	https://www.who.int/emergencies/disease-outbreak-news/item/27-december-2016-polio-pakistan-en
2291	2017-01-03	https://www.who.int/emergencies/disease-outbreak-news/item/03-january-2017-ah7n9-china-en
2292	2017-01-09	https://www.who.int/emergencies/disease-outbreak-news/item/09-january-2017-plague-mdg-en
2293	2017-01-13	https://www.who.int/emergencies/disease-outbreak-news/item/13-january-2017-yellow-fever-brazil-en
2294	2017-01-17	https://www.who.int/emergencies/disease-outbreak-news/item/17-january-2017-ah7n9-china-en

2295	2017-01-17	https://www.who.int/emergencies/disease-outbreak-news/item/17-january-2017-mers-saudi-arabia-en
2296	2017-01-18	https://www.who.int/emergencies/disease-outbreak-news/item/18-january-2017-ah7n9-china-en
2297	2017-01-24	https://www.who.int/emergencies/disease-outbreak-news/item/24-january-2017-hepatitis-e-chad-en
2298	2017-01-26	https://www.who.int/emergencies/disease-outbreak-news/item/26-january-2017-mers-saudi-arabia-en
2299	2017-01-27	https://www.who.int/emergencies/disease-outbreak-news/item/27-january-2017-yellow-fever-brazil-en
2300	2017-02-10	https://www.who.int/emergencies/disease-outbreak-news/item/10-february-2017-mers-saudi-arabia-en
2301	2017-02-20	https://www.who.int/emergencies/disease-outbreak-news/item/20-february-2017-ah7n9-china-en
2302	2017-02-20	https://www.who.int/emergencies/disease-outbreak-news/item/20-february-2017-seoulvirus-usa-and-canada-en
2303	2017-02-22	https://www.who.int/emergencies/disease-outbreak-news/item/22-february-2017-ah7n9-china-en
2304	2017-02-23	https://www.who.int/emergencies/disease-outbreak-news/item/23-february-2017-meningococcal-disease-togo-en
2305	2017-02-24	https://www.who.int/emergencies/disease-outbreak-news/item/24-february-2017-yellow-fever-brazil-en
2306	2017-02-27	https://www.who.int/emergencies/disease-outbreak-news/item/27-february-2017-ah7n9-china-en
2307	2017-03-06	https://www.who.int/emergencies/disease-outbreak-news/item/06-march-2017-yellow-fever-brazil-en
2308	2017-03-10	https://www.who.int/emergencies/disease-outbreak-news/item/10-march-2017-lassa-fever-benin-togo-burkina-faso-en
2309	2017-03-10	https://www.who.int/emergencies/disease-outbreak-news/item/10-march-2017-mers-saudi-arabia-en
2310	2017-03-15	https://www.who.int/emergencies/disease-outbreak-news/item/15-march-2017-ah7n9-china-en
2311	2017-03-16	https://www.who.int/emergencies/disease-outbreak-news/item/16-march-2017-ah7n9-china-en
2312	2017-03-20	https://www.who.int/emergencies/disease-outbreak-news/item/20-march-2017-yellow-fever-brazil-en
2313	2017-03-23	https://www.who.int/emergencies/disease-outbreak-news/item/23-march-2017-ah7n9-china-en
2314	2017-03-24	https://www.who.int/emergencies/disease-outbreak-news/item/24-march-2017-meningococcal-disease-nigeria-en
2315	2017-03-28	https://www.who.int/emergencies/disease-outbreak-news/item/28-march-2017-yellow-fever-suriname-en
2316	2017-04-03	https://www.who.int/emergencies/disease-outbreak-news/item/03-april-2017-ah7n9-china-en
2317	2017-04-03	https://www.who.int/emergencies/disease-outbreak-news/item/03-april-2017-mers-saudi-arabia-en
2318	2017-04-04	https://www.who.int/emergencies/disease-outbreak-news/item/04-april-2017-mers-qatar-en
2319	2017-04-04	https://www.who.int/emergencies/disease-outbreak-news/item/04-april-2017-yellow-fever-brazil-en
2320	2017-04-05	https://www.who.int/emergencies/disease-outbreak-news/item/05-april-2017-ah7n9-china-en
2321	2017-04-18	https://www.who.int/emergencies/disease-outbreak-news/item/18-april-2017-ah7n9-china-en
2322	2017-04-20	https://www.who.int/emergencies/disease-outbreak-news/item/20-april-2017-ah7n9-china-en
2323	2017-04-24	https://www.who.int/emergencies/disease-outbreak-news/item/24-april-2017-mers-uae-en
2324	2017-04-27	https://www.who.int/emergencies/disease-outbreak-news/item/27-april-2017-mers-saudi-arabia-and-qatar-en
2325	2017-05-01	https://www.who.int/emergencies/disease-outbreak-news/item/01-may-2017-ah7n9-china-en
2326	2017-05-05	https://www.who.int/emergencies/disease-outbreak-news/item/05-may-2017-hepatitis-e-niger-en
2327	2017-05-05	https://www.who.int/emergencies/disease-outbreak-news/item/05-may-2017-unexplained-cluster-of-deaths-liberia-en
2328	2017-05-09	https://www.who.int/emergencies/disease-outbreak-news/item/09-may-2017-ah7n9-china-en

2329	2017-05-13	https://www.who.int/emergencies/disease-outbreak-news/item/13-may-2017-ebola-drc-en
2330	2017-05-18	https://www.who.int/emergencies/disease-outbreak-news/item/18-may-2017-ah7n9-china-en
2331	2017-05-23	https://www.who.int/emergencies/disease-outbreak-news/item/23-may-2017-ah7n9-china-en
2332	2017-05-26	https://www.who.int/emergencies/disease-outbreak-news/item/26-may-2017-zika-ind-en
2333	2017-06-06	https://www.who.int/emergencies/disease-outbreak-news/item/06-june-2017-mers-en
2334	2017-06-07	https://www.who.int/emergencies/disease-outbreak-news/item/07-june-2017-hepatitis-a-en
2335	2017-06-08	https://www.who.int/emergencies/disease-outbreak-news/item/08-june-2017-ah7n9-china-en
2336	2017-06-13	https://www.who.int/emergencies/disease-outbreak-news/item/13-june-2017-mers-saudi-arabia-en
2337	2017-06-13	https://www.who.int/emergencies/disease-outbreak-news/item/13-June-2017-polio-drc-en
2338	2017-06-13	https://www.who.int/emergencies/disease-outbreak-news/item/13-June-2017-polio-syrian-arab-republic-en
2339	2017-06-19	https://www.who.int/emergencies/disease-outbreak-news/item/19-june-2017-mers-saudi-arabia-en
2340	2017-06-28	https://www.who.int/emergencies/disease-outbreak-news/item/28-june-2017-ah7n9-china-en
2341	2017-06-28	https://www.who.int/emergencies/disease-outbreak-news/item/28-june-2017-lassa-fever-nigeria-en
2342	2017-06-28	https://www.who.int/emergencies/disease-outbreak-news/item/28-june-2017-mers-saudi-arabia-en
2343	2017-07-04	https://www.who.int/emergencies/disease-outbreak-news/item/04-july-2017-mers-lebanon-en
2344	2017-07-06	https://www.who.int/emergencies/disease-outbreak-news/item/06-july-2017-meningococcal-septicaemia-liberia-en
2345	2017-07-06	https://www.who.int/emergencies/disease-outbreak-news/item/06-july-2017-mers-saudi-arabia-en
2346	2017-07-12	https://www.who.int/emergencies/disease-outbreak-news/item/12-july-2017-cholera-nigeria-en
2347	2017-07-12	https://www.who.int/emergencies/disease-outbreak-news/item/12-july-2017-hepatitis-e-nigeria-en
2348	2017-07-19	https://www.who.int/emergencies/disease-outbreak-news/item/19-july-2017-ah7n9-china-en
2349	2017-07-19	https://www.who.int/emergencies/disease-outbreak-news/item/19-july-2017-dengue-sri-lanka-en
2350	2017-07-21	https://www.who.int/emergencies/disease-outbreak-news/item/21-july-2017-cholera-kenya-en
2351	2017-08-04	https://www.who.int/emergencies/disease-outbreak-news/item/04-august-2017-dengue-cote-d-ivoire-en
2352	2017-08-07	https://www.who.int/emergencies/disease-outbreak-news/item/07-august-2017-ah7n9-china-en
2353	2017-08-17	https://www.who.int/emergencies/disease-outbreak-news/item/17-august-2017-mers-saudi-arabia-en
2354	2017-08-25	https://www.who.int/emergencies/disease-outbreak-news/item/25-august-2017-chikungunya-france-en
2355	2017-08-28	https://www.who.int/emergencies/disease-outbreak-news/item/28-august-2017-mers-uae-en
2356	2017-08-30	https://www.who.int/emergencies/disease-outbreak-news/item/30-august-2017-yellow-fever-french-guiana-en
2357	2017-09-05	https://www.who.int/emergencies/disease-outbreak-news/item/5-september-2017-ah7n9-china-en
2358	2017-09-06	https://www.who.int/emergencies/disease-outbreak-news/item/6-september-2017-mers-saudi-arabia-en
2359	2017-09-12	https://www.who.int/emergencies/disease-outbreak-news/item/12-september-2017-mers-oman-en
2360	2017-09-13	https://www.who.int/emergencies/disease-outbreak-news/item/13-september-2017-ah7n9-china-en
2361	2017-09-15	https://www.who.int/emergencies/disease-outbreak-news/item/15-september-2017-chikungunya-italy-en
2362	2017-09-21	https://www.who.int/emergencies/disease-outbreak-news/item/21-september-2017-mers-uae-en
2363	2017-09-29	https://www.who.int/emergencies/disease-outbreak-news/item/29-september-2017-chikungunya-italy-en
2364	2017-09-29	https://www.who.int/emergencies/disease-outbreak-news/item/29-september-2017-plague-madagascar-en

2365	2017-10-02	https://www.who.int/emergencies/disease-outbreak-news/item/02-october-2017-plague-madagascar-en
2366	2017-10-09	https://www.who.int/emergencies/disease-outbreak-news/item/09-october-2017-mers-saudi-arabia-en
2367	2017-10-15	https://www.who.int/emergencies/disease-outbreak-news/item/15-october-2017-plague-seychelles-en
2368	2017-10-25	https://www.who.int/emergencies/disease-outbreak-news/item/25-october-2017-marburg-uganda-en
2369	2017-10-26	https://www.who.int/emergencies/disease-outbreak-news/item/26-october-2017-ah7n9-china-en
2370	2017-10-26	https://www.who.int/emergencies/disease-outbreak-news/item/26-october-2017-plague-seychelles-en
2371	2017-11-02	https://www.who.int/emergencies/disease-outbreak-news/item/02-november-2017-plague-madagascar-en
2372	2017-11-06	https://www.who.int/emergencies/disease-outbreak-news/item/6-november-2017-dengue-burkina-faso-en
2373	2017-11-07	https://www.who.int/emergencies/disease-outbreak-news/item/7-november-2017-marburg-en
2374	2017-11-07	https://www.who.int/emergencies/disease-outbreak-news/item/7-november-2017-mers-saudi-arabia-en
2375	2017-11-10	https://www.who.int/emergencies/disease-outbreak-news/item/10-november-2017-mers-oman-en
2376	2017-11-15	https://www.who.int/emergencies/disease-outbreak-news/item/15-november-2017-marburg-uganda-kenya-en
2377	2017-11-15	https://www.who.int/emergencies/disease-outbreak-news/item/15-november-2017-plague-madagascar-en
2378	2017-11-24	https://www.who.int/emergencies/disease-outbreak-news/item/24-november-2017-yellow-fever-brazil-en
2379	2017-11-27	https://www.who.int/emergencies/disease-outbreak-news/item/27-november-2017-plague-madagascar-en
2380	2017-12-11	https://www.who.int/emergencies/disease-outbreak-news/item/11-december-2017-cholera-kenya-en
2381	2017-12-11	https://www.who.int/emergencies/disease-outbreak-news/item/11-december-2017-cholera-zambia-en
2382	2017-12-13	https://www.who.int/emergencies/disease-outbreak-news/item/13-december-2017-diphtheria-bangladesh-en
2383	2017-12-19	https://www.who.int/emergencies/disease-outbreak-news/item/19-december-2017-mers-saudi-arabia-en
2384	2017-12-21	https://www.who.int/emergencies/disease-outbreak-news/item/21-december-2017-monkeypox-nigeria-en
2385	2017-12-22	https://www.who.int/emergencies/disease-outbreak-news/item/22-december-2017-diphtheria-yemen-en
2386	2017-12-22	https://www.who.int/emergencies/disease-outbreak-news/item/22-december-2017-mers-cov-united-arab-emirates-en
2387	2017-12-22	https://www.who.int/emergencies/disease-outbreak-news/item/22-december-2017-salmonella-agona-infections-france-en
2388	2017-12-22	https://www.who.int/emergencies/disease-outbreak-news/item/22-december-2017-yellow-fever-nigeria-en
2389	2018-01-08	https://www.who.int/emergencies/disease-outbreak-news/item/08-january-2018-mers-cov-malaysia-en
2390	2018-01-12	https://www.who.int/emergencies/disease-outbreak-news/item/12-january-2018-cholera-tanzania-en
2391	2018-01-15	https://www.who.int/emergencies/disease-outbreak-news/item/15-january-2018-hepatitis-e-namibia-en
2392	2018-01-22	https://www.who.int/emergencies/disease-outbreak-news/item/22-january-2018-yellow-fever-brazil-en
2393	2018-01-26	https://www.who.int/emergencies/disease-outbreak-news/item/26-january-2018-mers-saudi-arabia-en
2394	2018-02-19	https://www.who.int/emergencies/disease-outbreak-news/item/19-february-2018-cholera-mozambique-en
2395	2018-02-22	https://www.who.int/emergencies/disease-outbreak-news/item/22-february-2018-ah7n4-china-en

2396	2018-02-22	https://www.who.int/emergencies/disease-outbreak-news/item/22-february-2018-lassa-fever-liberia-en
2397	2018-02-26	https://www.who.int/emergencies/disease-outbreak-news/item/26-february-2018-rift-valley-fever-gambia-en
2398	2018-02-27	https://www.who.int/emergencies/disease-outbreak-news/item/27-february-2018-chikungunya-kenya-en
2399	2018-02-27	https://www.who.int/emergencies/disease-outbreak-news/item/27-february-2018-yellow-fever-brazil-en
2400	2018-03-01	https://www.who.int/emergencies/disease-outbreak-news/item/01-march-2018-lassa-fever-nigeria-en
2401	2018-03-02	https://www.who.int/emergencies/disease-outbreak-news/item/02-march-2018-cholera-drc-en
2402	2018-03-09	https://www.who.int/emergencies/disease-outbreak-news/item/09-march-2018-yellow-fever-brazil-en
2403	2018-03-09	https://www.who.int/emergencies/disease-outbreak-news/item/2018-DON23
2404	2018-03-15	https://www.who.int/emergencies/disease-outbreak-news/item/15-march-2018-mers-oman-en
2405	2018-03-23	https://www.who.int/emergencies/disease-outbreak-news/item/23-march-2018-lassa-fever-nigeria-en
2406	2018-03-23	https://www.who.int/emergencies/disease-outbreak-news/item/23-march-2018-seasonal-reassortant-ah1n2-netherlands-en
2407	2018-03-28	https://www.who.int/emergencies/disease-outbreak-news/item/28-march-2018-listeriosis-south-africa-en
2408	2018-03-29	https://www.who.int/emergencies/disease-outbreak-news/item/29-march-2018-cholera-somalia-en
2409	2018-04-09	https://www.who.int/emergencies/disease-outbreak-news/item/09-april-2018-listeriosis-australia-en
2410	2018-04-20	https://www.who.int/emergencies/disease-outbreak-news/item/20-april-2018-lassa-fever-nigeria-en
2411	2018-05-01	https://www.who.int/emergencies/disease-outbreak-news/item/01-may-2018-dengue-reunion-en
2412	2018-05-02	https://www.who.int/emergencies/disease-outbreak-news/item/02-may-2018-listeriosis-south-africa-en
2413	2018-05-10	https://www.who.int/emergencies/disease-outbreak-news/item/10-may-2018-ebola-drc-en
2414	2018-05-14	https://www.who.int/emergencies/disease-outbreak-news/item/14-may-2018-ebola-drc-en
2415	2018-05-17	https://www.who.int/emergencies/disease-outbreak-news/item/17-may-2018-ebola-drc-en
2416	2018-05-17	https://www.who.int/emergencies/disease-outbreak-news/item/17-May-2018-polio-somalia-kenya-en
2417	2018-05-21	https://www.who.int/emergencies/disease-outbreak-news/item/21-may-2018-ebola-drc-en
2418	2018-05-23	https://www.who.int/emergencies/disease-outbreak-news/item/23-may-2018-ebola-drc-en
2419	2018-05-28	https://www.who.int/emergencies/disease-outbreak-news/item/28-may-2018-mers-uae-en
2420	2018-05-30	https://www.who.int/emergencies/disease-outbreak-news/item/30-may-2018-ebola-drc-en
2421	2018-05-31	https://www.who.int/emergencies/disease-outbreak-news/item/31-may-2018-nipah-virus-india-en
2422	2018-06-05	https://www.who.int/emergencies/disease-outbreak-news/item/05-june-2018-ebola-drc-en
2423	2018-06-05	https://www.who.int/emergencies/disease-outbreak-news/item/05-june-2018-monkeypox-cameroon-en
2424	2018-06-06	https://www.who.int/emergencies/disease-outbreak-news/item/06-june-2018-ebola-drc-en
2425	2018-06-11	https://www.who.int/emergencies/disease-outbreak-news/item/11-june-2018-ebola-en
2426	2018-06-11	https://www.who.int/emergencies/disease-outbreak-news/item/11-june-2018-measles-brazil-en
2427	2018-06-13	https://www.who.int/emergencies/disease-outbreak-news/item/13-june-2018-ebola-drc-en
2428	2018-06-14	https://www.who.int/emergencies/disease-outbreak-news/item/14-june-2018-cholera-cameroon-en
2429	2018-06-18	https://www.who.int/emergencies/disease-outbreak-news/item/18-june-2018-mers-saudi-arabia-en

2430	2018-06-18	https://www.who.int/emergencies/disease-outbreak-news/item/18-june-2018-rift-valley-fever-kenya-en
2431	2018-06-20	https://www.who.int/emergencies/disease-outbreak-news/item/20-june-2018-ebola-drc-en
2432	2018-06-20	https://www.who.int/emergencies/disease-outbreak-news/item/20-june-2018-measles-japan-en
2433	2018-07-02	https://www.who.int/emergencies/disease-outbreak-news/item/02-July-2018-polio-png-en
2434	2018-07-06	https://www.who.int/emergencies/disease-outbreak-news/item/06-july-2018-ebola-drc-en
2435	2018-07-10	https://www.who.int/emergencies/disease-outbreak-news/item/10-july-2018-polio-drc-en
2436	2018-07-25	https://www.who.int/emergencies/disease-outbreak-news/item/25-july-2018-ebola-drc-en
2437	2018-08-04	https://www.who.int/emergencies/disease-outbreak-news/item/4-august-2018-ebola-drc-en
2438	2018-08-07	https://www.who.int/emergencies/disease-outbreak-news/item/07-august-2018-nipah-virus-india-en
2439	2018-08-08	https://www.who.int/emergencies/disease-outbreak-news/item/08-august-2018-polio-nigeria-en
2440	2018-08-09	https://www.who.int/emergencies/disease-outbreak-news/item/9-august-2018-ebola-drc-en
2441	2018-08-14	https://www.who.int/emergencies/disease-outbreak-news/item/14-august-2018-ebola-drc-en
2442	2018-08-17	https://www.who.int/emergencies/disease-outbreak-news/item/17-august-2018-ebola-drc-en
2443	2018-08-24	https://www.who.int/emergencies/disease-outbreak-news/item/24-august-2018-ebola-drc-en
2444	2018-08-24	https://www.who.int/emergencies/disease-outbreak-news/item/24-august-2018-yellow-fever-french-guiana-en
2445	2018-08-31	https://www.who.int/emergencies/disease-outbreak-news/item/31-august-2018-ebola-drc-en
2446	2018-08-31	https://www.who.int/emergencies/disease-outbreak-news/item/31-august-2018-mers-united-kingdom-en
2447	2018-09-05	https://www.who.int/emergencies/disease-outbreak-news/item/05-september-2018-ah7n9-china-en
2448	2018-09-07	https://www.who.int/emergencies/disease-outbreak-news/item/7-september-2018-ebola-drc-en
2449	2018-09-07	https://www.who.int/emergencies/disease-outbreak-news/item/7-september-2018-yellow-fever-congo-en
2450	2018-09-12	https://www.who.int/emergencies/disease-outbreak-news/item/12-september-2018-mers-republic-of-korea-en
2451	2018-09-14	https://www.who.int/emergencies/disease-outbreak-news/item/14-september-2018-cholera-algeria-en
2452	2018-09-14	https://www.who.int/emergencies/disease-outbreak-news/item/14-september-2018-ebola-drc-en
2453	2018-09-20	https://www.who.int/emergencies/disease-outbreak-news/item/20-september-2018-cholera-zimbabwe-en
2454	2018-09-20	https://www.who.int/emergencies/disease-outbreak-news/item/20-september-2018-ebola-drc-en
2455	2018-09-27	https://www.who.int/emergencies/disease-outbreak-news/item/27-september-2018-ebola-drc-en
2456	2018-10-03	https://www.who.int/emergencies/disease-outbreak-news/item/03-october-2018-mers-saudi-arabia-en
2457	2018-10-04	https://www.who.int/emergencies/disease-outbreak-news/item/04-october-2018-ebola-drc-en
2458	2018-10-05	https://www.who.int/emergencies/disease-outbreak-news/item/05-october-2018-cholera-niger-en
2459	2018-10-05	https://www.who.int/emergencies/disease-outbreak-news/item/05-october-2018-cholera-zimbabwe-en
2460	2018-10-05	https://www.who.int/emergencies/disease-outbreak-news/item/05-october-2018-monkeypox-nigeria-en
2461	2018-10-11	https://www.who.int/emergencies/disease-outbreak-news/item/11-october-2018-ebola-drc-en
2462	2018-10-15	https://www.who.int/emergencies/disease-outbreak-news/item/15-october-2018-chikungunya-sudan-en
2463	2018-10-18	https://www.who.int/emergencies/disease-outbreak-news/item/18-october-2018-ebola-drc-en
2464	2018-10-25	https://www.who.int/emergencies/disease-outbreak-news/item/25-october-2018-ebola-drc-en

2465	2018-10-30	https://www.who.int/emergencies/disease-outbreak-news/item/30-october-2018-polio-niger-en
2466	2018-11-01	https://www.who.int/emergencies/disease-outbreak-news/item/01-november-2018-ebola-drc-en
2467	2018-11-01	https://www.who.int/emergencies/disease-outbreak-news/item/01-november-2018-mers-saudi-arabia-en
2468	2018-11-08	https://www.who.int/emergencies/disease-outbreak-news/item/08-november-2018-ebola-drc-en
2469	2018-11-15	https://www.who.int/emergencies/disease-outbreak-news/item/15-november-2018-ebola-drc-en
2470	2018-11-20	https://www.who.int/emergencies/disease-outbreak-news/item/20-november-2018-mers-saudi-arabia-en
2471	2018-11-22	https://www.who.int/emergencies/disease-outbreak-news/item/22-november-2018-ebola-drc-en
2472	2018-11-29	https://www.who.int/emergencies/disease-outbreak-news/item/29-november-2018-ebola-drc-en
2473	2018-12-06	https://www.who.int/emergencies/disease-outbreak-news/item/06-December-2018-ebola-drc-en
2474	2018-12-13	https://www.who.int/emergencies/disease-outbreak-news/item/13-December-2018-ebola-drc-en
2475	2018-12-18	https://www.who.int/emergencies/disease-outbreak-news/item/18-December-2018-yellowfever-netherlands-en
2476	2018-12-20	https://www.who.int/emergencies/disease-outbreak-news/item/20-december-2018-ebola-drc-en
2477	2018-12-27	https://www.who.int/emergencies/disease-outbreak-news/item/27-december-2018-typhoid-pakistan-en
2478	2018-12-28	https://www.who.int/emergencies/disease-outbreak-news/item/28-december-2018-ebola-drc-en
2479	2018-12-28	https://www.who.int/emergencies/disease-outbreak-news/item/28-december-2018-mers-saudi-arabia-en
2480	2019-01-04	https://www.who.int/emergencies/disease-outbreak-news/item/04-January-2019-hantavirus-panama-en
2481	2019-01-04	https://www.who.int/emergencies/disease-outbreak-news/item/2019-DON113
2482	2019-01-08	https://www.who.int/emergencies/disease-outbreak-news/item/08-january-2019-poliovirus-drc-en
2483	2019-01-09	https://www.who.int/emergencies/disease-outbreak-news/item/09-january-2019-yellow-fever-nigeria-en
2484	2019-01-10	https://www.who.int/emergencies/disease-outbreak-news/item/2019-DON114
2485	2019-01-16	https://www.who.int/emergencies/disease-outbreak-news/item/2019-DON999
2486	2019-01-17	https://www.who.int/emergencies/disease-outbreak-news/item/17-january-2019-measles-madagascar-en
2487	2019-01-17	https://www.who.int/emergencies/disease-outbreak-news/item/2019-DON119
2488	2019-01-23	https://www.who.int/emergencies/disease-outbreak-news/item/23-January-2019-hantavirus-argentina-en
2489	2019-01-24	https://www.who.int/emergencies/disease-outbreak-news/item/2019-DON121
2490	2019-01-25	https://www.who.int/emergencies/disease-outbreak-news/item/25-january-2019-polio-mozambique-en
2491	2019-01-30	https://www.who.int/emergencies/disease-outbreak-news/item/30-january-2019-gonococcal-infection-uk-en
2492	2019-01-31	https://www.who.int/emergencies/disease-outbreak-news/item/2019-DON125
2493	2019-02-04	https://www.who.int/emergencies/disease-outbreak-news/item/4-february-2019-dengue-jamaica-en
2494	2019-02-07	https://www.who.int/emergencies/disease-outbreak-news/item/2019-DON128
2495	2019-02-11	https://www.who.int/emergencies/disease-outbreak-news/item/11-february-2019-mers-oman-en
2496	2019-02-11	https://www.who.int/emergencies/disease-outbreak-news/item/11-february-2019-yellow-fever-brazil-en
2497	2019-02-14	https://www.who.int/emergencies/disease-outbreak-news/item/14-february-2019-lassa-fever-nigeria-en
2498	2019-02-14	https://www.who.int/emergencies/disease-outbreak-news/item/2019-DON168
2499	2019-02-15	https://www.who.int/emergencies/disease-outbreak-news/item/2019-DON170

2500	2019-02-20	https://www.who.int/emergencies/disease-outbreak-news/item/20-February-2019-polio-png-en
2501	2019-02-21	https://www.who.int/emergencies/disease-outbreak-news/item/2019-DON131
2502	2019-02-26	https://www.who.int/emergencies/disease-outbreak-news/item/2019-DON132
2503	2019-02-27	https://www.who.int/emergencies/disease-outbreak-news/item/27-february-2019-polio-indonesia-en
2504	2019-02-28	https://www.who.int/emergencies/disease-outbreak-news/item/2019-DON134
2505	2019-03-04	https://www.who.int/emergencies/disease-outbreak-news/item/04-march-2019-mers-oman-en
2506	2019-03-05	https://www.who.int/emergencies/disease-outbreak-news/item/5-march-2019-carbapenem-resistant-p-aeruginosa-mex-en
2507	2019-03-07	https://www.who.int/emergencies/disease-outbreak-news/item/2019-DON136
2508	2019-03-14	https://www.who.int/emergencies/disease-outbreak-news/item/2019-DON137
2509	2019-03-21	https://www.who.int/emergencies/disease-outbreak-news/item/2019-DON138
2510	2019-03-28	https://www.who.int/emergencies/disease-outbreak-news/item/2019-DON141
2511	2019-03-29	https://www.who.int/emergencies/disease-outbreak-news/item/2019-DON139
2512	2019-04-04	https://www.who.int/emergencies/disease-outbreak-news/item/2019-DON142
2513	2019-04-11	https://www.who.int/emergencies/disease-outbreak-news/item/11-april-2019-ebola-drc-en
2514	2019-04-18	https://www.who.int/emergencies/disease-outbreak-news/item/18-april-2019-yellow-fever-brazil-en
2515	2019-04-18	https://www.who.int/emergencies/disease-outbreak-news/item/2019-DON147
2516	2019-04-24	https://www.who.int/emergencies/disease-outbreak-news/item/2019-DON145
2517	2019-04-25	https://www.who.int/emergencies/disease-outbreak-news/item/2019-DON149
2518	2019-05-01	https://www.who.int/emergencies/disease-outbreak-news/item/01-may-2019-chikungunya-congo-en
2519	2019-05-02	https://www.who.int/emergencies/disease-outbreak-news/item/2019-DON151
2520	2019-05-06	https://www.who.int/emergencies/disease-outbreak-news/item/2019-DON140
2521	2019-05-07	https://www.who.int/emergencies/disease-outbreak-news/item/07-may-2019-measles-western-pacific-region-en
2522	2019-05-09	https://www.who.int/emergencies/disease-outbreak-news/item/2019-DON150
2523	2019-05-09	https://www.who.int/emergencies/disease-outbreak-news/item/2019-DON152
2524	2019-05-09	https://www.who.int/emergencies/disease-outbreak-news/item/2019-DON169
2525	2019-05-13	https://www.who.int/emergencies/disease-outbreak-news/item/13-may-2019-rift-valley-fever-mayotte-france-en
2526	2019-05-16	https://www.who.int/emergencies/disease-outbreak-news/item/16-may-2019-ebola-drc-en
2527	2019-05-16	https://www.who.int/emergencies/disease-outbreak-news/item/16-may-2019-monkeypox-singapore-en
2528	2019-05-17	https://www.who.int/emergencies/disease-outbreak-news/item/17-may-2019-mers-saudi-arabia-en
2529	2019-05-20	https://www.who.int/emergencies/disease-outbreak-news/item/20-may-2019-dengue-reunion-en
2530	2019-05-23	https://www.who.int/emergencies/disease-outbreak-news/item/23-may-2019-ebola-drc-en
2531	2019-05-24	https://www.who.int/emergencies/disease-outbreak-news/item/24-may-2019-wild-polio-virus-islamic-republic-of-iran-en
2532	2019-05-30	https://www.who.int/emergencies/disease-outbreak-news/item/30-may-2019-ebola-drc-en
2533	2019-06-06	https://www.who.int/emergencies/disease-outbreak-news/item/06-june-2019-ebola-drc-en
2534	2019-06-06	https://www.who.int/emergencies/disease-outbreak-news/item/06-june-2019-polio-cameroon-en
2535	2019-06-13	https://www.who.int/emergencies/disease-outbreak-news/item/13-june-2019-ebola-drc-en

2536	2019-06-13	https://www.who.int/emergencies/disease-outbreak-news/item/13-june-2019-ebola-uganda-en
2537	2019-06-20	https://www.who.int/emergencies/disease-outbreak-news/item/2019-DON166
2538	2019-06-27	https://www.who.int/emergencies/disease-outbreak-news/item/2019-DON319
2539	2019-07-03	https://www.who.int/emergencies/disease-outbreak-news/item/2019-DON163
2540	2019-07-04	https://www.who.int/emergencies/disease-outbreak-news/item/2019-DON172
2541	2019-07-11	https://www.who.int/emergencies/disease-outbreak-news/item/2019-DON176
2542	2019-07-16	https://www.who.int/emergencies/disease-outbreak-news/item/2019-DON173
2543	2019-07-18	https://www.who.int/emergencies/disease-outbreak-news/item/2019-DON178
2544	2019-07-24	https://www.who.int/emergencies/disease-outbreak-news/item/2019-DON177
2545	2019-07-25	https://www.who.int/emergencies/disease-outbreak-news/item/2019-DON179
2546	2019-07-31	https://www.who.int/emergencies/disease-outbreak-news/item/2019-DON180
2547	2019-08-01	https://www.who.int/emergencies/disease-outbreak-news/item/2019-DON320
2548	2019-08-08	https://www.who.int/emergencies/disease-outbreak-news/item/2019-DON181
2549	2019-08-15	https://www.who.int/emergencies/disease-outbreak-news/item/2019-DON182
2550	2019-08-22	https://www.who.int/emergencies/disease-outbreak-news/item/2019-DON183
2551	2019-08-22	https://www.who.int/emergencies/disease-outbreak-news/item/2019-DON185
2552	2019-08-26	https://www.who.int/emergencies/disease-outbreak-news/item/2019-DON184
2553	2019-08-29	https://www.who.int/emergencies/disease-outbreak-news/item/2019-DON187
2554	2019-09-06	https://www.who.int/emergencies/disease-outbreak-news/item/2019-DON186
2555	2019-09-06	https://www.who.int/emergencies/disease-outbreak-news/item/2019-DON321
2556	2019-09-12	https://www.who.int/emergencies/disease-outbreak-news/item/2019-DON323
2557	2019-09-16	https://www.who.int/emergencies/disease-outbreak-news/item/2019-DON256
2558	2019-09-19	https://www.who.int/emergencies/disease-outbreak-news/item/2019-DON324
2559	2019-09-21	https://www.who.int/emergencies/disease-outbreak-news/item/2019-DON188
2560	2019-09-24	https://www.who.int/emergencies/disease-outbreak-news/item/2019-DON190
2561	2019-09-26	https://www.who.int/emergencies/disease-outbreak-news/item/2019-DON189
2562	2019-09-26	https://www.who.int/emergencies/disease-outbreak-news/item/2019-DON191
2563	2019-09-26	https://www.who.int/emergencies/disease-outbreak-news/item/2019-DON193
2564	2019-10-03	https://www.who.int/emergencies/disease-outbreak-news/item/2019-DON194
2565	2019-10-08	https://www.who.int/emergencies/disease-outbreak-news/item/2019-DON403
2566	2019-10-10	https://www.who.int/emergencies/disease-outbreak-news/item/2019-DON197
2567	2019-10-15	https://www.who.int/emergencies/disease-outbreak-news/item/2019-DON192
2568	2019-10-17	https://www.who.int/emergencies/disease-outbreak-news/item/2019-DON198
2569	2019-10-18	https://www.who.int/emergencies/disease-outbreak-news/item/2019-DON196
2570	2019-10-22	https://www.who.int/emergencies/disease-outbreak-news/item/2019-DON195
2571	2019-10-23	https://www.who.int/emergencies/disease-outbreak-news/item/2019-DON199
2572	2019-10-24	https://www.who.int/emergencies/disease-outbreak-news/item/2019-DON202
2573	2019-10-31	https://www.who.int/emergencies/disease-outbreak-news/item/2019-DON200
2574	2019-10-31	https://www.who.int/emergencies/disease-outbreak-news/item/2019-DON203

2575	2019-11-01	https://www.who.int/emergencies/disease-outbreak-news/item/2019-DON201
2576	2019-11-07	https://www.who.int/emergencies/disease-outbreak-news/item/2019-DON204
2577	2019-11-14	https://www.who.int/emergencies/disease-outbreak-news/item/2019-DON205
2578	2019-11-14	https://www.who.int/emergencies/disease-outbreak-news/item/2019-DON257
2579	2019-11-19	https://www.who.int/emergencies/disease-outbreak-news/item/2019-DON206
2580	2019-11-21	https://www.who.int/emergencies/disease-outbreak-news/item/2019-DON208
2581	2019-11-21	https://www.who.int/emergencies/disease-outbreak-news/item/2019-DON209
2582	2019-11-22	https://www.who.int/emergencies/disease-outbreak-news/item/2019-DON207
2583	2019-11-27	https://www.who.int/emergencies/disease-outbreak-news/item/2019-DON211
2584	2019-11-28	https://www.who.int/emergencies/disease-outbreak-news/item/2019-DON210
2585	2019-11-28	https://www.who.int/emergencies/disease-outbreak-news/item/2019-DON213
2586	2019-11-28	https://www.who.int/emergencies/disease-outbreak-news/item/2019-DON215
2587	2019-11-29	https://www.who.int/emergencies/disease-outbreak-news/item/2019-DON212
2588	2019-11-29	https://www.who.int/emergencies/disease-outbreak-news/item/2019-DON214
2589	2019-12-05	https://www.who.int/emergencies/disease-outbreak-news/item/2019-DON216
2590	2019-12-05	https://www.who.int/emergencies/disease-outbreak-news/item/2019-DON224
2591	2019-12-12	https://www.who.int/emergencies/disease-outbreak-news/item/2019-DON225
2592	2019-12-13	https://www.who.int/emergencies/disease-outbreak-news/item/2019-DON218
2593	2019-12-15	https://www.who.int/emergencies/disease-outbreak-news/item/2019-DON217
2594	2019-12-17	https://www.who.int/emergencies/disease-outbreak-news/item/2019-DON219
2595	2019-12-18	https://www.who.int/emergencies/disease-outbreak-news/item/2019-DON222
2596	2019-12-19	https://www.who.int/emergencies/disease-outbreak-news/item/2019-DON226
2597	2019-12-26	https://www.who.int/emergencies/disease-outbreak-news/item/2019-DON223
2598	2019-12-26	https://www.who.int/emergencies/disease-outbreak-news/item/2019-DON227
2599	2020-01-02	https://www.who.int/emergencies/disease-outbreak-news/item/2020-DON228
2600	2020-01-05	https://www.who.int/emergencies/disease-outbreak-news/item/2020-DON229
2601	2020-01-08	https://www.who.int/emergencies/disease-outbreak-news/item/2020-DON231
2602	2020-01-09	https://www.who.int/emergencies/disease-outbreak-news/item/2020-DON230
2603	2020-01-10	https://www.who.int/emergencies/disease-outbreak-news/item/2020-DON232
2604	2020-01-12	https://www.who.int/emergencies/disease-outbreak-news/item/2020-DON233
2605	2020-01-14	https://www.who.int/emergencies/disease-outbreak-news/item/2020-DON234
2606	2020-01-16	https://www.who.int/emergencies/disease-outbreak-news/item/2020-DON235
2607	2020-01-16	https://www.who.int/emergencies/disease-outbreak-news/item/2020-DON236
2608	2020-01-17	https://www.who.int/emergencies/disease-outbreak-news/item/2020-DON237
2609	2020-01-21	https://www.who.int/emergencies/disease-outbreak-news/item/2020-DON238
2610	2020-01-23	https://www.who.int/emergencies/disease-outbreak-news/item/2020-DON241
2611	2020-01-30	https://www.who.int/emergencies/disease-outbreak-news/item/2020-DON242
2612	2020-01-31	https://www.who.int/emergencies/disease-outbreak-news/item/2020-DON239
2613	2020-02-06	https://www.who.int/emergencies/disease-outbreak-news/item/2020-DON243

2614	2020-02-13	https://www.who.int/emergencies/disease-outbreak-news/item/2020-DON244
2615	2020-02-20	https://www.who.int/emergencies/disease-outbreak-news/item/2020-DON245
2616	2020-02-20	https://www.who.int/emergencies/disease-outbreak-news/item/2020-DON248
2617	2020-02-21	https://www.who.int/emergencies/disease-outbreak-news/item/2020-DON249
2618	2020-02-22	https://www.who.int/emergencies/disease-outbreak-news/item/2020-DON250
2619	2020-02-24	https://www.who.int/emergencies/disease-outbreak-news/item/2020-DON247
2620	2020-02-27	https://www.who.int/emergencies/disease-outbreak-news/item/2020-DON252
2621	2020-03-04	https://www.who.int/emergencies/disease-outbreak-news/item/2020-DON246
2622	2020-03-05	https://www.who.int/emergencies/disease-outbreak-news/item/2020-DON253
2623	2020-03-10	https://www.who.int/emergencies/disease-outbreak-news/item/2020-DON251
2624	2020-03-12	https://www.who.int/emergencies/disease-outbreak-news/item/2020-DON254
2625	2020-03-12	https://www.who.int/emergencies/disease-outbreak-news/item/2020-DON255
2626	2020-03-19	https://www.who.int/emergencies/disease-outbreak-news/item/2020-DON258
2627	2020-03-26	https://www.who.int/emergencies/disease-outbreak-news/item/2020-DON259
2628	2020-04-02	https://www.who.int/emergencies/disease-outbreak-news/item/2020-DON260
2629	2020-04-08	https://www.who.int/emergencies/disease-outbreak-news/item/2020-DON261
2630	2020-04-09	https://www.who.int/emergencies/disease-outbreak-news/item/2020-DON266
2631	2020-04-16	https://www.who.int/emergencies/disease-outbreak-news/item/2020-DON268
2632	2020-04-18	https://www.who.int/emergencies/disease-outbreak-news/item/2020-DON262
2633	2020-04-22	https://www.who.int/emergencies/disease-outbreak-news/item/2020-DON263
2634	2020-04-23	https://www.who.int/emergencies/disease-outbreak-news/item/2020-DON265
2635	2020-04-23	https://www.who.int/emergencies/disease-outbreak-news/item/2020-DON269
2636	2020-04-24	https://www.who.int/emergencies/disease-outbreak-news/item/2020-DON267
2637	2020-04-30	https://www.who.int/emergencies/disease-outbreak-news/item/2020-DON271
2638	2020-05-05	https://www.who.int/emergencies/disease-outbreak-news/item/2020-DON270
2639	2020-05-06	https://www.who.int/emergencies/disease-outbreak-news/item/2020-DON264
2640	2020-05-07	https://www.who.int/emergencies/disease-outbreak-news/item/2020-DON272
2641	2020-05-14	https://www.who.int/emergencies/disease-outbreak-news/item/2020-DON274
2642	2020-05-21	https://www.who.int/emergencies/disease-outbreak-news/item/2020-DON275
2643	2020-05-25	https://www.who.int/emergencies/disease-outbreak-news/item/2020-DON273
2644	2020-05-28	https://www.who.int/emergencies/disease-outbreak-news/item/2020-DON276
2645	2020-06-03	https://www.who.int/emergencies/disease-outbreak-news/item/2020-DON277
2646	2020-06-04	https://www.who.int/emergencies/disease-outbreak-news/item/2020-DON278
2647	2020-06-05	https://www.who.int/emergencies/disease-outbreak-news/item/2020-DON279
2648	2020-06-11	https://www.who.int/emergencies/disease-outbreak-news/item/2020-DON281
2649	2020-06-17	https://www.who.int/emergencies/disease-outbreak-news/item/2020-DON282
2650	2020-06-18	https://www.who.int/emergencies/disease-outbreak-news/item/2020-DON283
2651	2020-06-26	https://www.who.int/emergencies/disease-outbreak-news/item/2020-DON284
2652	2020-07-02	https://www.who.int/emergencies/disease-outbreak-news/item/2020-DON285

2653	2020-07-09	https://www.who.int/emergencies/disease-outbreak-news/item/2020-DON286
2654	2020-07-23	https://www.who.int/emergencies/disease-outbreak-news/item/plague-democratic-republic-of-the-congo
2655	2020-08-01	https://www.who.int/emergencies/disease-outbreak-news/item/2020-DON288
2656	2020-09-01	https://www.who.int/emergencies/disease-outbreak-news/item/circulating-vaccine-derived-poliovirus-type-2-sudan
2657	2020-09-03	https://www.who.int/emergencies/disease-outbreak-news/item/ebola-virus-disease-democratic-republic-of-the-congo
2658	2020-09-24	https://www.who.int/emergencies/disease-outbreak-news/item/chikungunya-chad
2659	2020-10-01	https://www.who.int/emergencies/disease-outbreak-news/item/monkeypox-democratic-republic-of-the-congo
2660	2020-10-13	https://www.who.int/emergencies/disease-outbreak-news/item/oropouche-virus-disease---french-guiana-france
2661	2020-10-24	https://www.who.int/emergencies/disease-outbreak-news/item/mayaro-virus-disease---french-guiana-france
2662	2020-11-06	https://www.who.int/emergencies/disease-outbreak-news/item/2020-DON297
2663	2020-11-13	https://www.who.int/emergencies/disease-outbreak-news/item/rift-valley-fever-mauritania
2664	2020-11-17	https://www.who.int/emergencies/disease-outbreak-news/item/avian-influenza-a(h5n1)--lao-people-s-democratic-republic
2665	2020-11-18	https://www.who.int/emergencies/disease-outbreak-news/item/ebola-virus-disease-democratic-republic-of-the-congo-draft
2666	2020-11-24	https://www.who.int/emergencies/disease-outbreak-news/item/2020-DON299
2667	2020-11-27	https://www.who.int/emergencies/disease-outbreak-news/item/2020-DON300
2668	2020-12-03	https://www.who.int/emergencies/disease-outbreak-news/item/2020-DON301
2669	2020-12-21	https://www.who.int/emergencies/disease-outbreak-news/item/2020-DON304
2670	2020-12-23	https://www.who.int/emergencies/disease-outbreak-news/item/2020-DON302
2671	2020-12-29	https://www.who.int/emergencies/disease-outbreak-news/item/2020-DON303
2672	2020-12-31	https://www.who.int/emergencies/disease-outbreak-news/item/2020-DON305
2673	2021-01-04	https://www.who.int/emergencies/disease-outbreak-news/item/2021-DON306
2674	2021-01-04	https://www.who.int/emergencies/disease-outbreak-news/item/cholera-togo
2675	2021-02-01	https://www.who.int/emergencies/disease-outbreak-news/item/2021-DON308
2676	2021-02-05	https://www.who.int/emergencies/disease-outbreak-news/item/2021-DON309
2677	2021-02-10	https://www.who.int/emergencies/disease-outbreak-news/item/2021-DON310
2678	2021-02-12	https://www.who.int/emergencies/disease-outbreak-news/item/2021-DON311
2679	2021-02-17	https://www.who.int/emergencies/disease-outbreak-news/item/2021-DON312
2680	2021-02-26	https://www.who.int/emergencies/disease-outbreak-news/item/2021-DON313
2681	2021-03-17	https://www.who.int/emergencies/disease-outbreak-news/item/2021-DON314
2682	2021-03-26	https://www.who.int/emergencies/disease-outbreak-news/item/circulating-vaccine-derived-poliovirus-type-2-global-update
2683	2021-04-14	https://www.who.int/emergencies/disease-outbreak-news/item/2021-DON317
2684	2021-05-04	https://www.who.int/emergencies/disease-outbreak-news/item/2021-DON325
2685	2021-06-10	https://www.who.int/emergencies/disease-outbreak-news/item/human-infection-with-avian-influenza-a(h10n3)-china
2686	2021-06-11	https://www.who.int/emergencies/disease-outbreak-news/item/monkeypox---united-kingdom-of-great-britain-and-northern-ireland-ex-nigeria
2687	2021-06-19	https://www.who.int/emergencies/disease-outbreak-news/item/2021-DON328

2688	2021-07-08	https://www.who.int/emergencies/disease-outbreak-news/item/monkeypox---united-kingdom-of-great-britain-and-northern-ireland
2689	2021-07-27	https://www.who.int/emergencies/disease-outbreak-news/item/monkeypox---the-united-states-of-america
2690	2021-08-09	https://www.who.int/emergencies/disease-outbreak-news/item/2021-DON331
2691	2021-08-16	https://www.who.int/emergencies/disease-outbreak-news/item/human-infection-with-avian-influenza-a(h5n1)-%EF%BD%B0-india
2692	2021-08-17	https://www.who.int/emergencies/disease-outbreak-news/item/2021-DON333
2693	2021-09-17	https://www.who.int/emergencies/disease-outbreak-news/item/marburg-virus-disease---guinea
2694	2021-09-20	https://www.who.int/emergencies/disease-outbreak-news/item/2021-DON334
2695	2021-09-24	https://www.who.int/emergencies/disease-outbreak-news/item/nipah-virus-disease---india
2696	2021-10-01	https://www.who.int/emergencies/disease-outbreak-news/item/2021-DON336
2697	2021-10-01	https://www.who.int/emergencies/disease-outbreak-news/item/plague---madagascar
2698	2021-10-10	https://www.who.int/emergencies/disease-outbreak-news/item/ebola-virus-disease-democratic-republic-of-the-congo_1
2699	2021-10-13	https://www.who.int/emergencies/disease-outbreak-news/item/yellow-fever---bolivarian-republic-of-venezuela
2700	2021-10-14	https://www.who.int/emergencies/disease-outbreak-news/item/zika-virus-disease-india
2701	2021-10-22	https://www.who.int/emergencies/disease-outbreak-news/item/circulating-vaccine-derived-poliovirus-type-2-(cvdpv2)-ukraine
2702	2021-11-25	https://www.who.int/emergencies/disease-outbreak-news/item/2021-DON344
2703	2021-12-01	https://www.who.int/emergencies/disease-outbreak-news/item/yellow-fever---ghana
2704	2021-12-09	https://www.who.int/emergencies/disease-outbreak-news/item/circulating-vaccine-derived-poliovirus-type-2-(cvdpv2)-yemen
2705	2021-12-13	https://www.who.int/emergencies/disease-outbreak-news/item/middle-east-respiratory-syndrome-coronavirus-(mers-cov)-united-arab-emirates
2706	2021-12-14	https://www.who.int/emergencies/disease-outbreak-news/item/dengue-fever-pakistan
2707	2021-12-16	https://www.who.int/emergencies/disease-outbreak-news/item/2021-DON351
2708	2021-12-16	https://www.who.int/emergencies/disease-outbreak-news/item/cholera-cameroon
2709	2021-12-23	https://www.who.int/emergencies/disease-outbreak-news/item/hepatitis-e-virus-republic-of-south-sudan
2710	2021-12-23	https://www.who.int/emergencies/disease-outbreak-news/item/yellow-fever---west-and-central-africa
2711	2022-01-14	https://www.who.int/emergencies/disease-outbreak-news/item/influenza-a-(h5)---united-kingdom-of-great-britain-and-northern-ireland
2712	2022-01-17	https://www.who.int/emergencies/disease-outbreak-news/item/2022-DON352
2713	2022-01-25	https://www.who.int/emergencies/disease-outbreak-news/item/cholera-benin
2714	2022-02-04	https://www.who.int/emergencies/disease-outbreak-news/item/dengue---timor-leste
2715	2022-02-10	https://www.who.int/emergencies/disease-outbreak-news/item/measles-afghanistan
2716	2022-02-14	https://www.who.int/emergencies/disease-outbreak-news/item/lassa-fever---nigeria
2717	2022-02-21	https://www.who.int/emergencies/disease-outbreak-news/item/lassa-fever-united-kingdom-of-great-britain-and-northern-ireland
2718	2022-03-03	https://www.who.int/emergencies/disease-outbreak-news/item/wild-poliovirus-type-1-(WPV1)-malawi
2719	2022-03-24	https://www.who.int/emergencies/disease-outbreak-news/item/2022-DON362
2720	2022-03-24	https://www.who.int/emergencies/disease-outbreak-news/item/2022-DON364
2721	2022-03-25	https://www.who.int/emergencies/disease-outbreak-news/item/2022-DON361
2722	2022-04-07	https://www.who.int/emergencies/disease-outbreak-news/item/2022-DON363

2723	2022-04-15	https://www.who.int/emergencies/disease-outbreak-news/item/2022-DON366
2724	2022-04-15	https://www.who.int/emergencies/disease-outbreak-news/item/2022-DON368
2725	2022-04-23	https://www.who.int/emergencies/disease-outbreak-news/item/2022-DON376
2726	2022-04-25	https://www.who.int/emergencies/disease-outbreak-news/item/2022-DON367
2727	2022-04-27	https://www.who.int/emergencies/disease-outbreak-news/item/2022-DON369
2728	2022-04-27	https://www.who.int/emergencies/disease-outbreak-news/item/2022-DON371
2729	2022-04-27	https://www.who.int/emergencies/disease-outbreak-news/item/2022-DON372
2730	2022-04-28	https://www.who.int/emergencies/disease-outbreak-news/item/2022-DON365
2731	2022-04-28	https://www.who.int/emergencies/disease-outbreak-news/item/2022-DON377
2732	2022-05-06	https://www.who.int/emergencies/disease-outbreak-news/item/2022-DON379
2733	2022-05-09	https://www.who.int/emergencies/disease-outbreak-news/item/2022-DON378
2734	2022-05-12	https://www.who.int/emergencies/disease-outbreak-news/item/2022-DON370
2735	2022-05-13	https://www.who.int/emergencies/disease-outbreak-news/item/2022-DON382
2736	2022-05-16	https://www.who.int/emergencies/disease-outbreak-news/item/2022-DON374
2737	2022-05-16	https://www.who.int/emergencies/disease-outbreak-news/item/2022-DON381
2738	2022-05-17	https://www.who.int/emergencies/disease-outbreak-news/item/2022-DON380
2739	2022-05-18	https://www.who.int/emergencies/disease-outbreak-news/item/2022-DON383
2740	2022-05-19	https://www.who.int/emergencies/disease-outbreak-news/item/2022-DON384
2741	2022-05-21	https://www.who.int/emergencies/disease-outbreak-news/item/2022-DON385
2742	2022-05-26	https://www.who.int/emergencies/disease-outbreak-news/item/2022-DON387
2743	2022-05-27	https://www.who.int/emergencies/disease-outbreak-news/item/DON-389
2744	2022-05-29	https://www.who.int/emergencies/disease-outbreak-news/item/2022-DON388
2745	2022-06-01	https://www.who.int/emergencies/disease-outbreak-news/item/2022-DON386
2746	2022-06-04	https://www.who.int/emergencies/disease-outbreak-news/item/2022-DON390
2747	2022-06-10	https://www.who.int/emergencies/disease-outbreak-news/item/2022-DON392
2748	2022-06-17	https://www.who.int/emergencies/disease-outbreak-news/item/2022-DON391
2749	2022-06-17	https://www.who.int/emergencies/disease-outbreak-news/item/2022-DON393
2750	2022-06-23	https://www.who.int/emergencies/disease-outbreak-news/item/2022-DON395
2751	2022-06-24	https://www.who.int/emergencies/disease-outbreak-news/item/2022-DON394
2752	2022-06-27	https://www.who.int/emergencies/disease-outbreak-news/item/2022-DON396
2753	2022-07-04	https://www.who.int/emergencies/disease-outbreak-news/item/2022-DON398
2754	2022-07-12	https://www.who.int/emergencies/disease-outbreak-news/item/2022-DON400
2755	2022-07-20	https://www.who.int/emergencies/disease-outbreak-news/item/2022-DON398_1
2756	2022-07-22	https://www.who.int/emergencies/disease-outbreak-news/item/2022-DON402
2757	2022-08-03	https://www.who.int/emergencies/disease-outbreak-news/item/2022-DON401
2758	2022-08-12	https://www.who.int/emergencies/disease-outbreak-news/item/2022-DON403
2759	2022-08-25	https://www.who.int/emergencies/disease-outbreak-news/item/2022-DON404
2760	2022-09-02	https://www.who.int/emergencies/disease-outbreak-news/item/2022-DON405
2761	2022-09-05	https://www.who.int/emergencies/disease-outbreak-news/item/2022-DON407

2762	2022-09-13	https://www.who.int/emergencies/disease-outbreak-news/item/2022-DON406
2763	2022-09-14	https://www.who.int/emergencies/disease-outbreak-news/item/2022-DON408
2764	2022-09-26	https://www.who.int/emergencies/disease-outbreak-news/item/2022-DON409
2765	2022-09-26	https://www.who.int/emergencies/disease-outbreak-news/item/2022-DON410
2766	2022-09-29	https://www.who.int/emergencies/disease-outbreak-news/item/2022-DON411
2767	2022-10-10	https://www.who.int/emergencies/disease-outbreak-news/item/2022-DON412
2768	2022-10-12	https://www.who.int/emergencies/disease-outbreak-news/item/2022-DON415
2769	2022-10-13	https://www.who.int/emergencies/disease-outbreak-news/item/2022-DON414
2770	2022-10-17	https://www.who.int/emergencies/disease-outbreak-news/item/2022-DON413
2771	2022-10-19	https://www.who.int/emergencies/disease-outbreak-news/item/2022-DON416
2772	2022-10-20	https://www.who.int/emergencies/disease-outbreak-news/item/2022-DON417
2773	2022-10-28	https://www.who.int/emergencies/disease-outbreak-news/item/2022-DON421
2774	2022-11-03	https://www.who.int/emergencies/disease-outbreak-news/item/2022-DON420
2775	2022-11-07	https://www.who.int/emergencies/disease-outbreak-news/item/2022-DON419
2776	2022-11-10	https://www.who.int/emergencies/disease-outbreak-news/item/2022-DON423
2777	2022-11-16	https://www.who.int/emergencies/disease-outbreak-news/item/2022-DON422
2778	2022-11-24	https://www.who.int/emergencies/disease-outbreak-news/item/2022-DON425
2779	2022-11-28	https://www.who.int/emergencies/disease-outbreak-news/item/2022-DON424
2780	2022-12-08	https://www.who.int/emergencies/disease-outbreak-news/item/2022-DON428
2781	2022-12-13	https://www.who.int/emergencies/disease-outbreak-news/item/2022-DON427
2782	2022-12-15	https://www.who.int/emergencies/disease-outbreak-news/item/2022-DON429
2783	2022-12-16	https://www.who.int/emergencies/disease-outbreak-news/item/2022-DON426
2784	2022-12-19	https://www.who.int/emergencies/disease-outbreak-news/item/2022-DON430
2785	2023-01-03	https://www.who.int/emergencies/disease-outbreak-news/item/2022-DON431
2786	2023-01-12	https://www.who.int/emergencies/disease-outbreak-news/item/2023-DON362
2787	2023-01-14	https://www.who.int/emergencies/disease-outbreak-news/item/2023-DON433
2788	2023-01-18	https://www.who.int/emergencies/disease-outbreak-news/item/2023-DON434
2789	2023-02-02	https://www.who.int/emergencies/disease-outbreak-news/item/2023-DON438
2790	2023-02-08	https://www.who.int/emergencies/disease-outbreak-news/item/2023-DON436
2791	2023-02-08	https://www.who.int/emergencies/disease-outbreak-news/item/2023-DON439
2792	2023-02-09	https://www.who.int/emergencies/disease-outbreak-news/item/2022-DON435
2793	2023-02-10	https://www.who.int/emergencies/disease-outbreak-news/item/2023-DON440
2794	2023-02-10	https://www.who.int/emergencies/disease-outbreak-news/item/2023-DON441
2795	2023-02-11	https://www.who.int/emergencies/disease-outbreak-news/item/2023-DON437
2796	2023-02-17	https://www.who.int/emergencies/disease-outbreak-news/item/2023-DON442
2797	2023-02-24	https://www.who.int/emergencies/disease-outbreak-news/item/2023-DON443
2798	2023-02-25	https://www.who.int/emergencies/disease-outbreak-news/item/2023-DON444
2799	2023-02-26	https://www.who.int/emergencies/disease-outbreak-news/item/2023-DON445
2800	2023-03-14	https://www.who.int/emergencies/disease-outbreak-news/item/2023-DON446

2801	2023-03-21	https://www.who.int/emergencies/disease-outbreak-news/item/2023-DON447
2802	2023-03-22	https://www.who.int/emergencies/disease-outbreak-news/item/2023-DON449
2803	2023-03-23	https://www.who.int/emergencies/disease-outbreak-news/item/2023-DON448
2804	2023-03-24	https://www.who.int/emergencies/disease-outbreak-news/item/2023-DON450
2805	2023-03-24	https://www.who.int/emergencies/disease-outbreak-news/item/2023-DON451
2806	2023-04-06	https://www.who.int/emergencies/disease-outbreak-news/item/2023-DON453
2807	2023-04-11	https://www.who.int/emergencies/disease-outbreak-news/item/2023-DON455
2808	2023-04-11	https://www.who.int/emergencies/disease-outbreak-news/item/2023-DON456
2809	2023-04-15	https://www.who.int/emergencies/disease-outbreak-news/item/2023-DON459
2810	2023-04-17	https://www.who.int/emergencies/disease-outbreak-news/item/2023-DON458
2811	2023-04-20	https://www.who.int/emergencies/disease-outbreak-news/item/2023-DON457
2812	2023-04-21	https://www.who.int/emergencies/disease-outbreak-news/item/2023-DON461
2813	2023-04-27	https://www.who.int/emergencies/disease-outbreak-news/item/2023-DON452
2814	2023-04-27	https://www.who.int/emergencies/disease-outbreak-news/item/2023-DON454
2815	2023-04-28	https://www.who.int/emergencies/disease-outbreak-news/item/2023-DON462
2816	2023-05-01	https://www.who.int/emergencies/disease-outbreak-news/item/2023-DON463
2817	2023-05-04	https://www.who.int/emergencies/disease-outbreak-news/item/2023-DON460
2818	2023-05-05	https://www.who.int/emergencies/disease-outbreak-news/item/2023-DON466
2819	2023-05-08	https://www.who.int/emergencies/disease-outbreak-news/item/2023-DON467
2820	2023-05-17	https://www.who.int/emergencies/disease-outbreak-news/item/2023-DON465
2821	2023-05-30	https://www.who.int/emergencies/disease-outbreak-news/item/2023-DON468
2822	2023-05-31	https://www.who.int/emergencies/disease-outbreak-news/item/2023-DON469
2823	2023-06-01	https://www.who.int/emergencies/disease-outbreak-news/item/2023-DON470
2824	2023-06-02	https://www.who.int/emergencies/disease-outbreak-news/item/2023-DON471
2825	2023-06-09	https://www.who.int/emergencies/disease-outbreak-news/item/2023-DON472
2826	2023-06-16	https://www.who.int/emergencies/disease-outbreak-news/item/2023-DON473
2827	2023-07-07	https://www.who.int/emergencies/disease-outbreak-news/item/2023-DON474
2828	2023-07-16	https://www.who.int/emergencies/disease-outbreak-news/item/2023-DON476
2829	2023-07-19	https://www.who.int/emergencies/disease-outbreak-news/item/2023-DON475
2830	2023-07-24	https://www.who.int/emergencies/disease-outbreak-news/item/2023-DON478
2831	2023-07-25	https://www.who.int/emergencies/disease-outbreak-news/item/2023-DON477
2832	2023-07-28	https://www.who.int/emergencies/disease-outbreak-news/item/2023-DON479
2833	2023-07-28	https://www.who.int/emergencies/disease-outbreak-news/item/2023-DON480
2834	2023-08-11	https://www.who.int/emergencies/disease-outbreak-news/item/2023-DON481
2835	2023-08-11	https://www.who.int/emergencies/disease-outbreak-news/item/2023-DON482
2836	2023-08-23	https://www.who.int/emergencies/disease-outbreak-news/item/2023-DON483
2837	2023-08-29	https://www.who.int/emergencies/disease-outbreak-news/item/2023-DON484
2838	2023-09-13	https://www.who.int/emergencies/disease-outbreak-news/item/2023-DON485
2839	2023-09-13	https://www.who.int/emergencies/disease-outbreak-news/item/2023-DON486

2840	2023-09-14	https://www.who.int/emergencies/disease-outbreak-news/item/2023-DON487
2841	2023-09-20	https://www.who.int/emergencies/disease-outbreak-news/item/2023-DON489
2842	2023-09-21	https://www.who.int/emergencies/disease-outbreak-news/item/2023-DON488
2843	2023-10-03	https://www.who.int/emergencies/disease-outbreak-news/item/2023-DON490
2844	2023-10-16	https://www.who.int/emergencies/disease-outbreak-news/item/2023-DON491
2845	2023-10-18	https://www.who.int/emergencies/disease-outbreak-news/item/2023-DON492
2846	2023-11-23	https://www.who.int/emergencies/disease-outbreak-news/item/2023-DON493
2847	2023-11-23	https://www.who.int/emergencies/disease-outbreak-news/item/2023-DON494
2848	2023-11-29	https://www.who.int/emergencies/disease-outbreak-news/item/2023-DON495
2849	2023-12-01	https://www.who.int/emergencies/disease-outbreak-news/item/2023-DON496
2850	2023-12-08	https://www.who.int/emergencies/disease-outbreak-news/item/2023-DON497
2851	2023-12-21	https://www.who.int/emergencies/disease-outbreak-news/item/2023-DON498
2852	2023-12-28	https://www.who.int/emergencies/disease-outbreak-news/item/2023-DON499
2853	2024-01-11	https://www.who.int/emergencies/disease-outbreak-news/item/2024-DON500
2854	2024-02-07	https://www.who.int/emergencies/disease-outbreak-news/item/2024-DON502
2855	2024-02-08	https://www.who.int/zh/emergencies/disease-outbreak-news/item/2024-DON501
2856	2024-02-08	https://www.who.int/emergencies/disease-outbreak-news/item/2024-DON505

Table S2. Total number of reports of outbreaks, number of cases and deaths caused by the outbreaks (if reported), and case fatality rate of the outbreaks*.

**Outbreaks' name**	**Number of reported**	**Number of cases**	**Number of deaths**	**Case fatality rate (%)**
Respiratory Infections				
Influenza	771	3285450	577523	17.58
MERS-CoV	305	6418	1793	27.94
SARS	123	306203	22928	7.49
Measles	43	755799	2110	0.28
nCoV	24	327	147	44.95
Other respiratory syndromes	13	1230	21	1.71
Diphtheria	7	7969	188	2.36
Pneumonia†	3	1141	36	3.16
Pertussis	2	115	17	14.78
Coccidioidomycosis	1	1	–	–
XDR-TB	1	–	–	–
Vector-Borne Infections				
Yellow fever	164	779323	3434	0.44
Dengue	73	12977361	12327	0.09
Zika	47	317	0	0
Rift Valley Fever	38	7315	1959	26.78
Plague	36	10492	1004	9.57

Chikungunya	22	1311863	0	0
West Nile virus	21	8982	449	5
Hantavirus	6	533	23	4.32
Malaria	6	4020945	51	0
Tularemia	5	1743	0	0
Leishmaniasis	4	205738	–	–
Oropouche virus	2	94	0	0
Arenaviridae family	1	–	3	–
Mayaro fever	1	13	–	–
O'nyong'nyong Fever	1	–	–	–
Relapsing fever	1	–	–	–
Seoul virus	1	11	–	–
Foodborne or Waterborne Infections				
Cholera	289	4904288	149988	3.06
Poliovirus	112	5569	91	1.63
Diarrhea[†]	13	246619	504	0.2
Enterovirus	13	2056	260	12.65
Legionellosis	12	2189	63	2.88
typhoid fever	9	68228	135	0.2
Leptospirosis	6	1391	65	4.67
E.coli	5	15957	29	0.18
Listeriosis	5	2270	400	17.62
Shigella	5	2040	94	4.61
Salmonella/Salmonella Typhimurium	4	342	0	0
Botulism	4	92	1	1.09
food-borne intoxication	2	13914	–	–
Dracunculiasis	1	7	–	–
Dysentery	1	3094	132	4.27
Direct Contact Infections				
Ebola	342	226701	142813	63
Lassa fever	44	8934	1617	18.1
Marburg virus	28	795	611	76.86
Monkeypox	26	23062	840	3.64
Anthrax	18	932	42	4.51
Hand, Foot, and Mouth Disease	7	11041	232	2.1
Creutzfeldt-Jakob Disease	3	50	–	–
Streptococcus[†]	3	4828	51	1.06
Rabies	2	1	–	–
Buffalopox	1	5	–	–
Smallpox	1	8	–	–

Staphylococcus aureus	1	–	–	–
Surgical site infections caused by antibiotic-resistant Pseudomonas aeruginosa	1	20	1	5
Other outbreaks				
Meningitis	178	609772	60299	9.89
Haemorrhagic fever	68	8095	5151	63.63
Unknown	23	16678	1182	7.09
Acute hepatitis	18	16952	338	1.99
Encephalitis	12	4250	763	17.95
Nipah virus	8	94	55	58.51
Kwazulu-Natal	2	59167	146	0.25
Acute febrile illness	1	37	–	–
Borreliosis	1	–	–	–
Elizabethkingia anophelis	1	57	18	31.58
Hendra-like virus	1	15	1	6.67
HIV	1	30192	–	–
Reston virus	1	–	–	–
XDR Neisseria gonorrhoeae	1	2	–	–

CFR, case fatality rate; MERS-CoV, Middle East respiratory syndrome coronavirus; SARS, severe acute respiratory syndrome; nCoV, Novel Coronavirus; HIV, human immunodeficiency virus.

*The reported numbers of cases and deaths in this table represent cumulative counts aggregated from each outbreak report. For certain infectious diseases, multiple reports may document additional cases and deaths during the same outbreak, resulting in overlapping data being included in the totals. Consequently, the aggregated numbers of cases and deaths reflect all reported instances without de-duplication. This approach is appropriate for calculating the case fatality rate (CFR) since it is derived directly from the aggregated reported cases and deaths within the same data framework, ensuring consistency and comparability across outbreaks.

† Depending on the exact cause

Table S3. Top ten outbreaks by number of cases and deaths in different categories

Ranking	Total Number of cases				Total Number of Death			
	**Outbreak name**	**Year**	**Location**	**Number**	**Outbreak name**	**Year**	**Location**	**Number**
Respiratory Infections								
1	Influenza A (H1N1)	2009	Worldwide	414000	Influenza A (H1N1)	2010	Worldwide	>18449
2	Measles	2019	Worldwide	413308	Influenza A (H1N1)	2009	Worldwide	1799
3	Influenza-like illness	2009	Ukraine	250000	Measles	2011	DR Congo	>1100
4	Measles	2011	DR Congo	103000	SARS	2003	Worldwide	919
5	Measles	2022	Afghanistan	35319	Influenza A (H7N9)	2018	China	>615
6	Measles	2019	European Region	34300	Influenza-like illness	2002	Madagascar	444
7	Measles	2011	Europe	26025	Influenza A (H5N1)	2014	Worldwide	385
8	Measles	2019	Philippines	25956	Measles	2019	Philippines	381
9	Measles	2015	European Region	23000	MERS-CoV	2014	Worldwide	301
10	Measles	2019	Madagascar	19539	Measles	2023	Ethiopia	182
Vector-Borne Infections								
1	Dengue	2023	Worldwide	5000000	Dengue	2023	Worldwide	5000
2	Malaria	2022	Pakistan	3400000	Dengue	2023	Americas	1302
3	Chikungunya and Dengue	2023	Americas	3123752	Dengue	2004	Indonesia	658
4	Dengue	2023	Americas	2997097	Dengue	2008	Brazil	647
5	Chikungunya fever	2006	India	1250000	Dengue	2004	Java	507
6	Yellow fever	2013	Cameroon	663900	Dengue	1996	Delhi	354
7	malaria	1999	Burundi	616034	Dengue	1996	India	346
8	Dengue	1998	Asia	552088	Yellow fever	2016	Angola	345
9	Dengue	2002	Rio de Janeiro	317787	Dengue	2023	Bangladesh	327
10	Dengue	1996	Americas	200,000	Rift Valley fever	1998	Kenya	>300
Foodborne or Waterborne Infections								
1	Cholera	2023	Worldwide	2900000	Cholera	2023	Worldwide	95000
2	Diarrhoea	1998	Not reported	185000	Cholera	2009	Zimbabwe	4276
3	Cholera	2009	Zimbabwe	98424	Cholera	1996	Nigeria	2411

4	Cholera	2001	Kwazulu-Natal	86107	Cholera	1998	Tanzania	2025
5	Cholera	2009	Zimbabwe	79613	Cholera	2010	Central Africa	1879
6	Cholera	2010	Haiti	60,240	Cholera	1998	Uganda	1777
7	Cholera	2009	Zimbabwe	>60000	Cholera	2006	Angola	1642
8	Cholera	1998	Uganda	43911	Cholera	1998	Uganda	1,576
9	Cholera	2006	Angola	43076	Cholera	2008	Zimbabwe	1518
10	Cholera	2010	Central Africa	40468	Cholera	2006	Angola	1437
Direct Contact Infections								
1	Monkeypox	2023	DR Congo	12569	Ebola	2020	DR Congo	2287
2	Streptococcus	2022	Europe	4622	Ebola	2019	DR Congo	2217
3	HFMD	2008	China	4496	Ebola	2019	DR Congo	833
4	HFMD	1997	Malaysia	4253	Ebola	2019	Beni	383
5	Ebola	2020	DR Congo	3470	Ebola	2018	DR Congo	357
6	Monkeypox	2022	Worldwide	3413	Marburg virus	2005	Angola	329
7	Ebola	2000	Kwazulu-Natal	3279	Ebola	2014	Guinea	270
8	Monkeypox	2021	DR Congo	2780	Ebola	2001	Uganda	268
9	Monkeypox	2022	Worldwide	2103	Ebola	2014	Guinea	267
10	Lassa fever	2018	Nigeria	1849	Ebola	2018	DR Congo	219
Other Outbreaks								
1	Meningococcal disease	2002	Somalia	67681	Cerebrospinal meningitis	1996	African Region	8955
2	Cerebrospinal meningitis	1996	African Region	62280	Meningitis	1997	African Region	4,498
3	Kwazulu-Natal	2001	South Africa	56092	Meningitis	1997	Multiple countries	2933
4	Meningitis	1996	African Region	51,541	Cerebrospinal meningitis	1996	Nigeria	2550
5	Meningitis	1997	African Region	41,699	Meningococcal disease	1999	Sudan	1,600
6	Cerebrospinal meningitis	1996	African Region	>38000	Meningococcal disease	2001	Burkina Faso	1525
7	HIV	2019	Pakistan	30,192	Meningitis	2007	Burkina Faso	1490
8	Meningitis	1997	Multiple countries	24798	Meningitis	2002	Burkina Faso	1447
9	Meningitis	2007	Burkina Faso	22255	Meningitis	1996	Nigeria	1,181
10	Meningococcal disease	1999	Sudan	22,000	Meningococcal disease	2001	Burkina Faso	1137

SARS, Severe Acute Respiratory Syndrome; HFMD, Hand, foot and mouth disease; HIV, human immunodeficiency virus
